# A transdisciplinary and community-driven database to unravel subduction zone initiation

**DOI:** 10.1038/s41467-020-17522-9

**Published:** 2020-07-27

**Authors:** Fabio Crameri, Valentina Magni, Mathew Domeier, Grace E. Shephard, Kiran Chotalia, George Cooper, Caroline M. Eakin, Antoniette Greta Grima, Derya Gürer, Ágnes Király, Elvira Mulyukova, Kalijn Peters, Boris Robert, Marcel Thielmann

**Affiliations:** 1 Centre for Earth Evolution and Dynamics (CEED), University of Oslo, Oslo, Norway; 2 Department of Earth Sciences, University College London, London, UK; 3 School of Earth and Ocean Sciences, Cardiff University, Cardiff, UK; 4 Research School of Earth Sciences, Australian National University, Canberra, ACT Australia; 5 School of Earth and Environmental Sciences, University of Queensland, Brisbane, QLD Australia; 6 Department of Geology and Geophysics, Yale University, New Haven, CT USA; 7 Department of Earth Sciences, Utrecht University, Utrecht, The Netherlands; 8 Bavarian Geoinstitute, University of Bayreuth, Bayreuth, Germany

**Keywords:** Geochemistry, Geodynamics, Geology, Tectonics

## Abstract

Subduction zones are pivotal for the recycling of Earth’s outer layer into its interior. However, the conditions under which new subduction zones initiate are enigmatic. Here, we constructed a transdisciplinary database featuring detailed analysis of more than a dozen documented subduction zone initiation events from the last hundred million years. Our initial findings reveal that horizontally forced subduction zone initiation is dominant over the last 100 Ma, and that most initiation events are proximal to pre-existing subduction zones. The SZI Database is expandable to facilitate access to the most current understanding of subduction zone initiation as research progresses, providing a community platform that establishes a common language to sharpen discussion across the Earth Science community.

## Abstract (web summary)

Despite numerous advances in our understanding of subduction since the theory of plate tectonics was established, the mechanisms of subduction zone initiation remain highly controversial. Here, the authors present a transdisciplinary and expandable community database of subduction zone initiation events in the last 100 Ma, which establishes a clear direction for future research.

## Introduction

Subduction is the primary driver of plate tectonics on Earth. However, despite numerous advances since the theory of plate tectonics was established, the mechanisms of subduction zone initiation remain highly controversial. While subduction zone initiation (SZI) is particularly important in maintaining plate tectonics, the processes leading to new subduction zones remain poorly understood. This is in part due to the fundamental differences between the dynamics of individual subduction zone initiation events, but also to incomplete or missing and geographically discontinuous geologic evidence, as well as the long timescales and the numerous physical processes involved in forming new convergent plate boundaries.

Owing to the sparse geological record, evidence of both subduction initiation leading to the onset of modern-style ocean-plate tectonics (before 800 Ma), and more geologically recent (<800 Ma) initiation of individual subduction zones, is rare^1–3^. Importantly, SZI is different from early Earth subduction initiation (SI) processes^4^. While early SI dynamics are still debatable (i.e., some sort of subduction may have existed since the very beginning of the planet’s evolution), it is much more certain that the initiation of new subduction zones has actually occurred several times during the last hundred million years^5–7^. Reconstructing the exact location and timing of SZI events, however, remains challenging.

The progression from no subduction to subduction, marked by a slab reaching around 100 km depth, is mysterious: it leaves almost no process-specific geologic traces and it is not clear whether the few indicators (e.g., surface-topographic gradients, new magmatism and/or crustal thickening) observed in some places are applicable to other SZI events^8^. In some cases, new subduction zones might even form without any obvious accompanying magmatism (e.g., ref. ^9^). The onset of a subduction zone is a strongly time and space dependent physical process occurring in a variety of tectonic settings, possibly a function of continental arrangements^10^. SZI is also geodynamically diverse due to a complex interplay between internal and external plate forces, plate structure, rheology, and buoyancy. Moreover, SZI occurs while subduction is occurring elsewhere on the planet, and hence, is possibly influenced by other subduction zones. Newly formed subduction zones are therefore sometimes difficult to distinguish from pre-existing ones, and the sources and directions of the forces triggering them are often unclear. Furthermore, a universal, unambiguous definition describing the fundamental characteristics of subduction zone formation is lacking, and it is unclear which events qualify as SZI, and which do not.

To account for different types of SZI, and their respective dominant forcing, two endmembers have previously been proposed; induced, when horizontal (i.e., tectonic) forces are dominant, and spontaneous, when SZI is mainly driven by the negative buoyancy of the plate alone^1^. Irrespective of the somewhat misleading term spontaneous, SZI is, of course, a dynamic process and is always forced in one way or another (e.g., ref. ^11^).

To enable the formation of a new subduction zone at a pre-existing heterogeneity, or at an intact-plate portion (i.e., not at a plate boundary or lithosphere-scale fault), there has to be either [1] sufficient external plate forcing, [2] focusing of existing internal plate forcing, or [3] localised structural plate weakening. Specific candidates are thought to be important: the structural heterogeneity of a continental margin^10,12,13^ or a pre-existing transform^14–16^ or oceanic-detachment fault^17^; regional plate loading by either sediment^18^ or an oceanic plateau;^19,20^ the plate’s elastic behaviour^15,18,21,22^; the local plate weakening by either water or melt^23,24^, grain-size reduction^25–28^, shear-heating^22,29,30^, or actual void generation;^31,32^ the forcing induced by either small-scale convection in the sub-lithospheric mantle^33^, a delamination^21^, large-scale mantle downwelling^34^, or upwelling mantle plumes^35–38^. The general consensus is that most of these mechanisms are important, but likely require the interplay of others to result in SZI. Despite many potential SZI drivers, it is not yet clear which mechanisms are most common, and whether vertically or horizontally forced (see Box 1) SZI is predominant.

The unresolved questions surrounding SZI include:

- Is the plate or the mantle-flow system more important?
- Do weak spots in the plate system or external forces control SZI?
- Is SZI more likely to occur in regions of compression or extension?
- Does SZI occur nearby plate boundaries or distal from them? Or at passive margins?
- Which sort of plate boundary is the most likely place for SZI to occur?
- If distal from plate boundaries, what are the distances and critical controls?
- Is SZI supported or even induced by pre-existing, nearby subduction zones?
- Is the nature of the upper-plate critical?
- Do subduction zones initiate as a point source or trench-wide?
- Are continent-wide SZI events tied to global plate reorganisations?
- Is SZI restricted to certain regions relative to the global mantle-convection pattern?

To understand SZI, a four-dimensional (three-dimensional space and time) view must be established to overcome the often ingrained (horizontal or vertical) two-dimensional view of SZI. We need to give attention to the regional tectonic setting and plate-mantle dynamics surrounding SZI location. Moreover, it needs to be made clear whether a subduction zone that laterally propagates (e.g., ref. ^39^), or subdivides, or flips in polarity (e.g., refs. ^40,41^), qualifies as SZI event, or not.

**Fig. 1:**
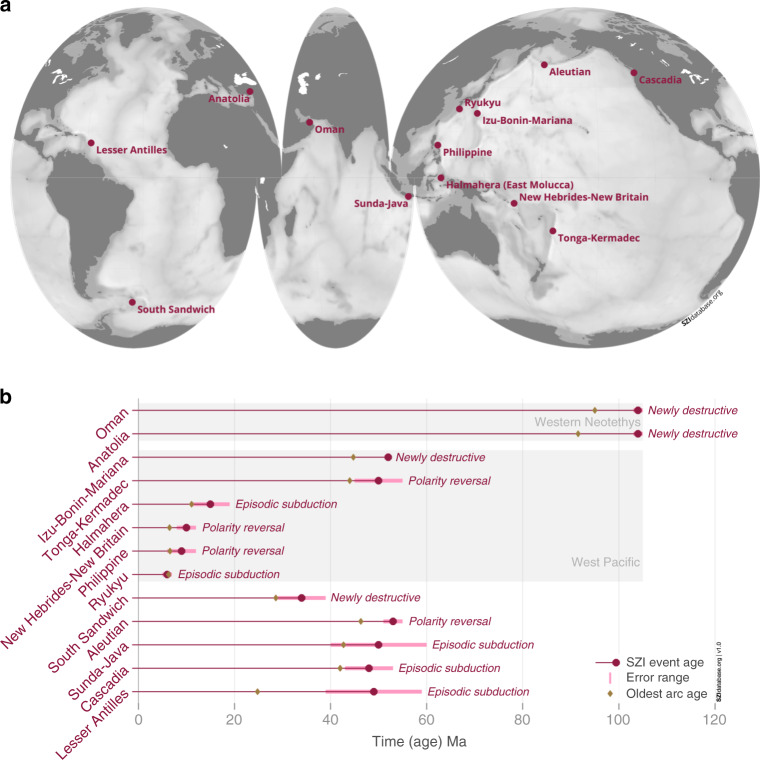
SZI events in space and time. Some of the SZI events that occurred in the last 120 million years are included in the SZI database version 1.0 and are indicated **a** in terms of the present-day geographic locations of corresponding geologic evidence on an interrupted Mollweide projection of the Earth’s bathymetry, and **b** in terms of their temporal occurrence, accompanied by an event-specific uncertainty estimate, oldest known volcanic arc age, and reconstructed SZI type.

**Fig. 2:**
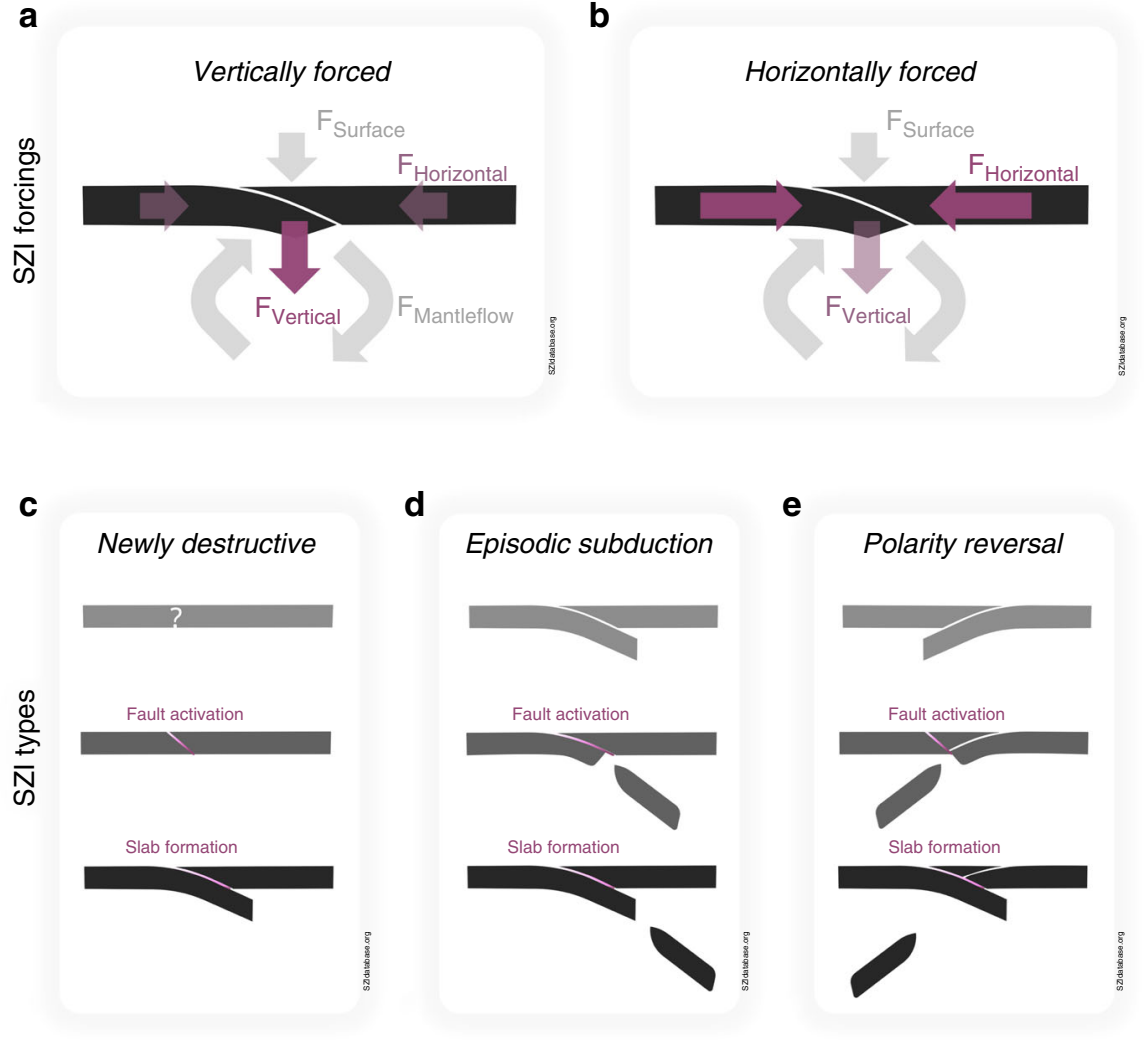
SZI forcing endmembers and SZI types. The two endmembers characterising SZI indicate the dominant forcing to be either—but never exclusively—**a** vertical (i.e., some combination of plate buoyancy force, the force from any surface load, and vertical mantle-flow force), or **b** horizontal (i.e., some combination of tectonic force and horizontal mantle-flow force). All known SZI events can further be grouped into one of the three types, **c** Newly destructive (a subduction fault establishing from an intact-plate portion or some sort of non-subduction-related plate weakness), **d** Episodic subduction (a subduction fault establishing at the same location following a previous, yet terminated subduction zone with the same polarity), and **e** Polarity reversal (formation of a new subduction fault with opposite polarity to the fault of the pre-existing, terminating subduction zone).

The construction of a common, interdisciplinary database for SZI events is therefore instrumental to move forward. The first database version presented here (version 1.0^42^), featuring more than a dozen actual SZI events that occurred during the last hundred million years (see Fig. 1), provides an improved understanding of how subduction zones form on the Earth. We find that during the last 100 Ma, destructive plate boundary formation is predominantly horizontally forced (see definition in Fig. 2a) and that pre-existing subduction zones foster the formation of new ones in their vicinity. The SZI database facilitates easy access to relevant data and the current knowledge of SZI, and establishes a common language together with a fully transparent, expandable platform to sharpen the discussion across the Earth Science communities.

Box 1 **Key terms and definitions**

To perform an interdisciplinary comparison of different subduction zone initiation (SZI) events, it is essential to provide universal, unambiguous definitions for key terms. The definitions provided here should be used throughout the community to minimise communication barriers.

- Subduction zone initiation (SZI)—the onset of downward plate motion forming a new slab, which later evolves into a self-sustaining subduction zone. As such, this definition crucially prevents misjudging simple subduction propagation, expansion, separation, or the failed development of a subduction zone, as a SZI event.
  SZI forcing endmembers:
- Vertically forced SZI—is SZI driven by a dominant, vertical net force arising from a combination of the plate-buoyancy force, a potential force from some surface load, and the vertical mantle-flow force (Fig. 2a).
- Horizontally forced SZI—is SZI driven by a dominant, horizontal net force arising from a combination of the tectonic force and the horizontal mantle-flow force (Fig. 2b).
  SZI types:
- Newly destructive—the formation of a new subduction zone without direct interaction with a pre-existing subduction zone that might occur within a fully intact-plate portion or at a pre-existing plate boundary and/or weakness such as a transform or spreading boundary, or a passive margin (Fig. 2c).
- Episodic subduction—the re-formation of a new subduction zone at the same location and with the same subduction polarity as a pre-existing but terminated subduction zone (Fig. 2d).
- Polarity reversal—the formation of a new subduction zone with opposite subduction polarity facing a pre-existing, dynamically coupled, and terminating subduction zone (Fig. 2e).
  Other essential SZI-related terms (illustrated in Supplementary Fig. 2).
- Early basalts (a.k.a. Forearc basalts (FAB) or Lower lavas)—MORB-like volcanic rocks that are likely products of the very first lavas that erupt during SZI. They underlie Boninites and arc-like volcanic rocks. These basalts differ from typical MORBs due to low Ti/*V* ratios (high *V* at similar Ti contents). They do not exhibit signatures of slab fluids and may be precursors to Boninites/arc-like volcanic rocks. Even though this type of basalt was initially observed in the forearc setting of the Izu-Bonin Mariana system (hence the term Forearc basalt^44^), it is not clear where it is actually emplaced during SZI. The clearer term Early basalts avoids misconceptions about their emplacement.
- Boninites—primitive andesitic extrusive rocks with a chemical composition of >54 wt% SiO_2_, <0.5 wt% TiO_2_ and >8 wt% MgO (Mg# > 0.6). High MgO contents and the presence of clinopyroxene suggest high temperatures and water contents in the mantle wedge with a highly depleted harzburgite residue. Boninites can be subdivided into low-Si and high-Si suites^47^. Whereas low-Si Boninites are not only associated with SZI, the high-Si ones appear to be uniquely associated with SZI. If associated with SZI, Boninites are the first melt products in which the influence of the slab is geochemically observed.
- SZI ophiolites—are remnants of oceanic crust that formed via spreading of the overriding plate during early stages of subduction. As ophiolites, these pieces of oceanic crust and mantle have been tectonically emplaced above sea level (e.g., ref. ^94^), however, SZI ophiolites are distinct from back-arc or mid-ocean ridge ophiolites, which have a different geochemical signature. SZI ophiolites are also referred to as Supra-subduction-zone ophiolites in the literature.
- Metamorphic soles—thin (<500 m thick), fault-bounded sheets of highly deformed meta-volcanic and meta-sedimentary rocks showing an inverted pressure- and temperature gradient that structurally underlie many SZI-ophiolite complexes. Metamorphic soles are interpreted to be derived from the top of a nascent oceanic slab that accreted to the base of the still hot overriding plate during the incipient stages of intra-oceanic thrusting^95–98^.

### Pros and pitfalls of the different approaches

The different Earth science disciplines and their various methods offer valuable information about SZI in general and shine light on individual SZI events on the Earth. Key evidence toward recognising SZI, thus, comes from a wide array of sources, but broadly includes field observations (e.g., structural geology), analytical results (e.g., geochemistry, geochronology and petrology), geophysical data (e.g., seismology), constraints from paleogeographic reconstructions, and the findings of comparative (both numerical and analogue) modelling.

The geologic record might be the most obvious starting point to study SZI. Metamorphic soles and supra-subduction zone ophiolites provide valuable information about the occurrence, timing, and duration of ancient SZI events. Metamorphic soles in particular record the very first stages of the SZI process; therefore, their ages are the best constraint on the age of the very beginning of subduction^43^. Unique volcanic rock types, such as Boninites and Early basalts (found in the arc and forearc of active subduction zones and in ophiolites associated with older events), give an age constraint on a juvenile subduction zone. However, the time delay between SZI and the first appearance of volcanism is uncertain and is likely to vary among different SZI events. To recover a geologic record of recent SZI, ocean exploration has proven very valuable; the most complete geologic evidence so far was extracted by diving and drilling into the Izu-Bonin-Mariana (IBM) plate margin^44,45^. This is also why most current knowledge about SZI events from the perspective of direct geologic observation is based on this one single subduction zone, and is therefore likely biased by this specific SZI event. Other SZI events have been studied in varying detail and provide useful insight into the differences between events, but the terminology used to describe the different studies is often inconsistent. For example, specific terms like Forearc basalt (FAB) (here renamed to Early basalt; see Box 1) were introduced only recently^44^ and have not been considered in previous studies. Furthermore, volcanic rocks like Boninites are not uniquely associated with SZI events as they are also found in volcanic arcs with continuous, ongoing subduction (e.g., ref. ^46^). A distinction between low-Si and high-Si Boninites^47^ might help to better constrain their origin, since high-Si Boninites appear to be uniquely the product of SZI-related volcanism. The possibility of a pre-existing volcanic arc in the vicinity of a SZI event also has to be taken into account, which can further complicate attempts to link the observed arc volcanic rocks to a specific SZI event. Moreover, the ages, including analytical uncertainties, determined for a SZI event need to be interpreted with care. Linking the specific isotopic systems and different minerals used in the age dating analysis to the corresponding evolutionary stage of a subduction zone is crucial to resolving the timing and duration of the SZI process. While a suitable starting point, the geologic record of SZI can and should be strengthened by cross-evaluation with plate reconstructions, seismic tomography, and geodynamic modelling.

Plate reconstructions are a useful tool with which information about the paleogeographic and plate kinematic context of SZI can be extracted and studied. Even though it is not possible to directly infer SZI from a given plate reconstruction, quantitative global reconstructions can provide relevant information about regional plate reorganisation events and inter-regional tectonic correlations that may play a key role in SZI. They can also be used to generate consistent paleogeographic and/or kinematic diagnostics to compare different SZI events within the same paleogeographic model, or the same event as reconstructed in alternative models. The specific tectonic history comprising a quantitative paleogeographic model can furthermore be used to generate testable predictions that can be useful to critically evaluate the corresponding SZI events built into it. The major shortcomings of plate reconstructions are the many unspecified uncertainties (plate reconstructions are interpretations and are often built by multiple contributors) and important assumptions (e.g., absolute plate rigidity) associated with any given model, as well as the fact that there are generally multiple alternative models to explain any given SZI event. Furthermore, plate reconstructions are a compromise between various geological and geochronological datasets, and therefore may not be adequate at the local-scale. With respect to contemporary global plate models of the last few hundred million years (e.g., ref. ^48^), the temporal and spatial resolution is relatively low with respect to the scales at which SZI occurs, and the uncertainty of these models generally increases backward in time.

Seismic tomography inverts observations of seismic waves to generate a model of the Earth’s present-day internal seismic wavespeed structure, providing a means with which to image subducted plate portions presently sinking through the mantle. Seismic tomography therefore allows identified slabs to be associated at least with present-day subduction zones, but furthermore aids in distinguishing separate SZI events (including those associated with subduction zones that have since terminated) and their timings. There exists a number of regional and global tomography models with which a given slab can be identified; recent slab catalogues (e.g., refs. ^49,50^) and slab consistency checks like vote maps^51^ have now emerged to aid in this endeavour. Even though it is clear that SZI should not be inferred from seismic tomography in isolation, owing to a host of uncertainties in the imaging process as well as the manner and rate of slab sinking, the position of an imaged slab can nevertheless provide invaluable information about the approximate timing and former surface location of recent SZI events. It must be kept in mind that the specified depth of a given slab identified by seismic tomography depends on many factors, including artificial model assumptions (e.g., the specific conversion from seismic velocities to material properties) and the non-uniform global resolution of the seismic data. Likewise, inferences drawn for the timing and location of past SZI events on the basis of a slab’s position in the mantle can be complicated by the fact that slabs do not sink purely vertically and that their sinking speeds may vary laterally or temporally owing to variations in temperature and viscosity or the behaviour of the slab (e.g., due to slab folding, deflection, and shallow or mid-mantle break-off).

Geodynamic modelling provides additional insight into the dynamics of SZI and is carried out using analogue and numerical models, basic physics and first-order observables^2^. Studying SZI through geodynamic modelling helps to test and link information from the geologic record, seismic tomography, and plate reconstructions. For example, modelling can provide understanding about the timescales, force balances and kinematic scenarios involved in SZI, as well as the role of mantle-convection, pre-existing structures, and ductile weakening mechanisms. However, geodynamic models are often limited by spatial and/or temporal resolution, approximations of real-world physics, non-linearity, non-unique rheological parameters and imperfectly known initial conditions. Moreover, geodynamic models are per definition never real, nor complete representations of the Earth and therefore never verifiable^52^. As such, much effort is necessary to test them thoroughly and make use of this important tool.

### The subduction zone initiation database

With the subduction zone initiation (SZI) database^42^, we aim to provide clear answers to the above key outstanding questions by not only bringing together previously collected data and insights, but also discussions, from across the various disciplines of the Earth Sciences (Supplementary Fig. 1), which are conveniently organised in a single place (www.SZIdatabase.org). With the SZI database and online platform, we aim to clarify and sharpen ongoing research and debates on SZI by establishing unambiguous definitions and concepts (see Box 1 and Fig. 2). Moreover, the flexibility and dynamic nature of an online database affords the possibility to continuously update the assembled data, revise definitions as our community-wide understanding evolves, and to re-shape prevailing concepts and outstanding questions as new insights arise; and importantly, these revisions can be driven by the community as a whole.

Developing and maintaining such a community-wide database are challenging tasks to undertake; it necessitates transdisciplinary expertise and sufficient academic and methodological flexibility to consider such a major geophysical phenomenon from a novel angle. Fourteen dedicated scientists from across different disciplines have provided the start to revisit, restructure, and reorganise the community’s approach to subduction zone initiation. The result is the community-driven SZI database: www.SZIdatabase.org/contribute.

The SZI database version 1.0^42^ covers more than a dozen SZI events that occurred during the past hundred million years (see Fig. 1). Subduction zone initiation events are classified as such only when they meet the necessary conditions (see Box 1) and are evaluated carefully using an interdisciplinary approach (see Supplementary Fig. 1 and Supplementary Notes 1–15). The resulting compilation provides novel insights into how and under what conditions new subduction zones have formed on the recent Earth.

### Predominant SZI settings and forcings in the last 100 Ma

**Fig. 3:**
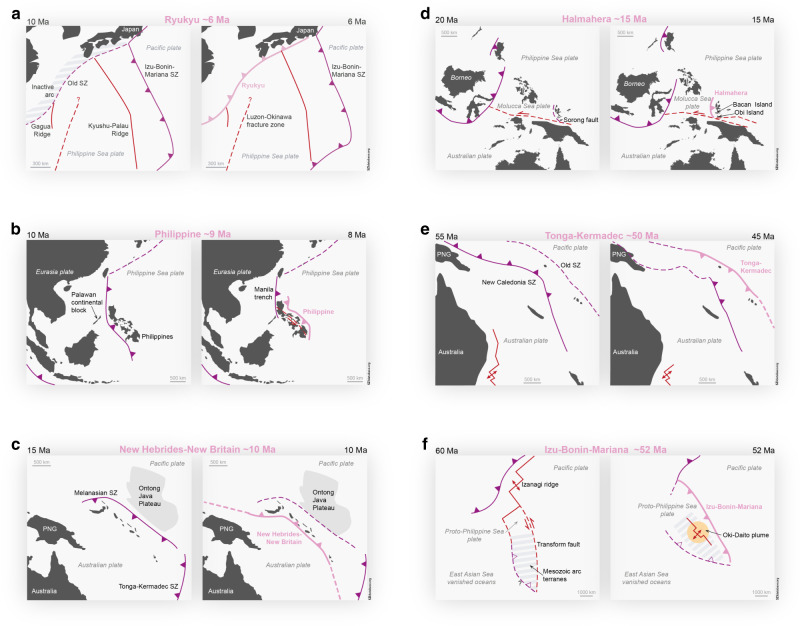
SZI events of the West Pacific subduction realm. The reconstructed events based on the SZI database compilation include **a** the Ryukyu SZI event at around 6 Ma (modified from ref. ^79^), **b** the Philippine SZI event at around 9 Ma^49,80^, **c** the New Hebrides-New Britain event at around 10 Ma^81,82^, **d** the Halmahera SZI event at around 16 Ma^80,83^, **e** the Tonga-Kermadec SZI event at around 48 Ma^84^, and **f** the Izu-Bonin-Mariana SZI event at around 52 Ma^57^. Shown are the new subduction zones (pink lines), other active (solid purple lines) and inactive (dashed purple lines) subduction zones, spreading ridges (solid red lines) and transform faults (red dashed lines).

**Fig. 4:**
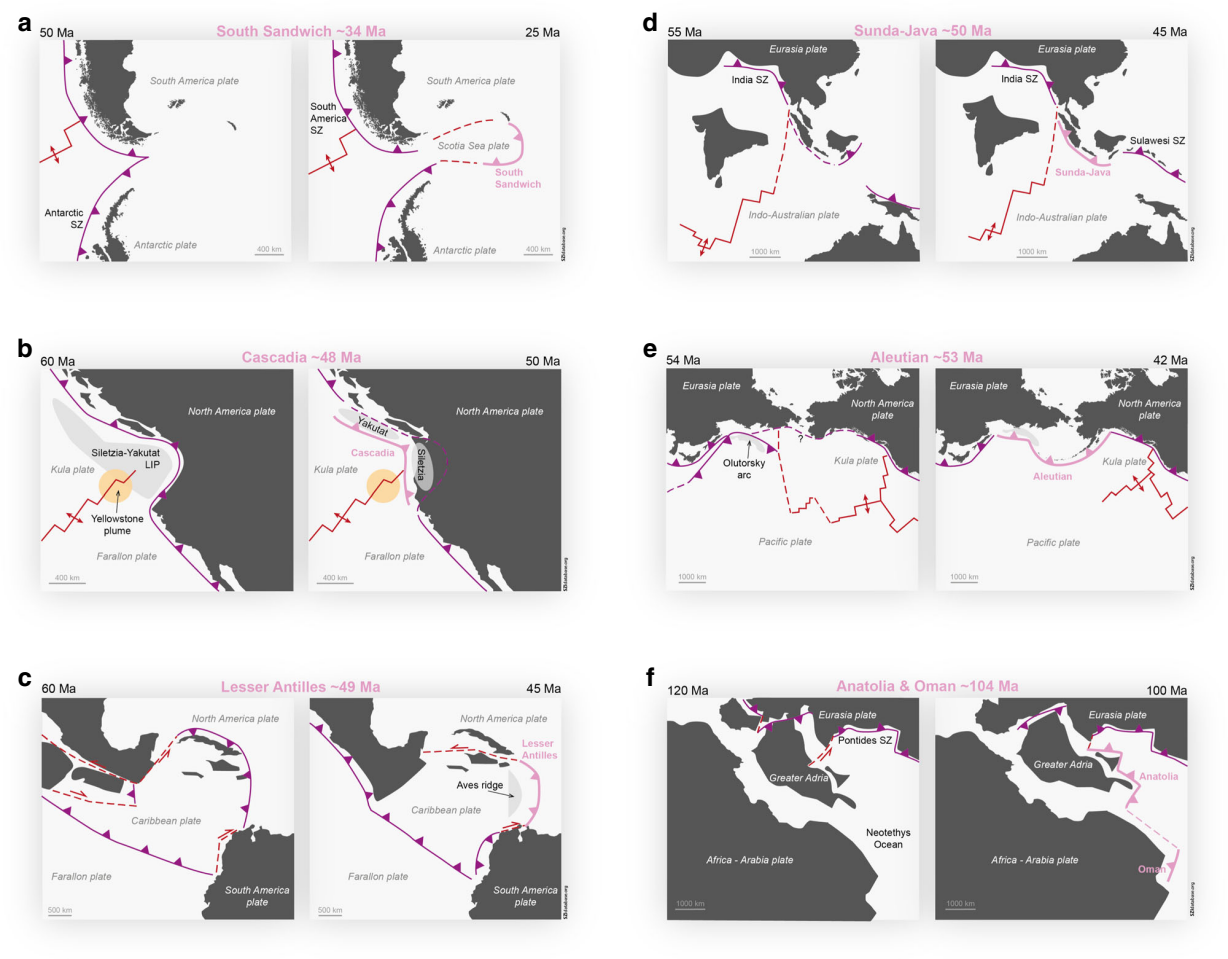
Remaining SZI events included in the SZI database. The reconstructed events based on the SZI database compilation include **a** the South-Sandwich SZI event at around 40 Ma (modified from ref. ^85^), **b** the Cascadia SZI event at around 48 Ma^86,87^, **c** the Lesser Antilles event at around 49 Ma^88,89^, **d** the Sunda-Java SZI event at around 50 Ma^90^, **e** the Aleutian SZI event at around 53 Ma^91^, and **f** the two SZI events, Anatolia and Oman, at around 104 Ma^92,93^. Shown are the new subduction zones (pink lines), other active (solid purple lines) and inactive (dashed purple lines) subduction zones, spreading ridges (solid red lines) and transform faults (red dashed lines).

The compilation and evaluation of the community’s existing data and knowledge as a whole provides new answers to important questions regarding the onset of new subduction zones. Here, we present the main findings of a first database analysis. Figures 3 and 4 display the overall tectonic setting of each SZI event. A more detailed description of each event can be found in Supplementary Notes 1–13.

The SZI database events are spread across the globe, but at least six SZI events occurred in the same region (West Pacific) during the last hundred million years (see Figs. 1a and 3). Moreover, examination of the timing of all SZI events reveals also a temporal clustering in two distinct time periods, between ~55–40 Ma and between ~16–6 Ma (see Fig. 1b), even though the most recent period might be biased by our current choice to not yet classify events as SZI before they have produced self-consistent subduction. Nevertheless, this potential episodic occurrence of multiple new subduction zones could indicate a common underlying SZI driver, like a major plate rearrangement or some perturbation of the mantle system, or, indeed, hint that subduction may foster SZI.

**Fig. 5:**
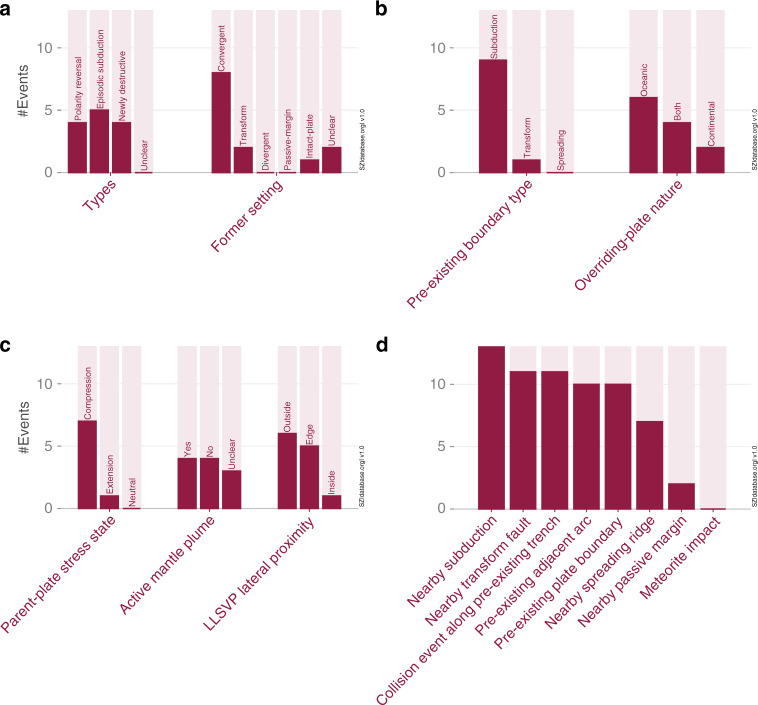
Quantitative SZI database analysis. The number of SZI events that fulfil specific aspects (dark pink bars) versus the total number of SZI events diagnosed (light pink bars; indicating a total of 13 SZI events). **a**–**c** The distribution of all SZI events into the different SZI types and former plate settings, and various other distinctive tectonic and mantle dynamic properties. **d** Total numbers of SZI events representing a given Earth-system characteristic. ‘Nearby’: within a 1500 km radius.

At least nine of all thirteen events identified in the current version of the database occur at or near a pre-existing convergent plate boundary (see Fig. 5a, d). In fact, two of the most common characteristics of all SZI events in the SZI database are initiation along a pre-existing boundary and initiation near a pre-existing subduction zone (see Fig. 5d). This often close association between SZI and pre-existing boundaries (especially subduction zones) is further demonstrated by quantitative investigations of plate reconstructions presented in the ‘Methods’ section.

**Fig. 6:**
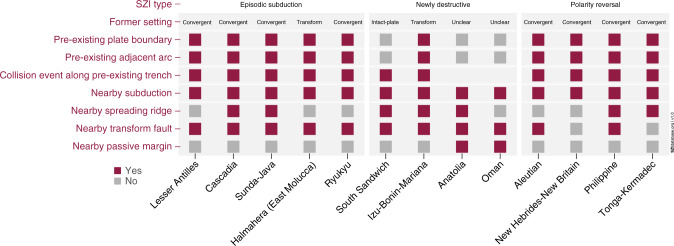
Cross-relations between SZI types and plate structures. SZI events are grouped according to their SZI type and diagnosed for common structural plate features. No square indicates unknown or unclear data.

All SZI events start less than 1500 km away from other active subduction zones and, in most cases, less than 500 km. Transform faults are also often present in the proximity of the SZI event location. Therefore, pre-existing subduction zones and transform faults seem to be the major players for a SZI event or they create favourable conditions for a new subduction zone to develop. Another common feature is the initiation of a new subduction zone close to a pre-existing volcanic arc (see also Fig. 6). We observe that subduction often starts either in the back-arc region of another subduction zone (a case of polarity reversal), or in the forearc region (a case of episodic subduction, wherein an arc is accreted to the overriding plate and subduction jumps to the front), or simply close to older arcs that constitute the overriding plate (as in the case of the Izu-Bonin-Mariana SZI event). This suggests volcanic arcs may comprise a lithospheric weakness or a heterogeneity that is easy to exploit during the formation of a new subduction zone.

Another key observation from our compilation is the many occurrences of collisional events along a pre-existing subduction trench that temporally and spatially coincide with SZI events (Fig. 5d). Such collision events often induce a disruption, by locally slowing down or halting subduction, and thus change the orientation and/or magnitude of the forces acting on a plate, which can foster a SZI event. Notably, we find that subduction zones often initiate through episodic subduction or polarity reversals, even though initiation at newly destructive boundaries occurs too (Fig. 5a). Along the same lines, it is evident that horizontally forced SZI is clearly more common than vertically forced SZI (for definitions of terms see Box 1) in the last 100 Ma.

Active mantle plumes might have fostered some SZI events, but were not a dominant driver for SZI globally since 100 Ma (Fig. 5c). This conclusion is also supported by the fact that SZI events occurred similarly often above the large-low-shear-wave-velocity province (LLSVP) edges (where most mantle plumes of the last few hundred million years appear to have originated^53^), as they did outside the surface-projected areas of the LLSVPs, where mantle-scale downwelling is generally predominant. Indeed, the reconstructed positions of subduction zones over the last ~200 million years match the first-order (global-scale) pattern of faster than average seismic velocities in the underlying mantle, which form a girdle approximately aligned with presently active subduction zones. This suggests that the modern regions of downwelling have been long-lived (e.g., ref. ^54^). Conversely, above the antipodal LLSVP regions, the mantle is largely devoid of seismically imaged-slabs and the surface is absent of reconstructed palaeo-subduction zones. SZI during the last hundred million years has clearly favoured oceanic plate settings (Fig. 5b), and only two SZI events within our database (i.e., Anatolia and Oman) occurred close to (and none directly along) a passive margin (Fig. 5d).

In summary, from the more complete perspective provided by the interdisciplinary SZI database (see Figs. 5 and 6), during the last 100 Ma:

i. subduction zones preferentially form at or near (within 1500 km of) a pre-existing plate weakness (e.g., plate boundary or fracture zone; see also ‘Methods’ section),
ii. SZI events are located outside the surface-projected areas of the LLSVPs, or along their edges.
iii. SZI events occur dominantly within oceanic plate settings in the presence of pre-existing volcanic arcs
iv. collision events along pre-existing subduction trenches are often precursors of SZI events, and
v. subduction breeds subduction.

### Classifying SZI events

Although short on geologic timescales, SZI is by no means an instantaneous process. A SZI event evolves, according to our definition (see Box 1), from lithosphere-scale fault activation to the formation of a subducting plate portion (i.e., slab). Based on relative age measurements on SZI rock sequences and numerical modelling, this process may take up to 5–10 Myr^55^.

Whether in their mature state or early, incipient stages, subduction zones are highly diverse. The only common aspect among all subduction zone initiation (SZI) events is that they are all forced in one way or another. SZI events are either mainly horizontally forced, via external forces that arise, for example, from tectonic or mantle-convection induced stresses, or mainly vertically forced, via a planetary gravitational force acting on density gradients in the plate-mantle system (see Box 1 and Fig. 2a, b).

The apparent SZI diversity can be further grouped into three general SZI types, named newly destructive SZI, episodic subduction SZI, and subduction polarity reversal SZI (see Box 1 and Fig. 2c–e), the latter two of which occur in a pre-existing subduction-zone setting. Such a type-based distinction is important to make, and prevents misjudging simple subduction evolution as SZI events. Subduction propagation, the simple expansion of a pre-existing subduction zone (e.g., lateral widening), and Separation, the splitting up of one pre-existing subduction zone into two or more separate trenches during continuous subduction, cannot be considered SZI as these processes do not form a new subducting slab. Subduction trenches like Yap (southern extension of the IBM system) and Puysegur (south of New Zealand), which are often referred to as having originated from individual, subduction-zone specific SZI events, are not considered as such in the SZI database. Yap cannot be considered as a SZI event since it is, on one hand, not clear if the Yap subduction zone actually formed a self-sustaining subduction zone (and subduction might have already shut down before even reaching that state). On the other hand, it is not clear whether the trench represents a new subduction zone or is a continuation of the Marianas trench (see detailed discussion in Supplementary Note 15). Puysegur is simply too young to tell whether it will develop into a separate, self-sustaining subduction zone and hence qualify as SZI event or not (see Box 1).

### Feasibility of spontaneous SZI

The term spontaneous SZI has commonly been used to describe the initiation of a new subduction zone that is purely driven by the negative plate buoyancy, without forcing external to the plate^1^. In the current version of the SZI database, we find no event that is purely plate-buoyancy driven or, in other words, spontaneous (see e.g., Fig. 5d). In addition, only two (i.e., Anatolia and Oman) of the SZI database events formed nearby a passive margin, which is widely imagined to be the prime location for plate-buoyancy driven SZI. Notably, all analysed SZI events are, in contrast, located in the vicinity of pre-existing subduction systems and the tectonic forces from regional and global plate rearrangements appear omnipresent. The resulting lithospheric stresses easily become large enough to cause SZI (e.g., ref. ^56^), which would indicate a clear predominance of horizontally forced SZI, and likely also vertically forced SZI (that includes vertical mantle-flow forces; see Box 1 for the definition), over spontaneous SZI on the Earth back to at least a 100 Ma.

Moreover, the incessantly convecting mantle provides another omnipresent forcing on each SZI event, which can take the form of both a dominantly vertical (Fig. 2a) or horizontal force (Fig. 2b). Mantle downwelling in particular seems to be dynamically linked to SZI: Most SZI events occur in locations above gross (global-scale) zones of mantle downwelling, such as around the margin of the Pacific Ocean. However, in contrast, some events formed in the vicinity of mantle upwellings; including near mantle plumes and along the surface-projected borders of the large-low-shear-wave-velocity provinces (LLSVPs).

### Future directions

Version 1.0 of the SZI database features most events that occurred in the last 100 Ma. However, more recent SZI events, such as potentially in the Matthew and Hunter area between Matthew Island and Hunter Island, South Pacific, should be suggested by the research community and evaluated to further extend the database.

While there are many SZI events that date back even further, key information about these events typically becomes increasingly sparse with age. Yet, with improving plate reconstructions, progressively more refined geological observations and increasingly more instructive geodynamic models, older events should eventually be included in the SZI database. Conversely, multiple destructive plate boundary formation events might be underway at present-day that cannot be included in the current SZI database because they did not evolve into self-sustaining subduction zones yet (a necessity of SZI per definition; see Box 1). However, it is important to investigate such events as they can provide more detailed information about the process and succession of potential geologic markers of SZI itself, the time period between no subduction and subduction with arc volcanism (e.g., ref. ^8^).

Some events that occurred in the last 100 Ma were deemed too unclear to be included in the current SZI database version (version 1.0^42^). This was either because of the lack of key information or strongly differing, incompatible current interpretations of multiple research groups. Examples of Unclear SZI events are the Manila and the Papua-New Guinea SZI events (see Supplementary Note 14 for a detailed description). Strongly differing reconstructions have been proposed for the onset of the present-day Manila (a.k.a. Luzon) subduction zone^49,57^. In addition, many of the detailed regional reconstructions concerning the history of the Manila subduction zone differ significantly from the default global reconstruction that we have used in the construction of the database^48^, which in turn also differs from some key insights derived from seismic tomography (e.g., ref. ^49^). The Papua-New Guinea SZI is similarly unconstrained; there are at least six competing scenarios for the Cenozoic evolution of New Guinea^58^, owing to an uncertain chronology of major tectonic events prior to the initiation of the presently active trench, as well as poorly constrained subduction polarities. Events like these should be studied in more detail and, if a broad conclusion can be reached, implemented in future versions of the SZI database.

The events that are currently included in the SZI database generally still include a significant amount of uncertainty concerning their timing, location, geodynamic setting, and mechanism. There is little doubt that continued work on each of these events will further clarify these various aspects and provide important updates for future versions of the SZI database. In addition, more data entries, such as the age of high-pressure metamorphism or upper-plate deformation structures, should be suggested by the community and added to the database to characterise the compiled SZI events in more detail.

Future geological expeditions, plate reconstructions and geodynamic modelling studies of SZI should aim to fill the gaps in the database, while acknowledging that some database entries will demonstratively remain empty. As a community, we should similarly concentrate on those SZI events that still offer additional data or can be clarified. For example, further drilling expeditions should be undertaken around the globe to find additional (possibly complete) SZI ophiolite sequences to complement the currently incomparable evidence of the Izu-Bonin-Mariana SZI event^59^. Possible sites of further exploration include the Tonga subduction zone, where the sediment cover is minimal, the New Caledonia subduction zone, a fossil but extensively exposed system, the subduction system spanning Matthew and Hunter Islands in the South Pacific, which may still be an ongoing SZI event^60^, and the Puysegur Trench and Macquarie Ridge, which are interpreted as strike-slip boundaries under oblique compression that appear to be transitioning into a subduction zone. The latter two locations, in particular, may provide a rare insight into ongoing SZI dynamics within a directly observable tectonic framework.

In addition to seeking to fill the gaps, we can also use the current content of the SZI database to help steer and sharpen future research on SZI. For example, the type and timing of different magmatic successions observed from the geologic data assembled from one SZI event can be used to formulate testable hypotheses concerning another (perhaps less well understood or poorly studied) SZI event. Likewise, future plate reconstructions can be refined with reference to the latest data (e.g., high-resolution, multi-geochronometer timing of SZI events) and insights (e.g., SZI appears to generally produce narrow new plate boundary segments, which then continuously widen) provided by the SZI database. In turn, updated and refined plate reconstructions can be used to further critically evaluate the kinematic self-consistency of the prevailing tectonic models of the database, as well as to provide additional testable hypotheses. As subduction polarity reversals and episodic subduction are shown to be the most predominant SZI types in the last 100 Ma, we suggest that future geodynamic modelling studies should focus on these events rather than the less common spontaneous subduction events. Lastly, the insights gained from the database show how important it is to not treat a SZI event as an isolated process. Instead, we must study SZI as part of its much bigger, evolving tectonic and mantle dynamic framework.

The SZI database as presented here (version 1.0) is by no means a complete and perfectly accurate compilation of all SZI data and knowledge. We, therefore, find it important that not only our team, but the whole Geoscience community can contribute to improve and extend the database from here onwards. Contributions can be conveniently submitted via an online form on www.SZIdatabase.org/contribute (see also Section 5.1 Methods). To further facilitate continued progress on a trajectory of open, accessible, and community-driven science, we built a platform that reaches far beyond the common individual research communities, so that everyone can learn about SZI, share a common language, and discuss and exchange new ideas and research on the topic. The online SZI database forum (www.szidatabase.org/forum) is a timely platform and should be used for an ongoing, community-wide discussion.

## Conclusions

We introduce a novel, interdisciplinary subduction zone initiation database to better understand one of the pivotal processes shaping our planet’s unique evolution: the initiation of subduction zones maintaining ocean-plate tectonics. Subduction zone initiation (SZI) during ongoing ocean-plate tectonics is fundamentally different from subduction initiation (SI) that might have kickstarted ocean-plate tectonics on the early Earth. To prevent further confusion with simple subduction propagation or separation, we clearly define the concept of SZI and other relevant terms for improved cross-disciplinary communication (see Box 1).

By combining diverse expertise and data related to geological and geochemical observations, plate reconstructions, seismic tomography, and geodynamic modelling, we collect and interpret a myriad of fragmentary evidence on actual SZI events to build a better understanding of how new subduction zones form. We merge this accumulation of current knowledge of SZI into one common, freely available repository and provide a much-needed transdisciplinary overview of both the fundamental and negligible aspects of SZI. The individual SZI events are (and will remain) too few to draw statistically sound conclusions. Nevertheless, we gain key insights from the current compilation of SZI events that occurred in the last ~100 Ma.

- Purely plate-buoyancy driven (i.e., spontaneous) SZI is unlikely on the present-day Earth and, in the last 100 Ma, only examples of SZI with either significant horizontal forcing, or else an additional vertical mantle-flow forcing, are observed.
- Since 100 Ma, subduction zones form preferentially at or near a pre-existing plate weakness, like a plate boundary or a fracture zone, which indicates the necessity of heterogeneities in plate-strength. However, only two compiled SZI events seems to have occurred close to a passive margin.
- Most SZI events are located outside the surface-projected area of the LLSVPs, or along their edges, suggesting that both mantle upwelling and downwelling could play a significant role in fostering SZI.
- SZI events occur dominantly within oceanic plate settings in the presence of pre-existing volcanic arcs, indicating that heterogeneous oceanic plate interiors are more favourable for SZI than ocean-continent boundaries, or more specifically, passive margins.
- Collision of buoyant features with pre-existing subduction trenches is often a precursor of SZI events as they likely trigger changes in the forces acting on the plates and can foster SZI.
- SZI events occur predominantly in the vicinity of pre-existing subduction zones, indicating that subduction breeds subduction.

For the SZI database and further SZI research, we established a transdisciplinary, universal language with up-to-date, unambiguous definitions and concepts. Together with an interactive online platform (www.szidatabase.org), this will allow for a more effective communication across different, diverging disciplines of the Earth Sciences, and also with the general public, and therefore pave a fresh new way forward in our quest to unravel the remaining secrets of how new subduction zones form on the Earth. We encourage the community to interact, improve and, crucially, extend the database from here on.

## Methods

### Database format and sources

The subduction zone initiation (SZI) database currently covers most of the well-studied SZI events back to about 100 Ma that, with the exception of Anatolia and Oman, formed presently active subduction zones. Each SZI event is described by parameters reflecting relevant data gleaned from the geologic record, seismic tomography, and plate reconstructions. In addition, an interdisciplinary interpretation of each event is provided through some key parameters that are based on objective diagnostics. Apart from the actual value, each parameter is assigned a tag, an uncertainty value, its dimension, its source (i.e., a reference), if applicable its coordinates, and a comment describing it in more detail. The user guide provided within the SZI database package provides general guidelines and additional information regarding each database entry.

The SZI database consists of individual data files, one for each covered event. Each data file is divided into four sections named Interpretation, Direct evidence, Reconstructions, and Seismic tomography.

The Interpretation section provides key interpretations based on a cross-disciplinary, community-wide basis like the time of subduction zone onset (i.e., age), the reconstructed location of the SZI event, and geodynamic information like SZI type and geographical freshness. The Interpretation section also provides informative entries about the regional geodynamics and nearby (i.e., within a 1500 km radius) structural and dynamic features during the time of SZI. The available data for possibly relevant, coinciding global extreme-events, like climate extremes, are also provided. The Interpretation section therefore provides some means to characterise the different SZI events, and relate and compare them to each other. Two indicators in particular (Global subduction system and Geographical freshness; see below for details) provide the events’ affiliation to regional subduction zone systems and their geographical and geometrical relation to co-existing, adjacent plate boundaries. The latter is a sequence of strings that contains condensed information about whether the SZI event occurred at a pre-existing plate discontinuity or not, and the approximate distance and orientation between the newly formed subduction zone and the nearest pre-existing subduction trench.

The Direct evidence section provides the facts relating to the SZI event from observations and direct measurements like the age of ophiolite crystallisation and of metamorphic sole formation/cooling. Details on the arc sequence, from Early basalts to Boninites to arc volcanic rocks, include the oldest and youngest ages found in the literature for these different magmatic expressions. When the presence of Early basalts and/or Boninites is not clearly related to the SZI event, their ages are not considered, but can be found in the comments section.

A myriad of reconstructions has previously been presented for the various SZI events considered here, many of them schematic. In order to present more consistent, quantitative metrics for each SZI event in the context of a common, global, reconstructed plate framework, we have elected to analyse the global full-plate model of Müller et al.^48^ (version 1.14; hereafter M16). The Reconstructions section covers a wide range of relevant geodynamical data extracted (where implemented) from M16 using GPlates (version 2.1; www.gplates.org). As a global model, M16 was generated from numerous regional studies, which are also referred to in the database, and we direct the reader to the corresponding papers for details. We would like to underscore that our usage of this model does not imply that we agree with all the specific kinematic histories presented by it, but rather employ it as a convenient and effective tool with which to make quantitative comparisons of the SZI events as they are presently implemented in it. We note that alternative regional and global reconstructions exist, as well as updates and corrections, including a correction to the kinematics of the Pacific realm before 83 Ma^61^. Data from M16 was extracted both with use of the standard tools available in GPlates, and by Python and MatLab scripts that were constructed to operate directly on the rotation poles, full-plate polygons and age-grids that comprise the M16 model. Examples of the former (GPlates analyses) include a determination of the timing of a SZI event, the nature and identity of the plates involved, and whether the newly initiated subduction zone nucleated along a pre-existing structure. Examples of the latter (custom script analyses) include calculation of the approximate length of the newly initiated subduction zone and the density of other plate boundaries in the vicinity of it, the age of the first subducted lithosphere, and the relative velocity along the juvenile trench.

The Seismic tomography section presents relevant data from observations of the mantle’s present-day seismic wavespeed structure and, more specifically, of structures associated with anomalously fast seismic wavespeeds (i.e., subducted slabs). In particular, the depth of the bottom of slabs connected to the surface can provide key information about the time at which SZI occurred, for example by assuming a constant slab sinking rate^62^. The collection of such data concerning the location of fast seismic wavespeed anomalies in the mantle was undertaken via two independent approaches. The first approach utilised seismic vote maps, following the methodology of Shephard et al.^51^. Vote maps highlight consistent wavespeed features seen across multiple tomography models, and thereby reveal anomalously fast wavespeed structures that are comparatively well-resolved and most likely robust. We constructed vote maps with use of the tomographic data repository and web-based analysis tools provided by SubMachine (www.submachine.earth.ox.uk^63^). We specifically used a combination of five global P-wave tomography models (DETOX-P01^64^; GAP-04^65,66^; HMSL-P06^67^; MITP-2011^68^; UUP07^69^) and five global S-wave tomography models (S40RTS^70^; Savani^71^; HMSL S06^67^; SEMUCB-WM1^72^; TX2015^73^). Using these vote maps, we estimated the depths of the bottoms of subducted slabs inferred to be related to the SZI events considered in the database. The determination of slab tops and bottoms was done based on the associated pixel depth of at least 7 (of maximum 10) votes.

Our second approach was to exploit an existing online database (Atlas of the Underworld; https://www.atlas-of-the-underworld.org/) of positive wavespeed anomalies for which the depths of slab tops and bottoms have already been estimated, and an interpreted association of slabs to specific subduction zones has already been provided^50^. The tomographic data underlying the Atlas of the Underworld comes from one P-wave tomography model (UUP07^69^) and one S-wave model (S40RTS^70^). This atlas includes some slabs identified by other regional studies, and we refer the reader to the corresponding papers listed there for further details.

### Geographic freshness classification

All SZI events are classified using a generally applicable geographical and structural freshness classifier (see Supplementary Fig. 3). The classification is based on a cross-disciplinary interpretation of the SZI event and provides crucial information in a condensed string about the presence of a pre-existing lithospheric weakness, the closeness of pre-existing subduction zones and their geometric relation to the SZI event location. It consists of three indicators. The first is either [A], representing SZI at an intact-plate portion, or [B], SZI at pre-existing plate discontinuity (i.e., plate boundary or lithosphere-scale fault). This is followed by one of the numbers [3000, 2000, 1000, 500, 250, or 100], which represents the upper limit of the horizontal distance (i.e., offset) between the SZI event location and the nearest pre-existing subduction trench. This is followed by either [C], which denotes for a SZI event laterally offset along strike or in continuation of the closest, pre-existing subduction trench, or [P], which denotes a SZI event with a normal offset, but otherwise parallel to the closest, pre-existing subduction trench, or [O], which denotes for a SZI event with a normal offset, but oblique position to the closest, pre-existing subduction trench. Combining the three individual classifiers leads, using the Izu-Bonin-Mariana SZI event as an example, to a complete string in the form of B100O, indicating SZI less than 100 kilometres away from, and oblique to, a pre-existing convergent plate boundary.

### Quantitative reconstruction analysis

To further quantitatively explore possibly important connections between SZI and a variety of parameters implicitly defined by the reference plate reconstruction used here^48^, we have developed a set of simple tools to compute the following metrics from each SZI event implemented in the reference plate model:

1. The length of the SZI trench (notably, this does not necessarily imply that the SZI event was associated with instantaneous subduction along the full length of the boundary, but is rather a convenient measure of the approximate length of the system shortly after its inception);
2. The total length of each kind of pre-existing tectonic boundary (convergent, divergent, transcurrent) within a variety of radii of the boundary defined by (1);
3. The percent length of each pre-existing boundary type defined in (2), relative to the SZI trench length defined in (1);
4. The minimum, maximum and mean age of the subducting oceanic lithosphere at SZI time. This is computed with reference to the oceanic age grids provided by Müller et al.^48^, and achieved by sampling all oceanic lithospheric ages from the down-going plate, within a nominally small distance of the SZI trench as defined in (1).

The results of these computations are provided in Supplementary Data 1 and on the SZI website (www.szidatabase.org/resources).

Supplementary Data 1 contains a collection of raw data, methodological settings and analyses concerning parameters that have been extracted from the global topological (full-plate) tectonic model of Müller et al.^48^, the default plate model used in the construction of SZI database version 1.0. For clarification, we use the GPlates-compatible files of Müller et al.^48^, specifically model version 1.14, together with their age-grid model version 1.11.

The structure of the Supplementary Data 1 spreadsheet is broken into two parts: the first are the Müller et al.^48^ extracted data and their collection methods, organised by SZI event, comprising nine individual sheets (Aleutian, Lesser-Antilles, Halmahera, Izu-Bonin, New Hebrides-New Britain, Philippine, San Cristobal-New Hebrides, South Sandwich, Sunda-Java, and Tonga-Kermadec). The second part is the Analysis sheet, wherein data from all the events are compiled and considered in different combinations.

Each ‘Data and methods’ sheet is further broken into two parts: a boundary analysis, and an age-grid analysis. Each boundary and age-grid analysis lists a corresponding event file (both analyses share a common event file for a given SZI event) that comprises a list of vertices defining the SZI trench at initiation time, according to the model of Müller et al.^48^. We have somewhat arbitrarily taken the full length of the polyline defined by Müller et al.^48^ as the initial length of the SZI trench; in many cases, this is likely not representative of the true initial zone of failure, and future work could be dedicated to constructing more detailed representations of the initial failure zones of these SZI events. Nevertheless, these polylines provide a convenient, common quantitative reference, and can effectively serve as an upper bound for the initial SZI trench lengths. These polylines can be found on www.SZIdatabase.org/resources. We then reconstruct these polylines according to the rotation scheme of Müller et al.^48^, and extract data with respect to them either at the SZI time, or immediately (1 Ma) before.

For the boundary analysis, we determine the total lengths and types of other plate boundaries nearby the SZI trench, where the term nearby is defined by some variable great circle distance (GCD) threshold from the vertices comprising the SZI trench. Our setup for this exercise involves a pre-conditioning step wherein all the constituent polylines comprising the topological polygon file associated with the model of Müller et al.^48^ are seeded with additional nodes such that there is a minimum spacing of 0.1° between each node. For each event and reconstruction time considered, we then find the total lengths of (1) other subduction zones and (2) ridges and transforms that lie within a specified GCD threshold of any node along the SZI trench polyline; we specifically considered three thresholds of 2.25° (~250 km), 4.5° (~500 km), and 9.0° (~1000 km). In addition to measuring the total distance of each plate boundary type, we also report these values scaled to the total length of the SZI trench.

The age-grid analysis is conducted by using the reconstructed SZI trenches to identify locations along the subducting plate, immediately adjacent to the trench, from which to sample age data from the age-grids provided by Müller et al.^48^. To accomplish this first requires identifying the plate IDs of the subducting plates, and then constructing a global reference mesh (achieved by querying velocity domain nodes in GPlates using a 256 × 256 Terra mesh) from which nodes close to the SZI trench (we used a GCD threshold of 0.5°) can be identified. We then use a nearest neighbour algorithm to query the age-grids of Müller et al.^48^ at the closest nodes to these reference points. To consider the role of the age-grid resolution, we have performed this on grids with a resolution of both 0.5° and 0.2°. From the resulting age population, we then determine the minimum, maximum and mean age of the subducting plate at SZI time.

The analysis sheet shows a collection of comparisons between the data collected and presented in the data and methods sheets. These include comparisons between subducting plate age (SPA) and the various SZI parameters (age of SZI, trench length, length of other subduction zones).

### Current SZI events in the database

The current SZI database version 1.0 covers thirteen actual SZI events that occurred on the Earth since around 105 Ma and are sufficiently well constrained (see Table 1). These SZI events (and their age estimate) are listed, from oldest to youngest, below.

- *Anatolia* (104 ± 1 Ma)
- Oman (104 ± 1 Ma)
- Aleutian (53 ± 2 Ma)
- Izu-Bonin-Mariana (52 ± 0.5 Ma)
- Sunda-Java (50 ± 10 Ma)
- Lesser Antilles (49 ± 10 Ma)
- Tonga-Kermadec (50 ± 5 Ma)
- Cascadia (48 ± 5 Ma)
- South Sandwich (34 ± 5 Ma)
- Halmahera (15 ± 4 Ma)
- New Hebrides-New Britain (10 ± 2 Ma)
- Philippine (9 ± 3 Ma)
- Ryukyu (6 ± 1 Ma).

**Table 1:** The SZI events included in the SZI database version 1.0.

Event name	Subduction system	Age (Ma)	Type	Former setting	Geographic freshness
Lesser Antilles	Atlantic	49 ± 10	Episodic subduction	Convergent	B250P
Cascadia	East Pacific	48 ± 5	Episodic subduction	Convergent	A250P
Sunda-Java	Indian Ocean	50 ± 10	Episodic subduction	Convergent	B250P
Aleutian	North Pacific	53 ± 2	Polarity reversal	Convergent	A250P
South Sandwich	South Atlantic	34 ± 5	Newly destructive	Intact-plate	A1000P
Ryukyu	West Pacific	6 ± 1	Episodic subduction	Convergent	B100O
Philippine	West Pacific	9 ± 3	Polarity reversal	Convergent	A250P
New Hebrides-New Britain	West Pacific	10 ± 2	Polarity reversal	Convergent	A250P
Halmahera (East Molucca)	West Pacific	15 ± 4	Episodic subduction	Transform	A2000P
Tonga-Kermadec	West Pacific	50 ± 5	Polarity reversal	Convergent	B500P
Izu-Bonin-Mariana	West Pacific	52 ± 0.5	Newly destructive	Transform	B100O
Anatolia	Western Neotethys	104 ± 1	Newly destructive	Unclear	A500P
Oman	Western Neotethys	104 ± 1	Newly destructive	Unclear	B2000O

Details about each individual event and its interpretation can be found in Supplementary Notes 1–13. The Geographic freshness classifier is explained in Supplementary Fig. 3.

Other events were classified as unclear (e.g., Manila; Papua-New Guinea), or as a non-SZI event (e.g., Yap; Puysegur), or still need to be considered (e.g., Matthew and Hunter and other potential candidates; see, for example, refs. ^8,60^). More detailed summaries for all included SZI events are provided on SZIdatabase.org and in Supplementary Notes 1–15, including relevant Supplementary References.

### Database availability

The SZI database is made openly available to the scientific community and the general public. All relevant SZI data can be accessed and downloaded via the webpage www.szidatabase.org, where we also provide high-resolution graphics, including dark versions of the manuscript figures. Versioning and long-term availability of citable sources is enabled by a Zenodo repository^42^.

SZIdatabase.org provides a simple user interface to quickly and freely access not only the database files as a whole, but also key SZI data. In addition, a variety of figures and graphs summarising the latest insights and present-day knowledge are made available online. A glossary with key SZI terms is provided that aims to prevent confusion and facilitate communication across individual scientific communities but also with the general public. Key existing software that is used to build the SZI database is linked on the webpage alongside custom-made software to visualise individual database variables and events.

### Database extensibility

SZIdatabase.org provides a convenient means to suggest changes and additions to the existing database via a submission form (www.SZIdatabase.org/contribute). Suggestions for additions and improvements will be reviewed by our interdisciplinary group and built into future versions of the SZI database on a regular (e.g., yearly) basis. In addition, the SZI Database Forum (www.szidatabase.org/forum) is a convenient platform for the whole community to openly evaluate SZI database versions, suggest additions and improvements, and, importantly, discuss and exchange new ideas and recent results about the onset of new subduction zones.

## Supplementary information

Supplementary Information

Description of Additional Supplementary Files

Supplementary Data 1

## Data Availability

The SZI Database is available on www.SZIdatabase.org and reposited as Crameri et al.^42^.

## Code availability

The open-access SZI Database plotting tool for MatLab to visualise specific SZI parameters directly from the SZI database sheets can be found within the database package, or separately on www.SZIdatabase.org/resources^74–78^.
