# Supplementary material for: A transdisciplinary and community-driven database to unravel subduction zone initiation
Source: Nat Commun. 2020 Jul 27;11:3750. doi: 10.1038/s41467-020-17522-9 (PMC7385650; doi:10.1038/s41467-020-17522-9)
Supplement: Supplementary file 1 — Supplementary Information [file 41467_2020_17522_MOESM1_ESM.pdf]

Supplementary Information for "A transdisciplinary and community-driven database to unravel subduction zone initiation"

Fabio Crameri[1], Valentina Magni[1], Mathew Domeier[1], Grace Shephard[1], Kiran Chotalia[2], George Cooper[3], Caroline M. Eakin[4], Antoniette Greta Grima[2], Derya Gürer[5], Ágnes Király[1], Elvira Mulyukova[6], Kalijn Peters[7], Boris Robert[1], Marcel Thielmann[8]

[1] *Centre for Earth Dynamics and Evolution (CEED), University of Oslo, Postbox 1028 Blindern, 0315 Oslo, Norway*

[2] *Department of Earth Sciences, University College London, Gower Street, London WC1E 6BT, United Kingdom*

[3] *School of Earth and Ocean Sciences, Cardiff University, Cardiff, United Kingdom*

[4] *Research School of Earth Sciences, Australian National University, Canberra, Australia*

[5] *School of Earth and Environmental Sciences, University of Queensland, Brisbane, Australia*

[6] *Department of Geology and Geophysics, Yale University, New Haven, USA*

[7] *Department of Earth Sciences, Utrecht University, Utrecht, Netherlands*

[8] *Bavarian Geoinstitute, University of Bayreuth, Bayreuth, Germany*

Contents

Supplementary Figures 3

Supplementary Note 1: Aleutian 6

Supplementary Note 2: Anatolia 9

Supplementary Note 3: Cascadia 11

Supplementary Note 4: Halmahera 13

Supplementary Note 5: Izu-Bonin-Mariana 17

Supplementary Note 6: Lesser Antilles 20

Supplementary Note 7: New Hebrides-New Britain 23

Supplementary Note 8: Oman 27

Supplementary Note 9: Philippine 29

Supplementary Note 10: Ryukyu 32

Supplementary Note 11: South Sandwich 34

Supplementary Note 12: Sunda-Java 38

Supplementary Note 13: Tonga-Kermadec 42

Supplementary Note 14: Unclear SZI events 45

Supplementary Note 15: Non-SZI events 46

Supplementary References 47

Supplementary Figures

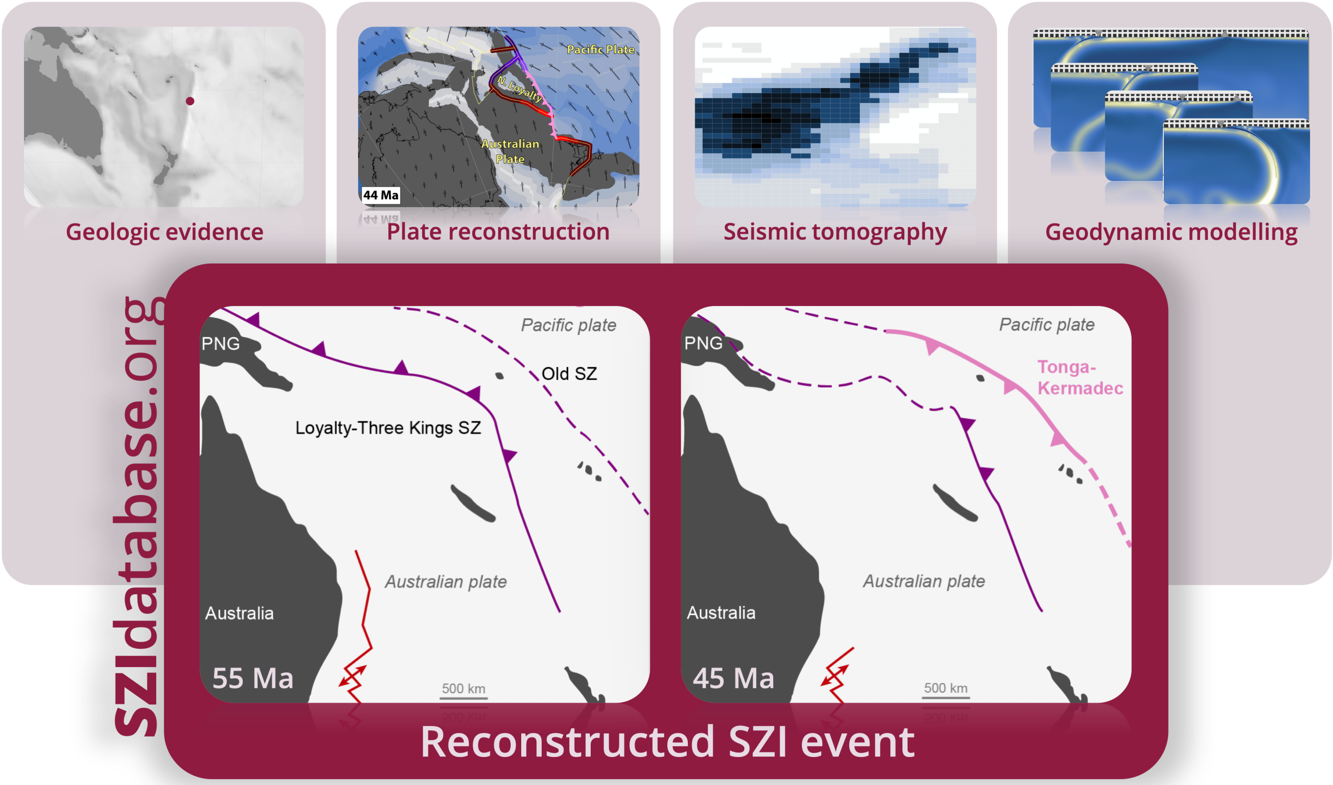

Supplementary Figure 1 SZI database fundament. The underlying procedure of the SZI database combines direct geologic evidence with plate reconstruction, seismic tomography, and geodynamic modelling provided by the various scientific communities to infer individual SZI events, which are eventually provided back to the scientific communities via the interactive online platform www.SZIdatabase.org.

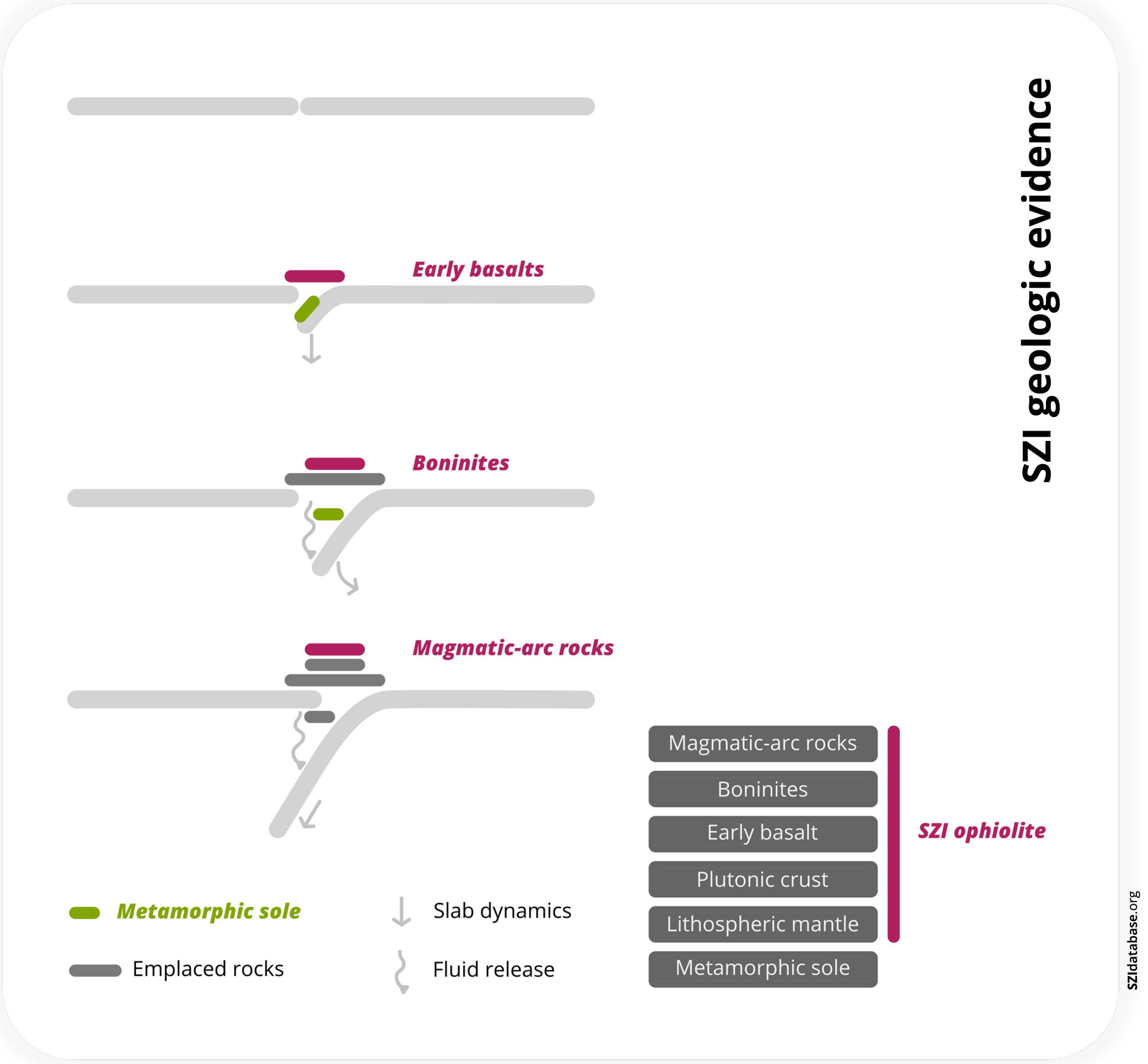

Supplementary Figure 2 SZI geologic evidence. Schematic illustration of the emplacement of SZI-typical rocks during SZI, and a typical SZI ophiolite sequence. Note that this is a text-book example (according to e.g., the *Izu-Bonin-Mariana* SZI) and that pre-existing structures (e.g., a pre-existing volcanic arc) or variable SZI dynamics (e.g., horizontal compression) could inhibit various stages and therefore their typical rock signatures.

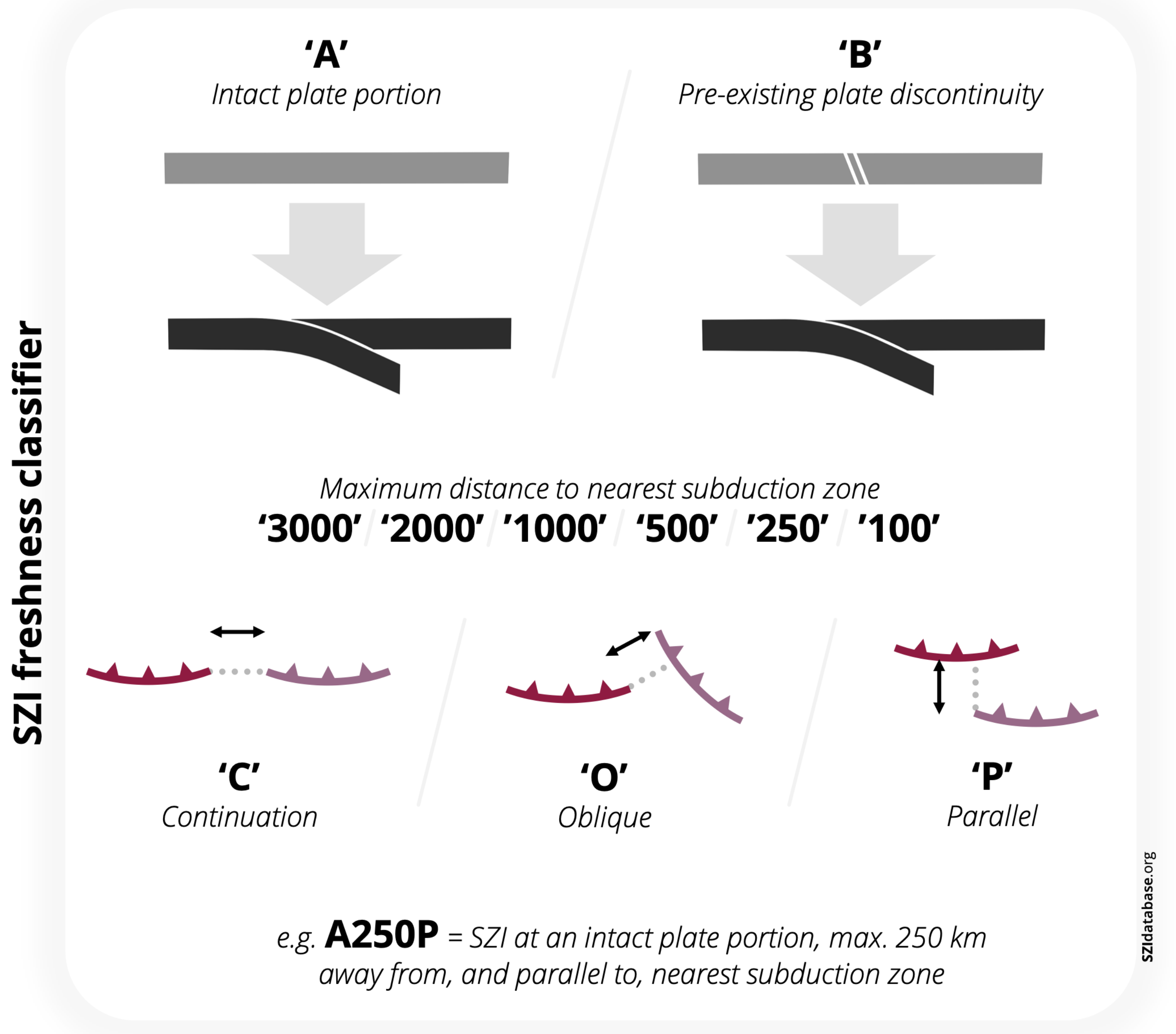

Supplementary Figure 3 SZI freshness classifier. The objective classification provides crucial information in a condensed string, consisting of three indicators, about the presence of a pre-existing lithospheric weakness, the closeness of pre-existing plate boundaries and their geometric relation to the SZI event location.

Supplementary Note 1: Aleutian

Interpretation. The Aleutian subduction zone initiation event formed today's active Aleutian trench (Supplementary Figure 4). The onset of the subduction zone likely occurred at around 53 Ma[1,2] when the Pacific and Kula plates began to subduct northward, and at some point, under the overriding continental plates of northeast Siberia and North America.

The Aleutian SZI event was possibly instigated as a subduction polarity reversal associated with the arrival of an intraoceanic arc (Olutorsky arc) to the Okhotsk-Chukotka-Beringian margin of northeastern Asia and northwestern North America[3,4,5], and therefore may have formed close to (i.e., at a distance of around 300 km) a pre-existing convergent plate boundary. Vaes et al.[5] have speculated that the Aleutian trench may have exploited a pre-existing transform boundary in a possible backarc behind the Olutorsky arc. Those authors have furthermore pointed out that arc volcanics with ages of 54.4 Ma to 50.2 Ma have been dredged from the Beringian margin[1], and note that the Aleutian SZI event could also be seen as the outboard jump of that Beringian subduction zone.

Direct evidence. The oldest rocks in the Aleutian arc are andesites dredged from Murray Canyon[2] and primitive basaltic rocks from Medny Island, both of which are 46 Ma[6].

Reconstruction. In the model of Müller et al.[7] (see Supplementary Figure 5), the Aleutian SZI event occurs at 55 Ma, by the outboard (southward) jump of a pre-existing Kamchatka-Alaska subduction zone; this jump ranges from less than 100 km in the east to ~500 km in the west. The Olutorsky arc is not implemented in this model (nor is any other intra-oceanic arc in this region at this time), but instead a regional plate reorganisation event is temporally paired to subduction of the Izanagi ridge. This plate reorganisation occurs at 55 Ma, and includes both a re-direction of the Pacific plate (from NW-directed to N-directed) and an acceleration of the Kula plate's northward drift.

Seismic tomography. Seismic tomography (see Supplementary Figure 6) reveals a slab attached to the trench and reaching a maximum depth of around 700 km[8].

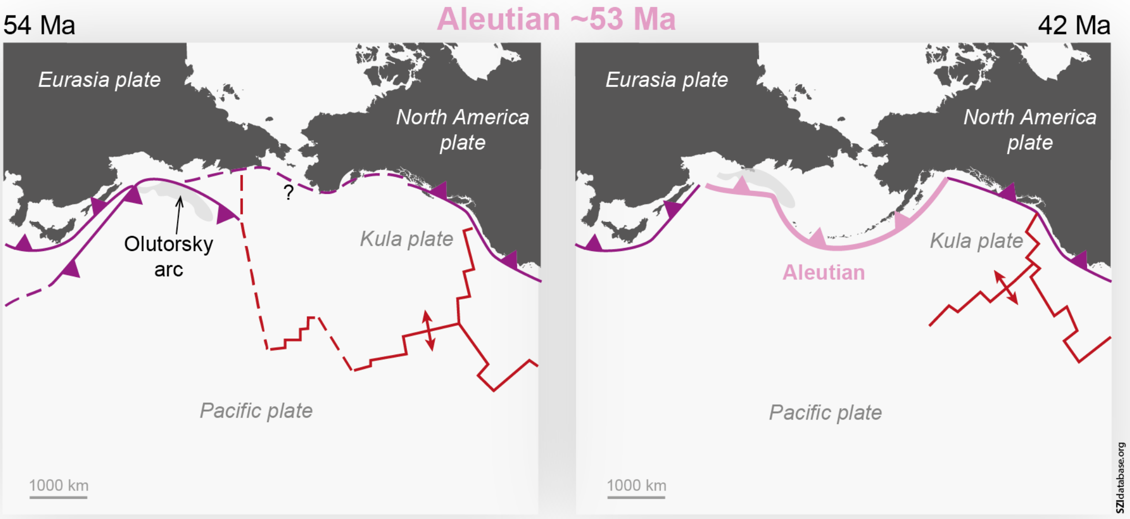

Supplementary Figure 4 Schematic tectonic reconstruction of the Aleutian SZI event modified from ref.[4]. The collision of the Olutorsky arc with the trench of the south-dipping subduction of the Eurasia plate below the Pacific Plate is suggested to have caused a flip in subduction polarity, initiating the new Aleutian subduction zone. Shown are the new subduction zone (pink line), other active (solid purple lines) and inactive (dashed purple lines) subduction zones, spreading ridges (solid red lines) and transform faults (red dashed lines).

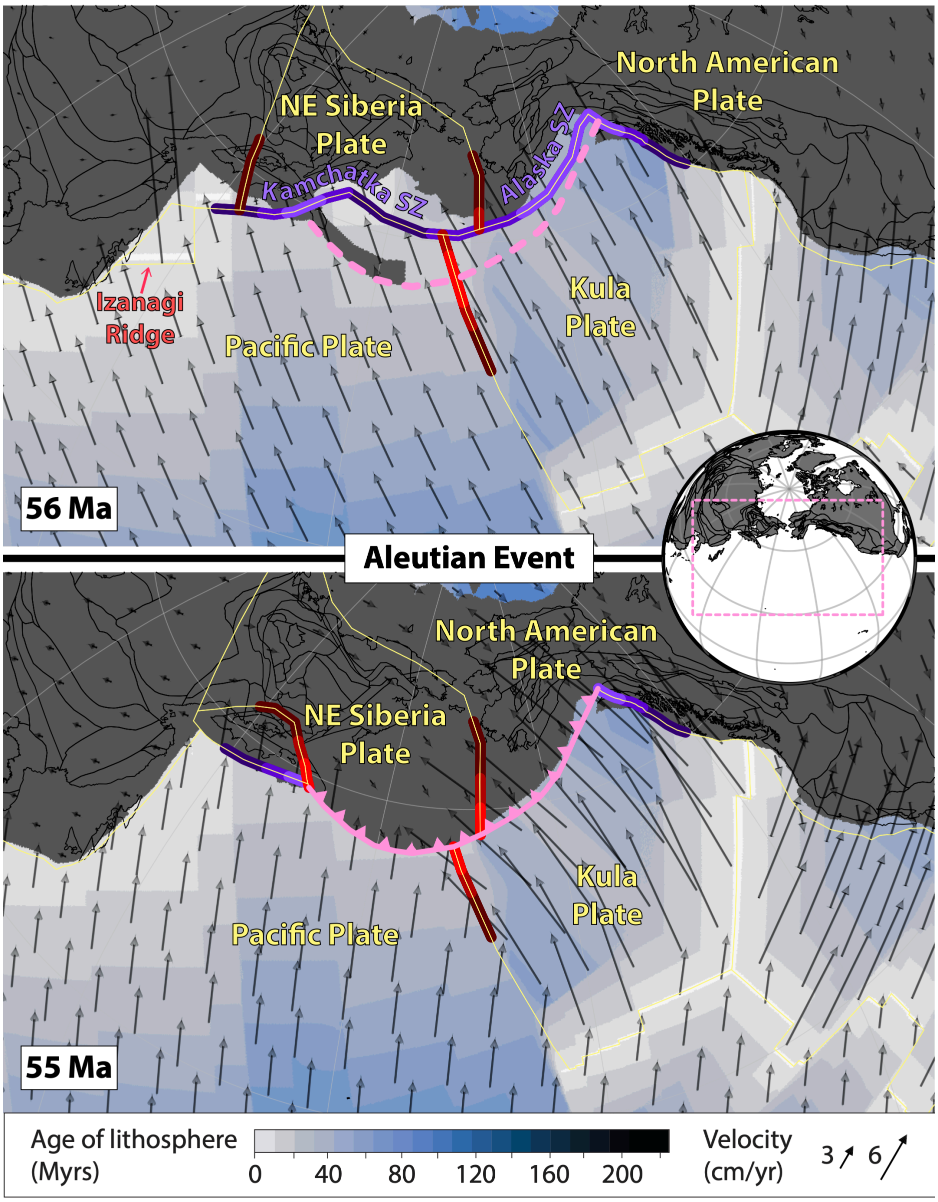

Supplementary Figure 5 Aleutian SZI event as reconstructed in the model of Müller et al.[7]. Pink dashed (solid with teeth) line shows the Aleutian trench 1 Myr before (at) SZI time in the model. Purple (red) lines show segments of neighbouring subduction zones (ridges and transforms) that lie within some radius of the Aleutian trench (pink line); the brightness of the colours reflects 3 different distance thresholds of 250, 500 and 1000 km.

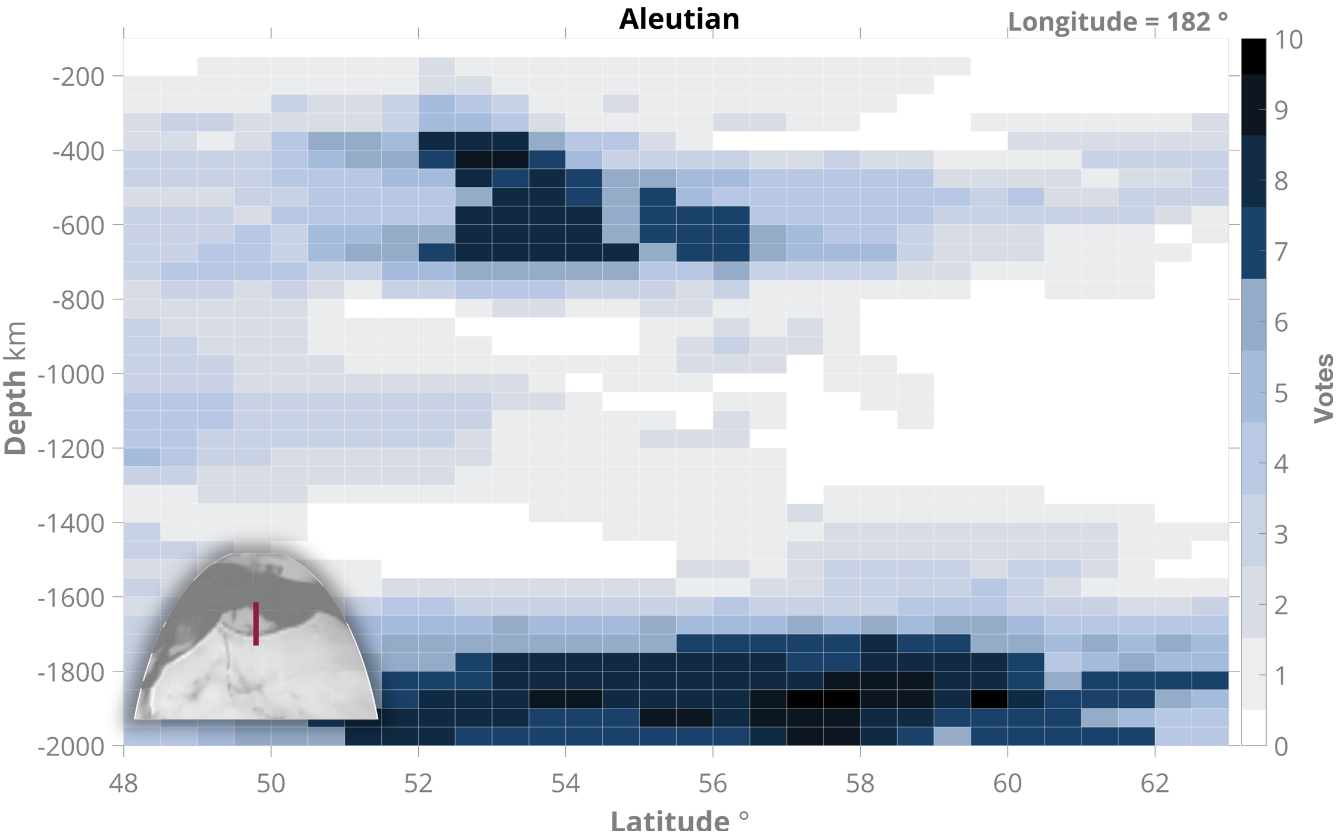

Supplementary Figure 6 Seismic tomography VoteMap[9] analysis of the Aleutian SZI event.

Supplementary Note 2: Anatolia

Interpretation. The Anatolia subduction zone (also referred to as 'Anatolian Neotethys' or 'southern strand of the Neotethys') started at around 104 Ma (Supplementary Figure 7). It initiated within the oceanic lithosphere of the Neotethys and, together with the Oman SZI event, represented the western Neotethyan subduction system. SZI has been proposed to be the consequence of fracture zone inversion[10], though this remains a matter of debate[10,11].

At the time of SZI, both downgoing and overriding plates were oceanic lithosphere of the Neotethys; the overriding oceanic lithosphere has also been termed 'Anadolu plate'[12]. The final stages of subduction of the downgoing plate saw the arrival and accretion of continental lithosphere (the Africa-Arabia plate), including several microcontinents of 'Greater Adria'[13,14].

Direct evidence. Presently, the only remnant of this subduction system is represented by the Cyprus trench[15,16]. The collision along strike of the paleo-trench resulted in widespread ophiolite obduction. The timing of subduction initiation at ~104 Ma (based on Lu-Hf) is inferred from prograde garnet growth in metamorphic soles underlying Anatolian ophiolites[17,18]. The time lag between pro-grade metamorphism and the crystallisation of supra-subduction forearc crust, based on zircon U-Pb ages (94–90 Ma; e.g. van Hinsbergen et al.[19], and references therein), argue for a horizontally forced SZI event contemporaneous and in the northwestward continuation of Oman[10,20]. The oldest arc age from the Uckapili granite in the Central Anatolian Crystalline Complex (or Kirsehir arc) is ~91 Ma[21,22].

Reconstruction. The model of Müller et al.[7] does not implement this SZI event; the northern margin of 'Greater Adria' in this model is passive at ~104 Ma, lying in the middle of the 'Dinaride plate', which was subducting to the north beneath Eurasia at this time. The northern margin of 'Greater Adria' remained passive until ~80 Ma, when north-dipping subduction locally appeared to the south of the Kirsehir Block (between the Kirsehir Block and the Menderes-Taurides Block), and elsewhere the margin remained passive until its arrival to the southern active margin of Eurasia.

Seismic tomography. In the Atlas of the Underworld[8], Anatolian subduction is related to both the Antalya and Cyprus slabs (which are difficult to tomographically distinguish from each other). In the vote maps, a slab is identified between 250–1550 km depth (see Supplementary Figure 8).

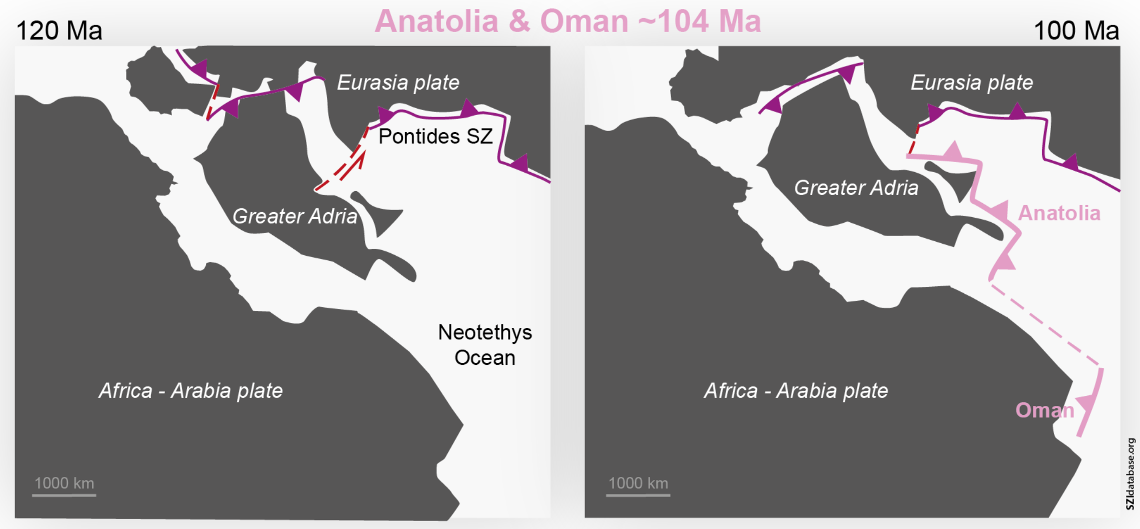

Supplementary Figure 7 Schematic tectonic reconstruction of the Anatolia SZI event modified from ref.[10]. Shown are the new subduction zone (pink line), other active subduction zones (solid purple lines) and transform faults (red dashed lines).

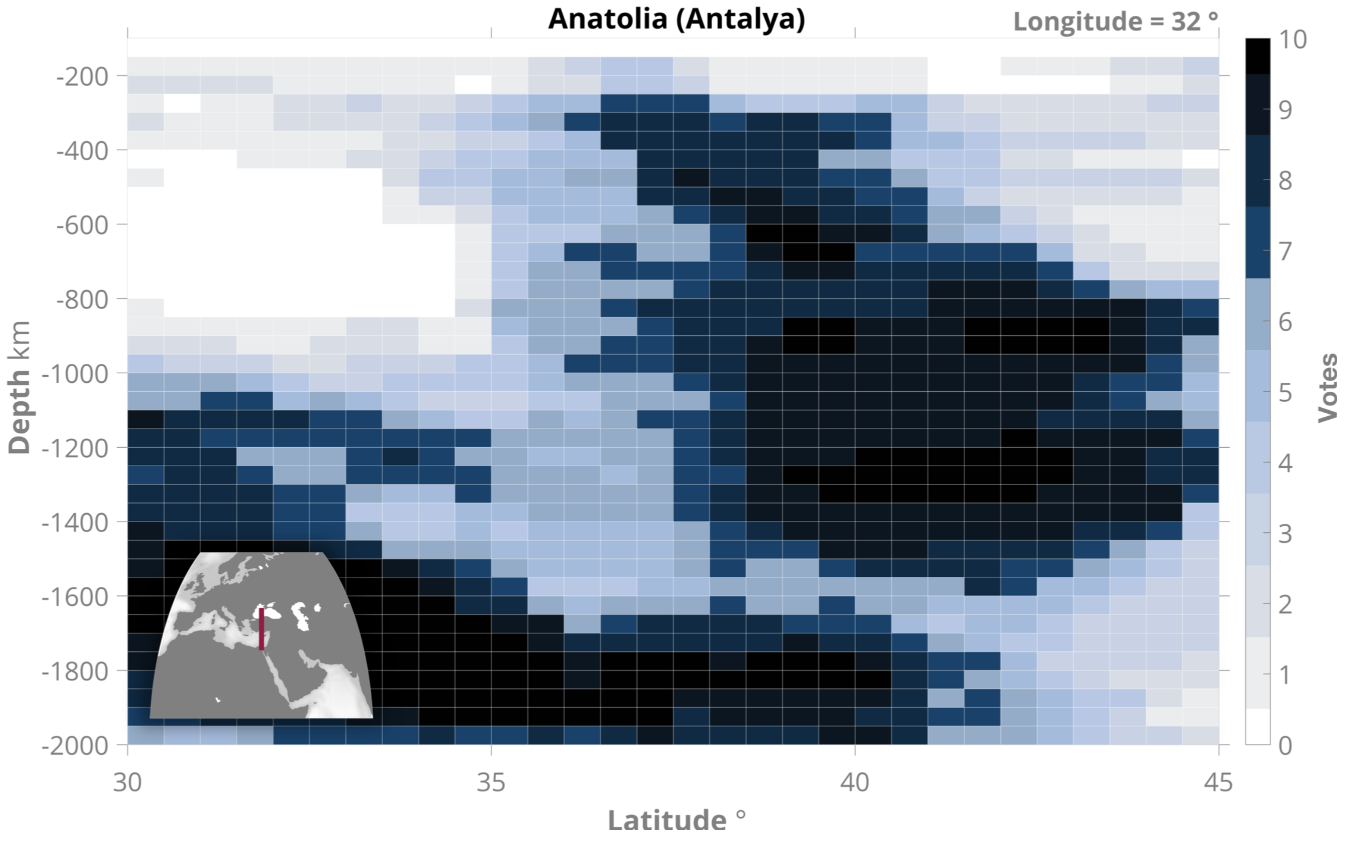

Supplementary Figure 8 Seismic tomography VoteMap[9] analysis of the Anatolia SZI event.

Supplementary Note 3: Cascadia

Interpretation. The Cascadia SZI event formed the currently active Cascadia subduction zone at around 53–43 Ma[23,24,25,26] and induced subduction of the Farallon and Kula plates below the North America plate (Supplementary Figure 9).

The Cascadia SZI event is believed to have occurred as an episodic subduction via a trench jump after the large igneous province (LIP) Siletzia accreted at the previous trench of the Farallon/Cordilleran subduction zone[26,27]. There is also an indication of the presence of the prominent Yellowstone mantle plume during the time of the onset, which might have been facilitated by the breaking of the intact subducting plate[26]. It is worth noting that at the time of the trench jump there was ongoing subduction to the north and south of the Cascadia subduction zone. Although the accretion of the Siletzia block is likely to be the main cause of the trench jump, the nearby subduction zones in the north and south might therefore also have had a role on this SZI event.

Direct evidence. Siletzia volcanic rocks on northwestern Washington (Olympic Peninsula) were emplaced between 53.18 ± 0.17 Ma and 48.364 ± 0.036 Ma, based on zircon U-Pb ages[28]. Deformation associated with Siletzia emplacement has been suggested to be between 51.309 ± 0.024 and 49.933 ± 0.059 Ma, as inferred from shortening in the forearc basin[28]. The oldest ages of the Cascadia arc are found mostly in south-west Washington in basalts and basaltic andesites, and have age ranges of 45–36 Ma[29]. Wells et al.[27] document a sill complex phase related to subduction initiation to 48–45 Ma.

Reconstruction. The model of Müller et al.[7] does not implement this SZI event, and east-dipping subduction along the western margin of North America remains uninterrupted in this model during this time interval.

Seismic tomography. P-wave seismic tomography images a slab attached to the surface at the Cascadia subduction zone that reaches 350 km depth[8]. The anomaly is broken into two parts, separated by the Yellowstone plume. S- and P-wave vote map tomography images agree on a small fast anomaly between 200–250 km depth (see Supplementary Figure 10).

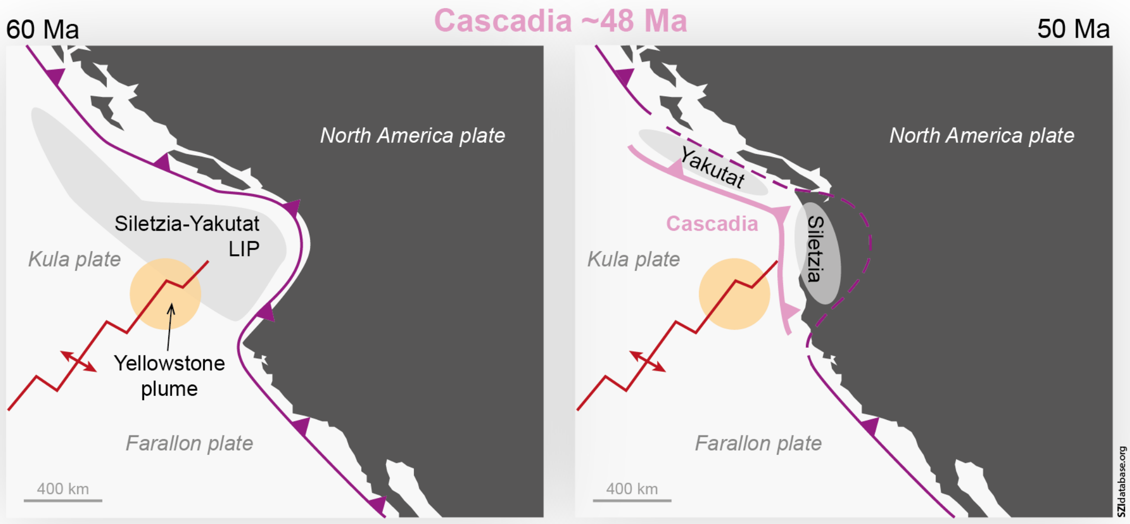

Supplementary Figure 9 Schematic tectonic reconstruction of the Cascadia SZI event modified from refs.[26,27]. A trench jump occurred due to the accretion of the Siletzia and Yakutat large igneous province (LIP) formed by the Yellowstone plume, initiating the new Cascadia subduction zone. Shown are the new subduction zone (pink line), other active (solid purple lines) and inactive (dashed purple lines) subduction zones, and spreading ridges (solid red lines).

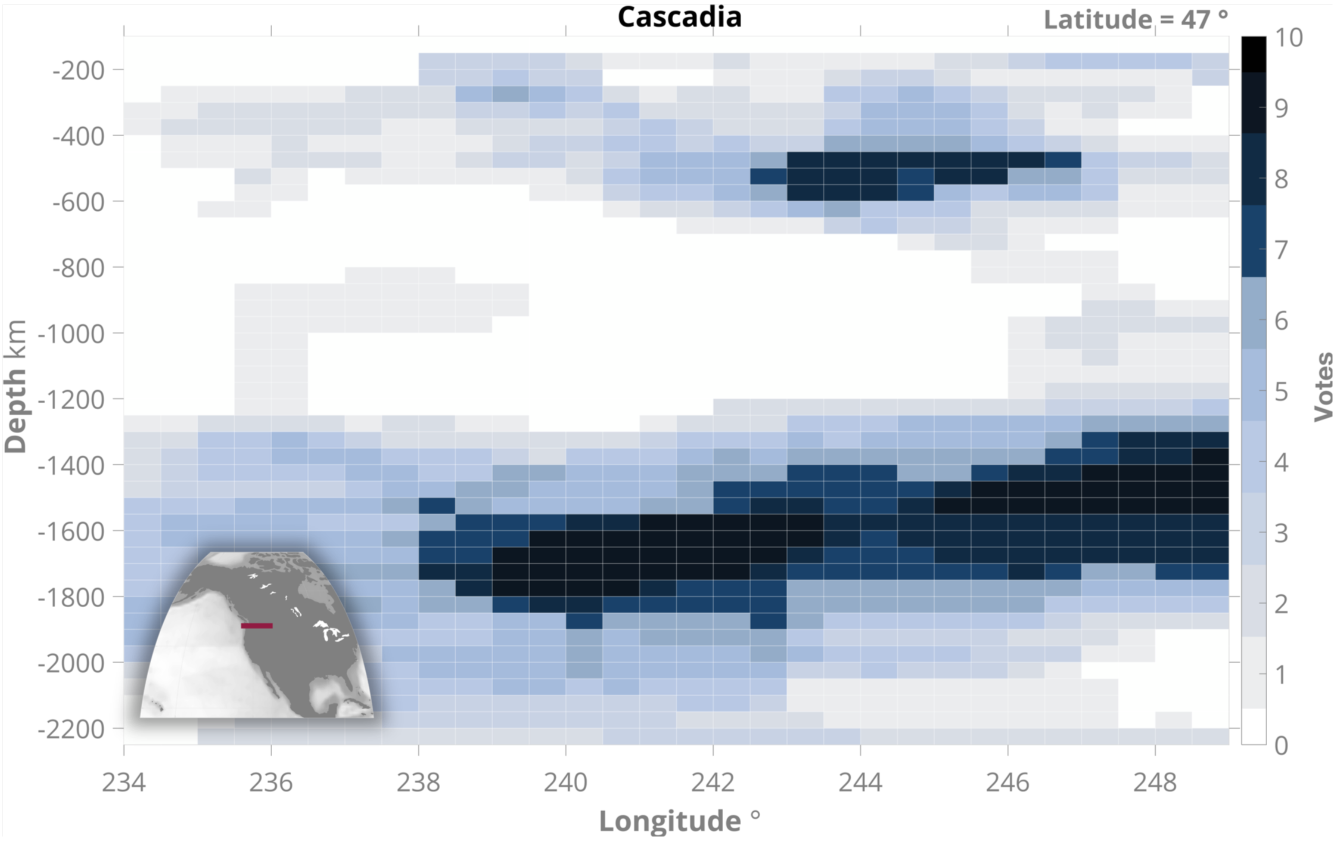

Supplementary Figure 10 Seismic tomography VoteMap[9] analysis of the Cascadia SZI event.

Supplementary Note 4: Halmahera

Interpretation. The Halmahera SZI event (Supplementary Figure 11) − associated with the subduction of the Molucca Sea plate eastwards beneath the Philippine Sea plate − was previously estimated to have initiated at around 17–15 Ma[30,31], but more recent studies have alternatively suggested a younger age of ~10–7 Ma for SZI[32,33,34]. Given the evidence presented below, SZI must be older than 11 Ma. Considering a ~5 Myr delay in line with[30] we suggest ~15 Ma as our best-estimate for Halmahera SZI, but emphasise that this preference is likely to be controversial and may be revised downward if additional evidence demonstrates a disconnect between the oldest arc volcanism (on Obi Island) and later subduction beneath Halmahera. The type of SZI is also unclear, but could be ascribed to episodic subduction, as there is evidence of subduction along Halmahera from the Mesozoic to the Oligocene. That subduction ceased by the time of a regional plate boundary reorganisation at about 25 Ma, whereafter subduction initiated on the western side of the Molucca Sea plate, along the Sangihe arc[35]. Hall and Smyth[35] suggested that Halmahera SZI was caused by locking of the Sorong fault zone along the southern edge of the Molucca Sea. Considering the broader geodynamic picture, Halmahera SZI occurred perhaps within ~20° of the Manus plume[36], and approximately above the edge of the Pacific LLSVP (i.e., along the edge of its surface projection, according to its present-day shape).

Direct evidence. The older (17–15 Ma) age estimate for SZI is based on the presence of ~11 Ma calc-alkaline volcanic rocks on Obi Island at the southern end of the Halmahera system, which are recognised as the oldest arc-related Neogene volcanic products of the system[30]. However, Hall and Spakman[32] have suggested that the Obi Island volcanic rocks may be due to "to minor subduction within the northern New Guinea strike-slip zone" (a strike-slip bounding the Halmahera system to the south; also called Sorong fault zone), and thus potentially unrelated to SZI of the main Halmahera subduction zone. They further point out that arc-related volcanism on the island of Halmahera does not appear until 8–7 Ma, and therefore argue for a younger (~10 Ma) initiation of the Halmahera subduction zone. While acknowledging that the Obi Island volcanic rocks may be in part due to strike-slip processes, we note that if "minor subduction" began along Obi Island and later propagated northward, our definition of SZI would still recognise that earliest phase of subduction to be the SZI event itself. We further note that Baker and Malaihollo[30] report the occurrence of 9.5–8.8 Ma acidic volcanic rocks from Pulau Bisa (north of Obi Island), which seem to reinforce the notion of northward propagating arc activity, and the occurrence of ~15 and ~9 Ma diorites on Bacan Island (between Obi and Halmahera islands). The latter intrusive rocks could be arc-related, although no coeval volcanic counterparts have been recognised.

SZI along the Sorong fault zone could have produced the calc-alkaline volcanic rocks found on Obi Island and then subduction (and arc magmatism) may have propagated northward to Pulau Bisa, Bacan and then Halmahera islands by ~10–7 Ma[30]. Notably, the Neogene Halmahera subduction zone succeeded an earlier Mesozoic to Eocene subduction zone along Halmahera[35,37], and so the younger subduction zone may have exploited a pre-existing structure.

Reconstruction. In the model of Müller et al.[7], the Halmahera SZI event occurs at 14 Ma, in conjunction with the Philippine SZI event (the subduction zones are contiguous but of opposite polarity) (see Supplementary Figure 12). Halmahera SZI immediately follows the cessation of a pre-existing subduction zone (the 'East Philippine' subduction zone) within ~250 km of the Halmahera trench; to the east the trace of these two subduction zones become progressively closer until they merge. Coincident with the SZI event (at 14 Ma), the motion of the Philippine Sea plate changes significantly, as does the neighbouring Caroline plate to the immediate east of the Halmahera trench, whereas the motion of the Australian plate remains unchanged.

Seismic tomography. Subducted slabs of the Molucca Sea plate are well-imaged by seismic tomography, and resemble an inverted "U-shape" due to the occurrence of both west-dipping subduction beneath the Sangihe arc and east-dipping subduction beneath Halmahera. However, in the case of the seismic anomalies beneath Halmahera, it is not straightforward to differentiate the slab of (younger) Neogene Halmahera subduction from lower-mantle anomalies that likely represent the remnants of earlier (Mesozoic-Eocene) subduction (see Supplementary Figure 13). van der Meer et al.[8] interpret the Halmahera slab to reach ~760 km depth, whereas other interpretations consider the anomalies below 440 km to be separated from the Halmahera slab and associated with older subduction[34]. The former interpretation is more consistent with an older (~17–15 Ma) timing for SZI, whereas the latter is more consistent with a younger (~10–7 Ma) initiation.

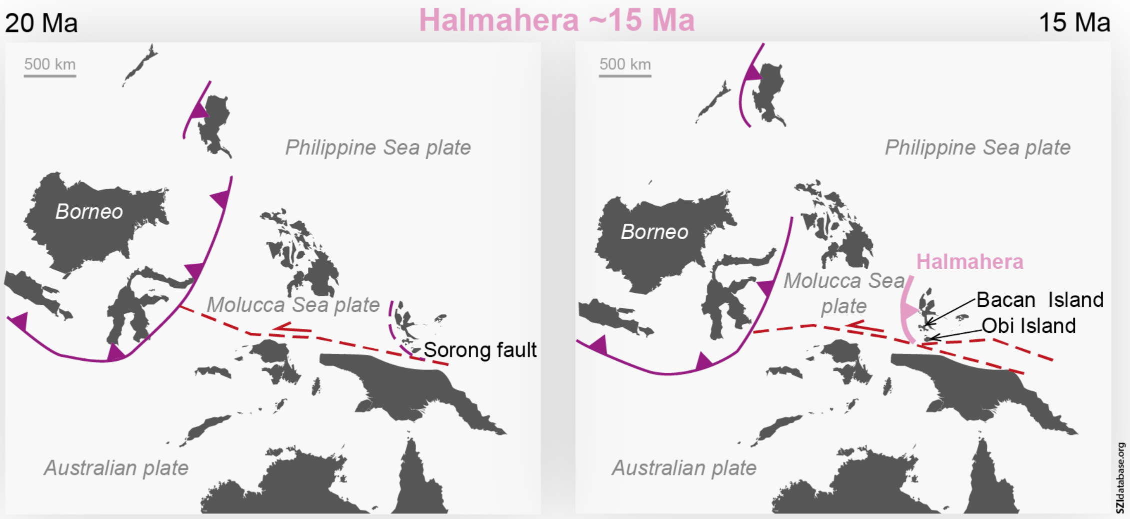

Supplementary Figure 11 Schematic tectonic reconstruction of the Halmahera SZI event modified from ref.[31]. Subduction of the Molucca Sea plate started close to a transform boundary, initiating the new Halmahera subduction zone. Shown are the new subduction zone (pink line), other active subduction zones (solid purple lines), and transform faults (red dashed lines).

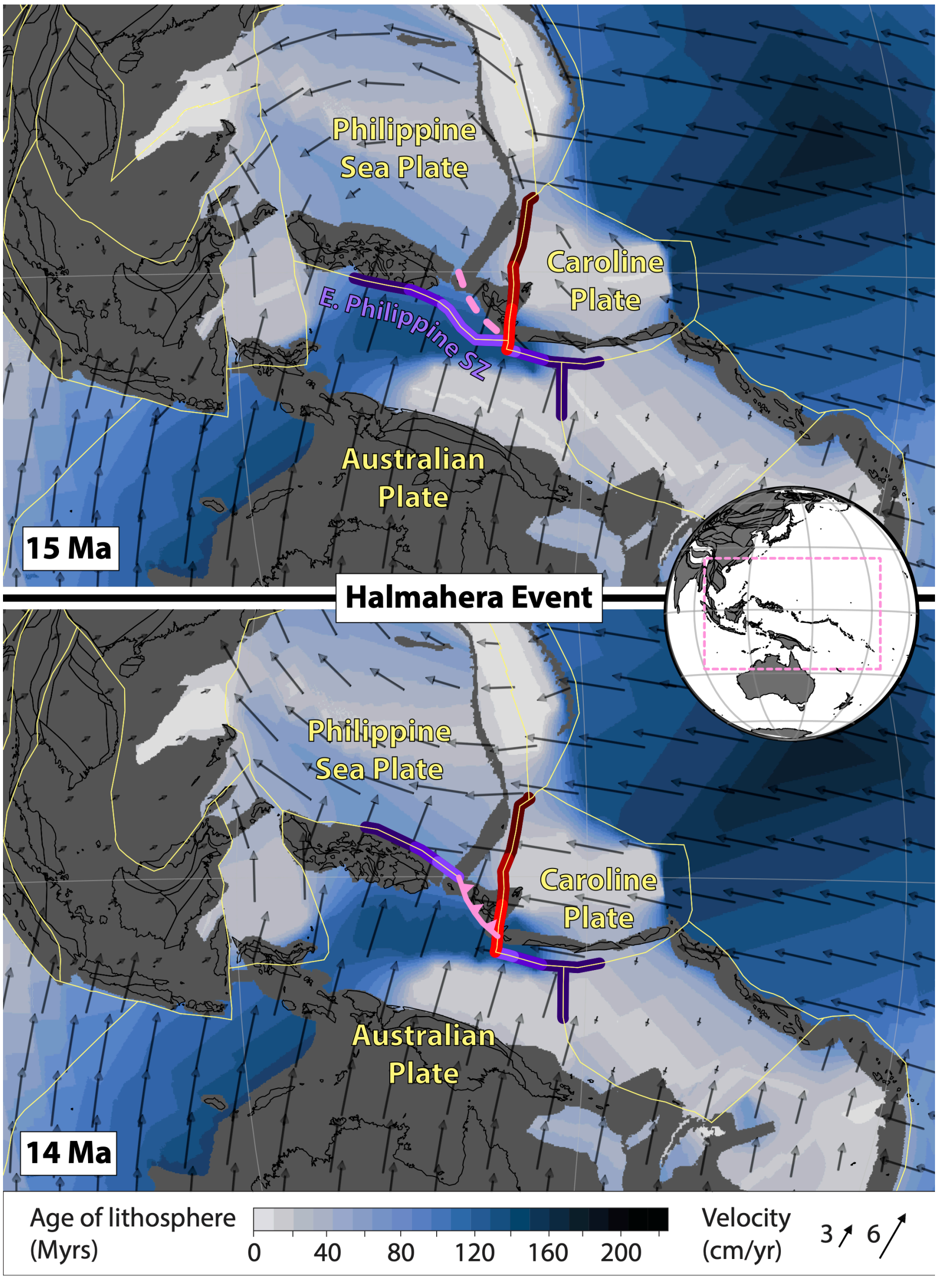

Supplementary Figure 12 Halmahera SZI event as reconstructed in the model of Müller et al.[7]. Pink dashed (solid with teeth) line shows the Halmahera trench 1 Myr before (at) SZI time in the model. Purple (red) lines show segments of neighbouring SZs (ridges and transforms) that lie within some radius of the Halmahera trench (pink line); the brightness of the colours reflects 3 different distance thresholds of 250, 500 and 1000 km.

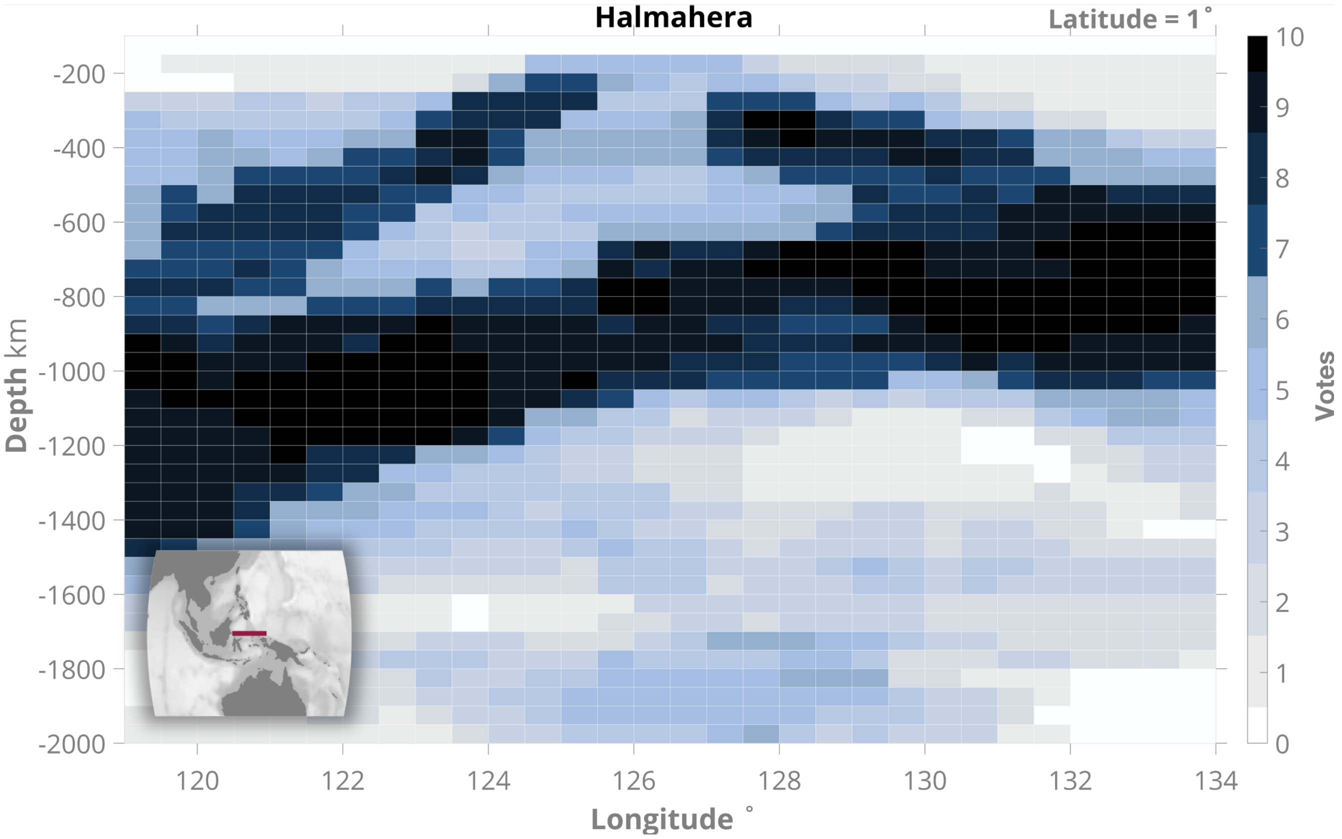

Supplementary Figure 13 Seismic tomography VoteMap[9] analysis of the Halmahera SZI event.

Supplementary Note 5: Izu-Bonin-Mariana

Interpretation. The onset of the presently active Izu-Bonin-Mariana (IBM) subduction zone likely occurred at around 52 Ma (Supplementary Figure 14) with the subduction of the Pacific plate under the Proto-Philippine Sea plate, which was mostly formed of arc terranes at the time of subduction initiation (e.g.,[38]). The age of this SZI event is mostly based on the age of the oldest *Early basalts* (e.g.,[39]), which are considered to be the first magmatic product of SZI and to erupt very soon after the onset of subduction.

The most common view is that subduction initiated along a pre-existing fracture zone after a plate reorganisation due to the subduction of the Izanagi-Pacific ridge beneath Asia at around 60–55 Ma[40,41] or the collision of the Olutorsky arc[4]. Regardless of the cause, these stress changes might have caused compression across a transform fault (or a pre-existing fracture zone) and locally initiated subduction[42]. Additionally, ocean-island basalt (OIB) magmatism in the West Philippine basin indicates the presence of a mantle plume (the Oki-Daito plume) that started its activity almost at the same time as the IBM SZI[43].

Direct evidence. The oldest age of *Early basalts* is around 51 Ma[39]. *Early basalts* are found along the entire length of the trench, suggesting that subduction started roughly at the same time everywhere along the IBM trench[44]. *Boninites* erupted soon after the *Early basalts* between around 51–44 Ma[39]. Afterwards, more typical tholeiitic arc lavas started to erupt at around 44 Ma[44].

Reconstruction. In the model of Müller et al.[7], the Izu-Bonin SZI event occurs at 52 Ma (see Supplementary Figure 15). The nucleation of the subduction zone occurs parallel to and generally within ~200 km of a pre-existing subduction zone ('Philippine Subduction') along which the Pacific plate subducted beneath the North Philippine Basin. The polarity of the Izu-Bonin subduction zone is the same as this pre-existing subduction zone. No major plate motions occur at ~52 Ma itself, but the motion of the North Philippine Basin plate changes significantly at 54 Ma (i.e., 2 Myr before the Izu-Bonin SZI event).

Seismic tomography. The Izu-Bonin and the Mariana slab appear to be separated today[8]. From a 3-D view of the anomalies on a vote map (see Supplementary Figure 16), it is clear that the two slabs are connected in the upper mantle, but while the Mariana slab goes straight down to the lower mantle, reaching around 1250 km depth, the Izu-Bonin slab has a shallower dip and it flattens out around the transition zone (reaching a maximum depth of 850 km).

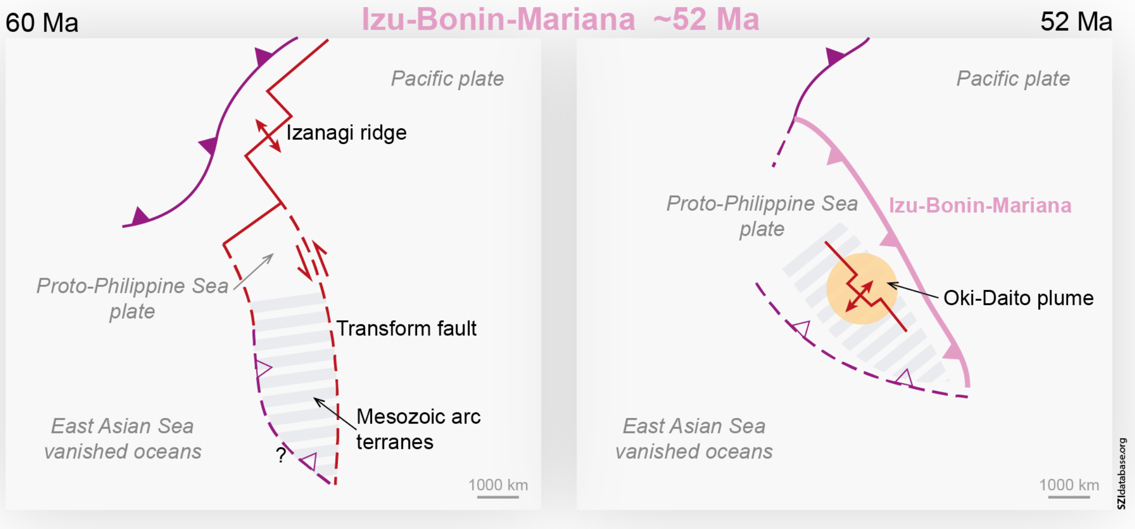

Supplementary Figure 14 Schematic tectonic reconstruction of the Izu-Bonin-Mariana SZI event modified from ref.[41]. A plate reorganisation, possibly due to the arrival of the Izanagi ridge at the trench, is suggested to trigger SZI along a pre-existing transform fault in the south, initiating the Izu-Bonin-Mariana subduction zone. The orange circle shows the location of the Oki-Daito plume. Shown are the new subduction zone (pink line), other active (solid purple lines) and inactive (dashed purple lines) subduction zones, spreading ridges (solid red lines), and transform faults (red dashed lines).

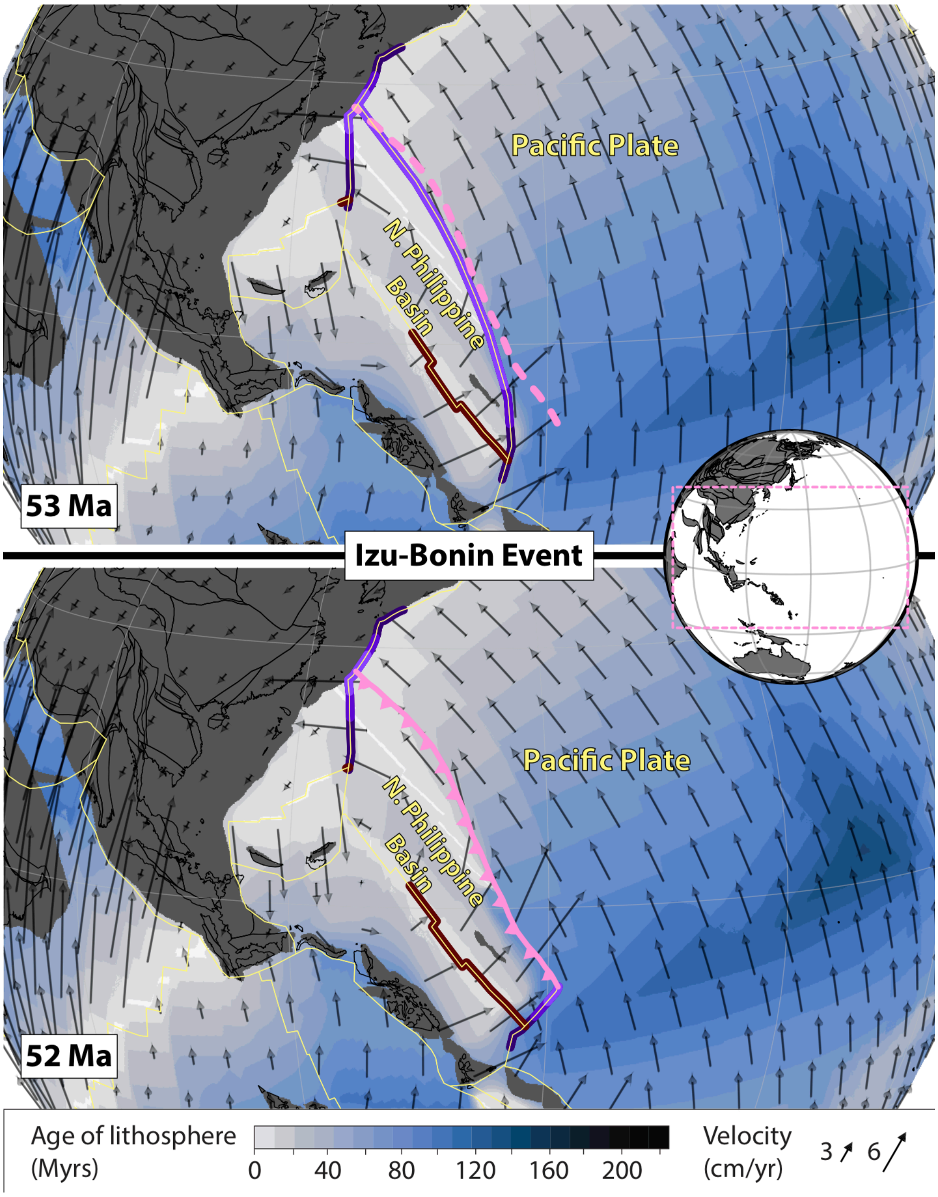

Supplementary Figure 15 Izu-Bonin SZI event as reconstructed in the model of Müller et al.[7]. Pink dashed (solid with teeth) line shows the Izu-Bonin trench 1 Myr before (at) SZI time in the model. Purple (red) lines show segments of neighbouring subduction zones (ridges and transforms) that lie within some radius of the Izu-Bonin trench (pink line); the brightness of the colours reflects 3 different distance thresholds of 250, 500 and 1000 km.

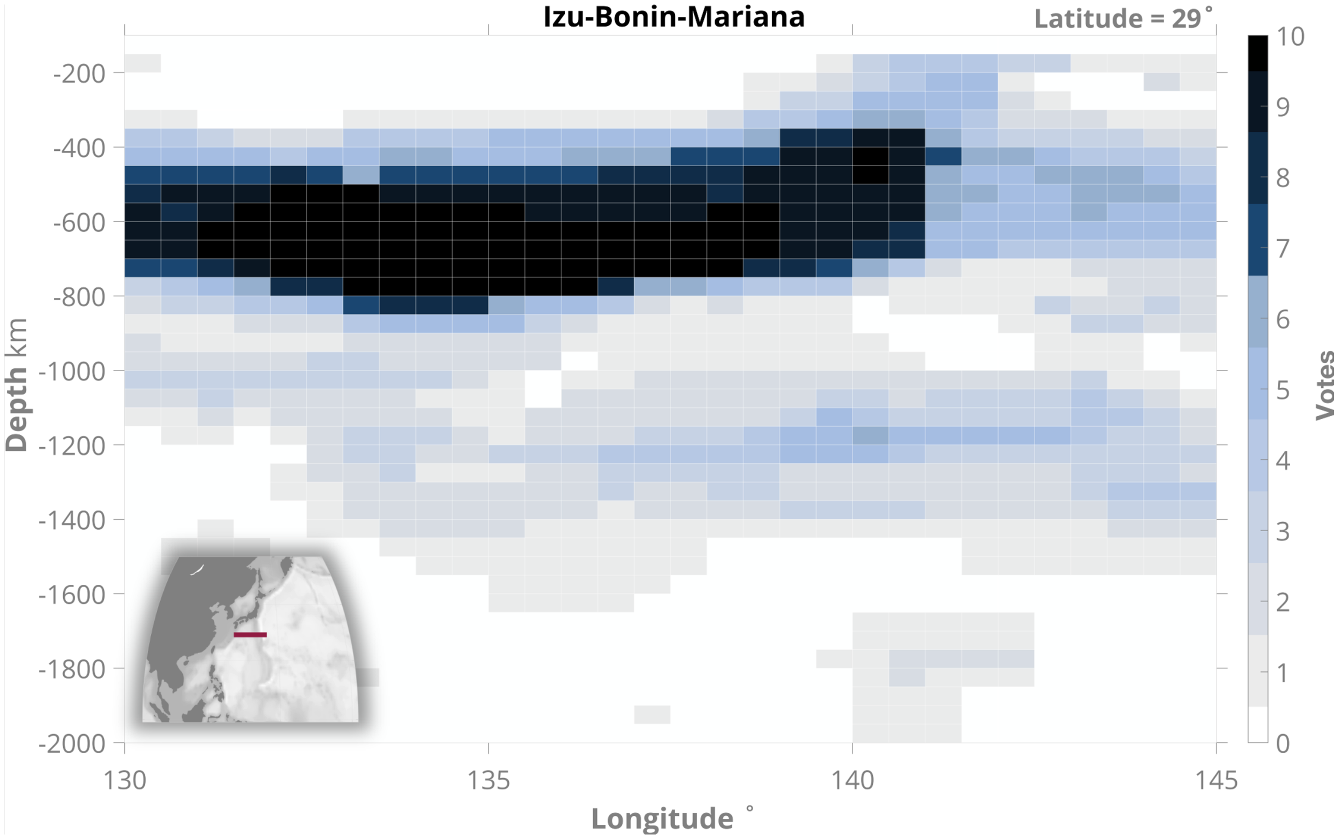

Supplementary Figure 16 Seismic tomography VoteMap[9] analysis of the Izu-Bonin-Mariana SZI event.

Supplementary Note 6: Lesser Antilles

Interpretation. The Lesser Antilles SZI event that formed the present-day Lesser Antilles subduction zone likely occurred between 59–38 Ma[45], with the North and South American plates subducting below the Caribbean plate (Supplementary Figure 17). However, there is a debate on the nature of this event, which also represents the transition from the Greater Caribbean Arc to the Lesser Antilles subduction zone. The break in the slab, revealed by seismic tomography[46], along with the age gap between the Aves Ridge and the Lesser Antilles Arc and the start of the formation of the Barbados Accretionary Prism[45] suggests episodic subduction. Other interpretations consider continuous subduction during the narrowing of the arc and suggest that the arc has jumped 50–250 km from the Avis ridge to the Lesser-Antilles arc during continuous subduction roll-back and the consequent opening of the Grenada and Tobago basins (together) as a forearc basin (e.g.,[47]). Due to the widening forearc, the Avis ridge became inactive. In this scenario, the SZI event of the Lesser Antilles is the same as that of the Greater Caribbean arc, which might have happened sometimes between 120 to 88 Ma. This earlier event is not considered here any further.

The Lesser Antilles SZI event might be an episodic event that followed from a previously active, but subsequently extinct, subduction zone; it is suggested that the active arc from the Aves ridge transitioned to, and formed, the Lesser Antilles arc during the mentioned time span[45].

Direct evidence. Ages of arc volcanism (K-Ar) in the Lesser Antilles Arc range from 38 Ma to present[48], while in the Aves ridge, volcanic rocks age between 88 to 59 Myr[49]. The Barbados prism, forming the accretionary prism of the Lesser Antilles subduction zone, was building up since the Early Eocene[50].

Reconstruction. In the model of Müller et al.[7], the Lesser Antilles SZI event occurs at 53 Ma (see Supplementary Figure 18). The Lesser Antilles subduction zone nucleated mostly parallel to and generally between 150 and 300 kms of a pre-existing subduction zone (unnamed in the model), along which the South American plate subducted beneath the Caribbean plate (with the same polarity as the Lesser Antilles subduction zone). The Caribbean plate exhibits a rather minor change in its plate motion at 55 Ma, but otherwise there are no significant plate reorganisations associated with the Lesser Antilles SZI event.

Seismic tomography. In the Atlas of the Underworld[8], the Lesser Antilles is connected to the Caribbean slab, which is imaged with P-wave tomography from below the surface to 750 km depth. A vote map cross-section through the Caribbean arc at 16° latitude shows a consistent fast (P- and S-wave) anomaly between 200 and 900 km depth (see Supplementary Figure 19). This anomaly cannot be differentiated from a lower-mantle anomaly towards the south.

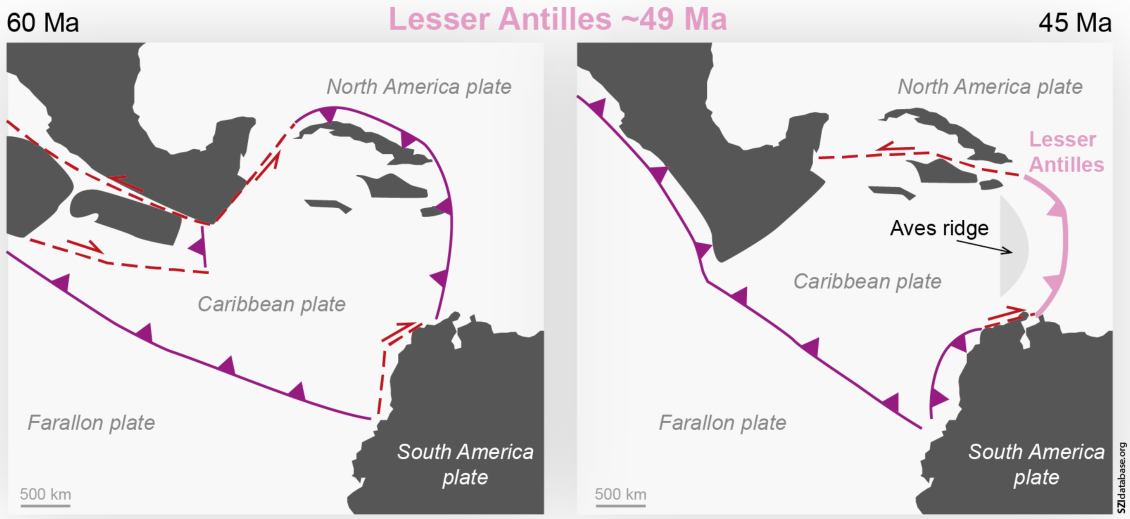

Supplementary Figure 17 Schematic tectonic reconstruction of the Lesser Antilles SZI event modified from ref.[45]. Subduction of the North and South America plates beneath the Caribbean plate was probably already active earlier on. At 58–39 Ma, subduction jumped eastwards, creating the new Lesser Antilles subduction zone. Shown are the new subduction zone (pink line), other active (solid purple lines) and inactive (dashed purple lines) subduction zones, and transform faults (red dashed lines).

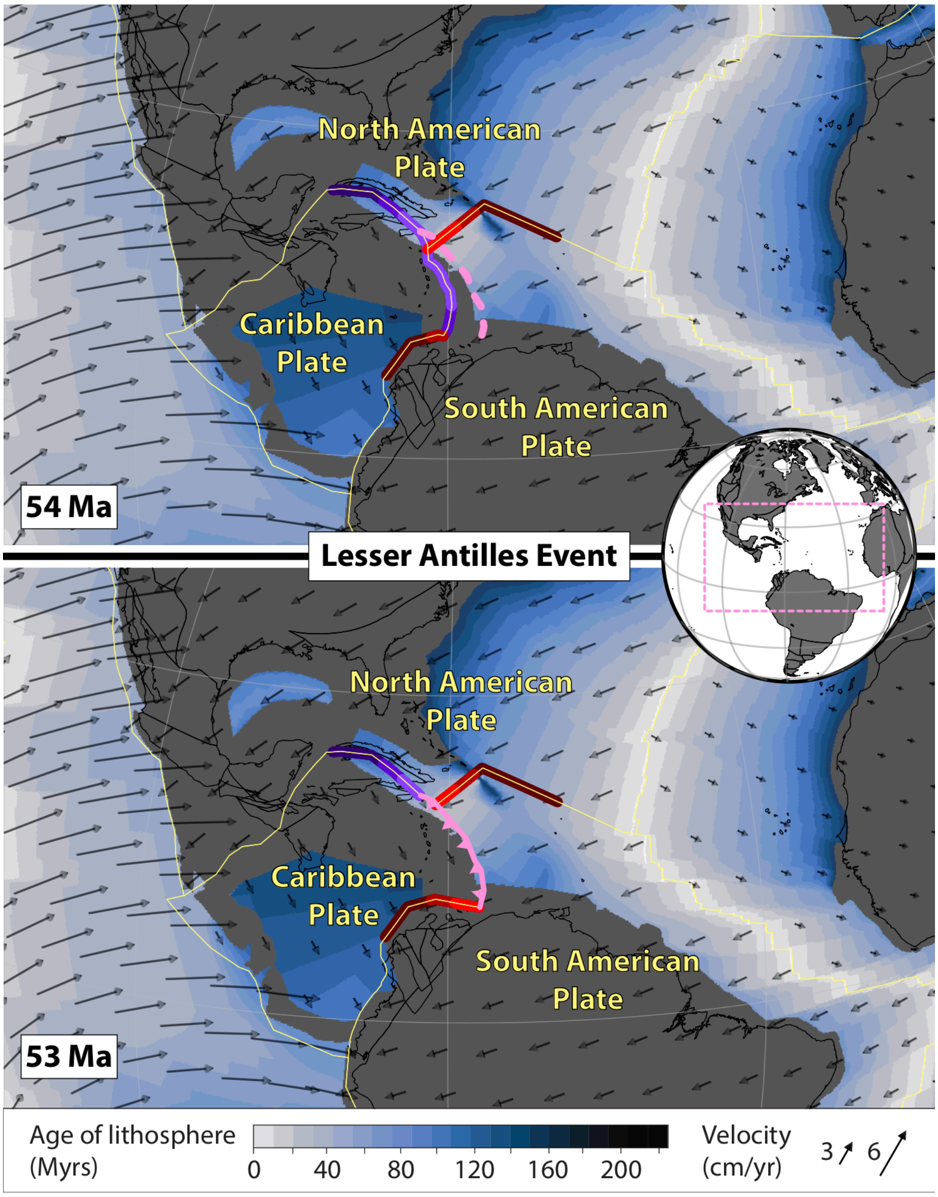

Supplementary Figure 18 Lesser Antilles SZI event as reconstructed in the model of Müller et al.[7]. Pink dashed (solid with teeth) line shows the Lesser Antilles trench 1 Myr before (at) SZI time in the model. Purple (red) lines show segments of neighbouring subduction zones (ridges and transforms) that lie within some radius of the Lesser Antilles trench (pink line); the brightness of the colours reflects 3 different distance thresholds of 250, 500 and 1000 km.

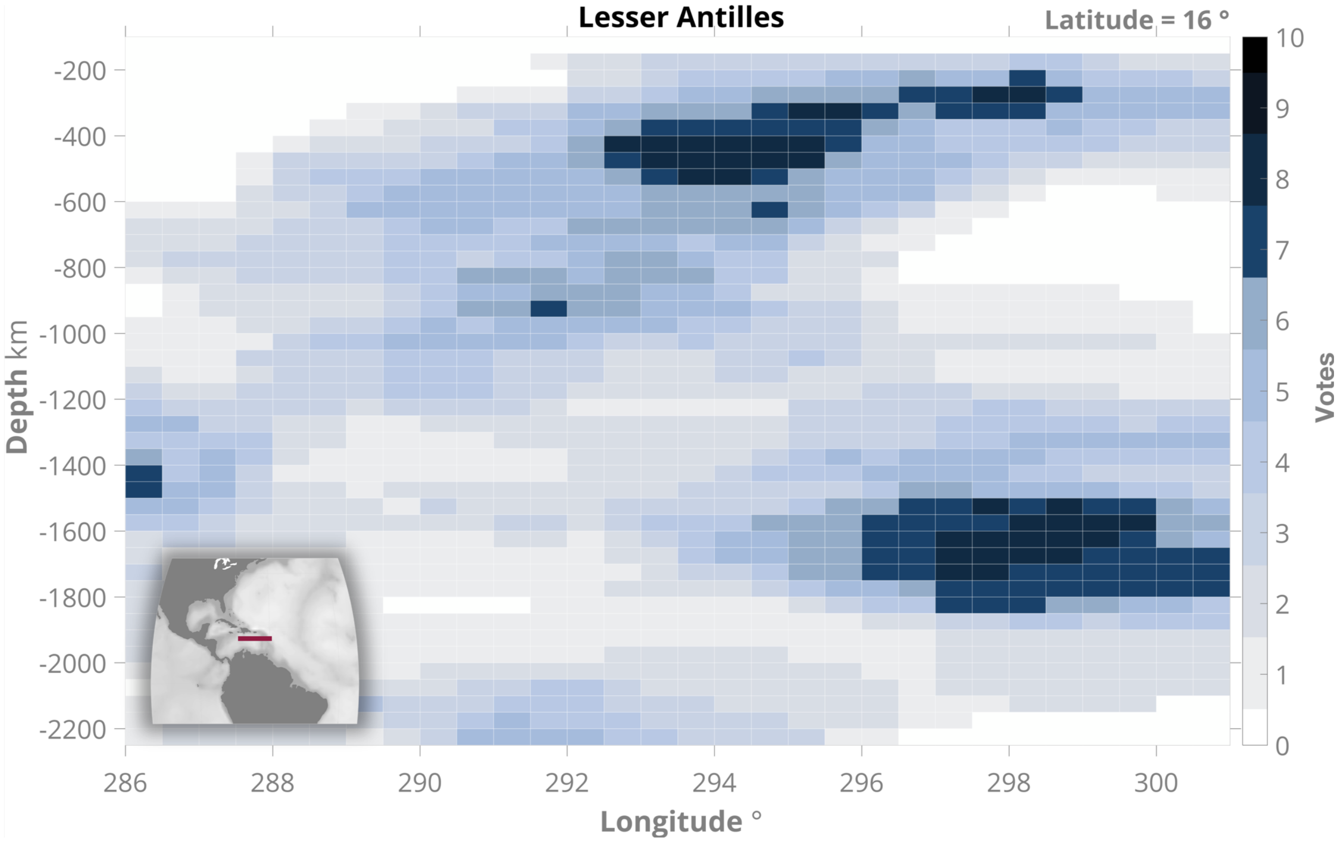

Supplementary Figure 19 Seismic tomography VoteMap[9] analysis of the Lesser Antilles SZI event.

Supplementary Note 7: New Hebrides-New Britain

Interpretation. The New Hebrides-New Britain (NHNB) SZI event evolved into the present-day subduction system that includes the New Hebrides, San Cristobal, and New Britain trenches (Supplementary Figure 20). The Australian plate currently subducts below a former portion of itself, lying to the north-east, that today makes up the North Fiji Basin (e.g.,[51]). These trenches are all currently connected to each other and initiated at similar times, which is why their onset is here attributed to one single event.

It has been suggested that the onset of the NHNB subduction zone, which is related to subduction of the Australian plate below the Pacific plate, originated by a reversal in subduction polarity at around 10 Ma. While some studies favour a time period for the SZI event between 10 and 6 Ma[52,53], others suggest an onset age of between 14–11 Ma[51,54]. This polarity reversal likely occurred as a result of the collision of the Ontong Java plateau with the Vitiaz trench (e.g.,[54,55]). The Ontong Java plateau lies on the Pacific plate that was subducting below the Australian plate during the collision, prior to the polarity reversal.

Direct evidence. The arc associated with this SZI event builds on the pre-existing arc of the south-dipping subduction of the Pacific plate (before the polarity reversal). Therefore, the oldest ages found in the New Hebrides arc (~25 Ma) are linked to the previous subduction zone[54]. At around 14–11 Ma the arc activity ceased. New arc volcanism starts again at around 6 Ma, this time related to the New Hebrides subduction[54,56]. Similarly, along the New Britain trench, the subduction of the Pacific plate below the Australian plate built an arc (43–26 Ma). Afterwards, a gap in volcanic activity is observed between 20–12 Ma. Finally, arc volcanism associated with the new subduction zone (i.e., after the polarity reversal) started again at around 10–5 Ma[55].

Timing of SZI is based on the opening of the North Fiji basin, which is interpreted as a backarc basin behind the New Hebrides subduction. Paleomagnetic data in different locations of the New Hebrides arc display a 28–52° clockwise rotation of the eastern limb of the arc since the Late Miocene in response to the opening of the North Fiji basin[57,58]. Magnetic seafloor anomalies identified in the North Fiji basin constrain the initial phase of basin opening to around 12–8 Ma[53,59].

Reconstruction. In the model of Müller et al.[7], the New Hebrides-New Britain SZI event occurs at 12 Ma (see Supplementary Figure 21). This subduction zone initiated on the south side of an island arc that had a pre-existing subduction zone along its northern margin ('Pacific Melanesia subduction zone' in the model). The subduction zone on the north margin of the island arc, which had a south-dipping polarity (i.e., opposite to that of the New Hebrides-New Britain subduction zone), shutdown at the same time that the New Hebrides-New Britain subduction zone started (12 Ma). Thus, in this model, the New Hebrides-New Britain SZI can be seen as a subduction polarity inversion. The distance separating these subduction zones was ~200–350 km. In conjunction with this SZI event, the motion of the Solomon Sea plate changed at 12 Ma, but no major plate reorganisations occurred at or immediately before 12 Ma; the motion of the Australian and Pacific plates, for example, remained unchanged during this time.

Seismic tomography. The New Hebrides and the New Britain slabs are both imaged and cataloged in the Atlas of the Underworld[8]. Both anomalies reach the upper-lower mantle boundary. However, the New Britain slab (575 km deep) seems to be shallower than the New Hebrides slab (675 km deep). Compared to a vote map, we find a consistent fast anomaly until around 800 km depth (see Supplementary Figure 22).

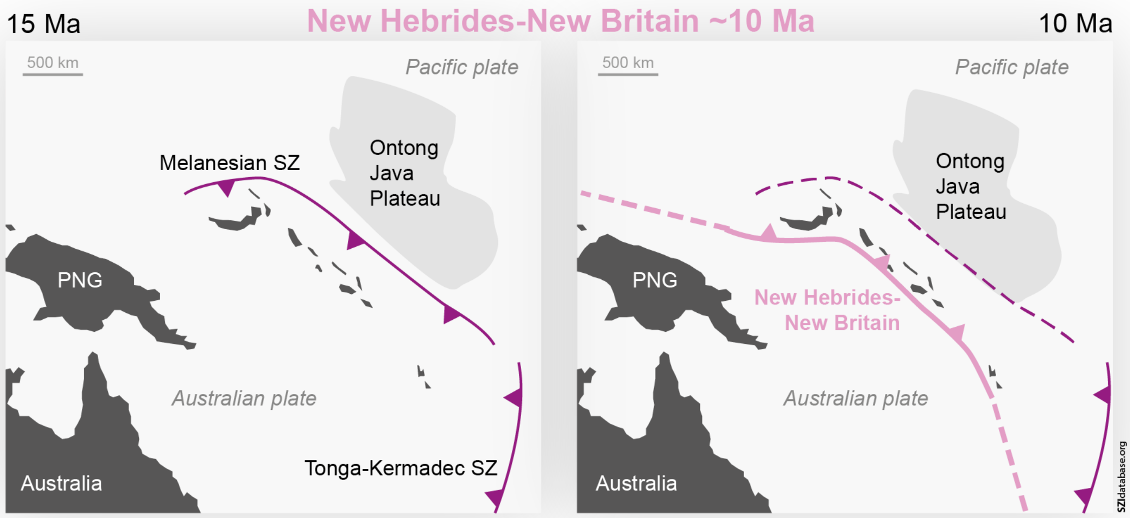

Supplementary Figure 20 Schematic tectonic reconstruction of the New Hebrides-New Britain SZI event modified from refs.[51,60]**.** The collision of the Ontong Java plateau with the trench of the Melanesian subduction zone is suggested to have caused a flip in subduction polarity, initiating the New Hebrides-New Britain subduction zone. Shown are the new subduction zone (pink line) and other active (solid purple lines) and inactive (dashed purple lines) subduction zones.

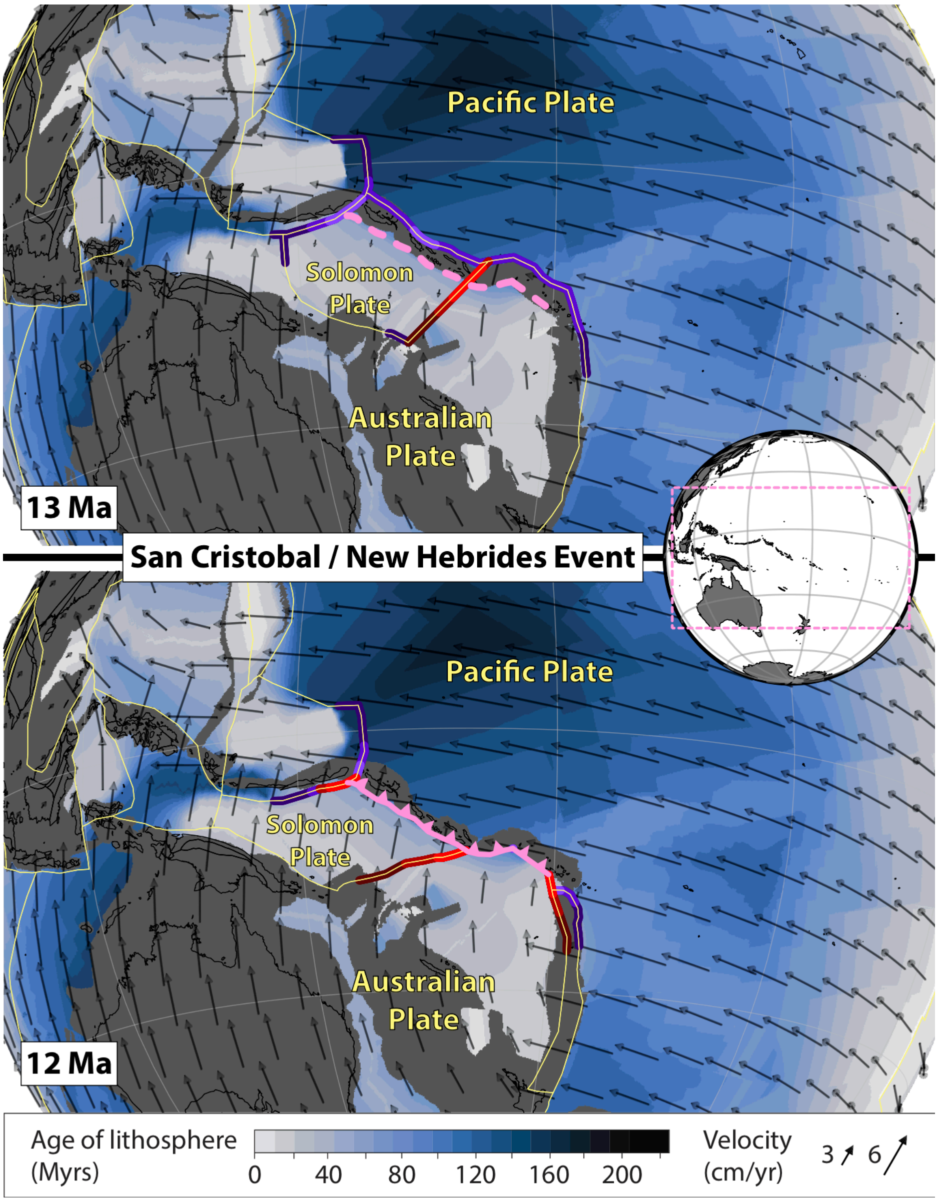

Supplementary Figure 21 New Hebrides-New Britain SZI event as reconstructed in the model of Müller et al.[7]. Pink dashed (solid with teeth) line shows the New Hebrides-New Britain SZI trench 1 Myr before (at) SZI time in the model. Purple (red) lines show segments of neighbouring subduction zones (ridges and transforms) that lie within some radius of the New Hebrides-New Britain SZI trench (pink line); the brightness of the colours reflects 3 different distance thresholds of 250, 500 and 1000 km.

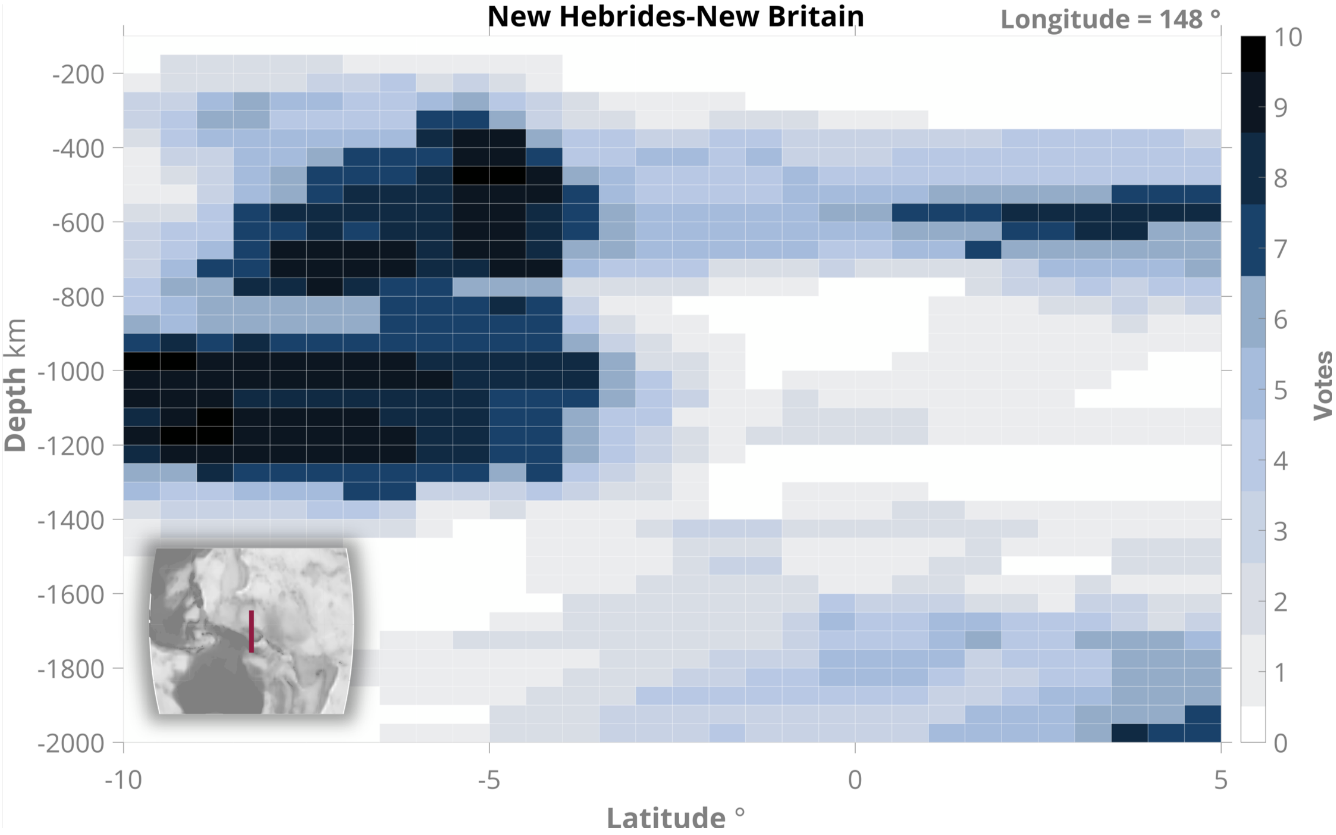

Supplementary Figure 22 Seismic tomography VoteMap[9] analysis of the New Britain-New Hebrides SZI event.

Supplementary Note 8: Oman

Interpretation. The Oman subduction zone, together with the Anatolian subduction zone, formed the Western Neotethyan subduction system (Supplementary Figure 23). The Oman SZI event was widely thought to have initiated along, or in the vicinity of, a Neotethyan mid-oceanic ridge (e.g.,[61,62,63]). Recently, it has been suggested that the subduction zone initiated along a fracture zone, located parallel to the Arabian continent[10,64].

The subduction zone seems to have initiated at 104 Ma (e.g.,[20]) within Neotethyan oceanic lithosphere (similar to the Anatolia SZI (see *Anatolia* SZI event in the SZI database), but with the opposite vergence[10]. At the time of SZI, both downgoing and overriding plates were oceanic lithosphere of the Neotethys. In the case of Oman (and in contrast to the Anatolian subduction zone), the 'Anadolu plate'[12] subducted below the Africa-Arabia continental plate (i.e., 'Greater Adria' of Gaina et al.[13] and van Hinsbergen et al.[10],[14]). The subduction zone later terminated and resulted in widespread ophiolite obduction onto the Arabian continental margin in the Late Cretaceous at 70±5 Ma, represented by the Semail ophiolite of Oman, the Kermanshah and Neyriz ophiolites of Iran, the Baer Bassit ophiolite of Syria, the Hatay ophiolites of SE Turkey, and perhaps the Troodos ophiolite of Cyprus[62,65,66,67,68,69,70,71].

Direct evidence. The oldest subduction-related products are represented by the plutonic section of the Semail ophiolite, which indicate that the ophiolitic crust formed at a fast spreading ridge in less than 1 Myr in the Late Cretaceous (zircon U-Pb ages of ~96–95 Ma; Rioux et al.[72]). *Boninites* are found in the Alley unit (also called V2) and are interbedded with tholeitic lavas (which have similarities with the Izu-Bonin-Mariana *Early basalts*). The age of the V2 unit is ~95 Ma[72,73]. Basaltic andesites are also found in the Alley unit V2[74], which are the products of the formation of the arc. Garnet Lu-Hf ages from the metamorphic sole constrain subduction-related prograde metamorphism to 104 Ma[20]. The time lag between prograde metamorphism and the crystallisation of supra-subduction forearc crust argue for a horizontally-forced SZI event[10,20]. The cause for this horizontally-forced SZI event within the western Neotethys remains speculative and matter of debate[10,71].

Reconstruction. The model of Müller et al.[7] does not implement this SZI event; instead, Oman is modelled as a passive margin during the Early Cretaceous, adjacent to a northeast-oriented transform ("Proto-Owen Fracture Zone") running sub-parallel to the southeast margin of the Arabian subcontinent. From approximately 125 Ma, a southwest-facing intra-oceanic subduction zone ("Western Tethys intra-oceanic subduction zone") began migrating toward the southwest from the southern margin of Eurasia (being separated from the latter by a backarc spreading system), and by ~85 Ma that intra-oceanic arc had collided with the passive margin of Oman. Following that collision, the margin of Oman remained passive until the Miocene, when it collided with the "Western Tethys subduction zone" along the southern margin of Eurasia.

Seismic tomography. The Arabia slab of the Atlas of the Underworld[8] has been identified to be related to the Oman SZI event, and its base age was recently updated to 105–102 Ma. It is supported in the vote maps, which shows a positive wavespeed anomaly consistently from 1100 km to 2000 km depth.

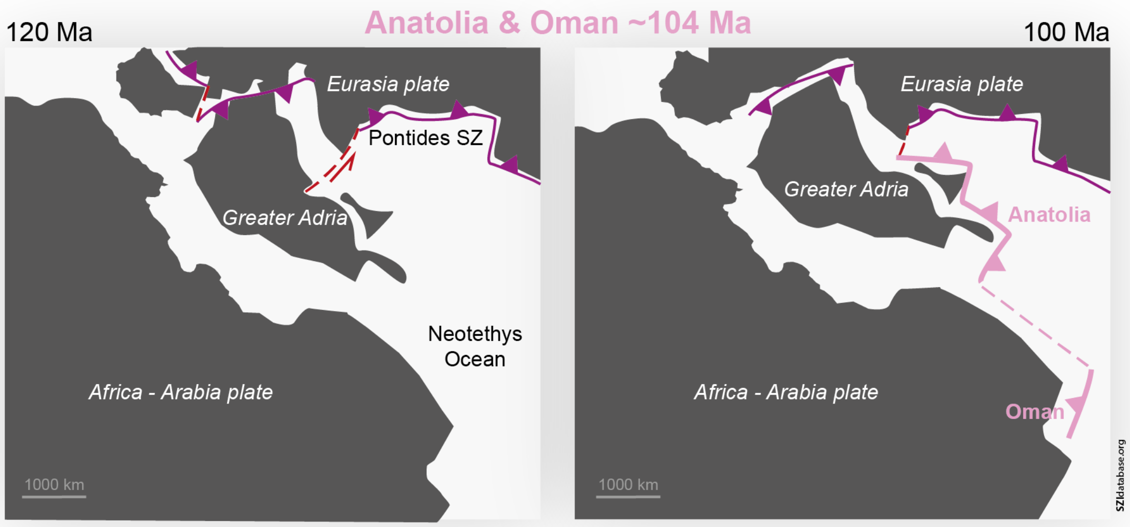

Supplementary Figure 23 Schematic tectonic reconstruction of the Oman SZI event modified from ref.[10]. Shown are the new subduction zone (pink line), other active subduction zones (solid purple lines) and inactive, and transform faults (red dashed lines).

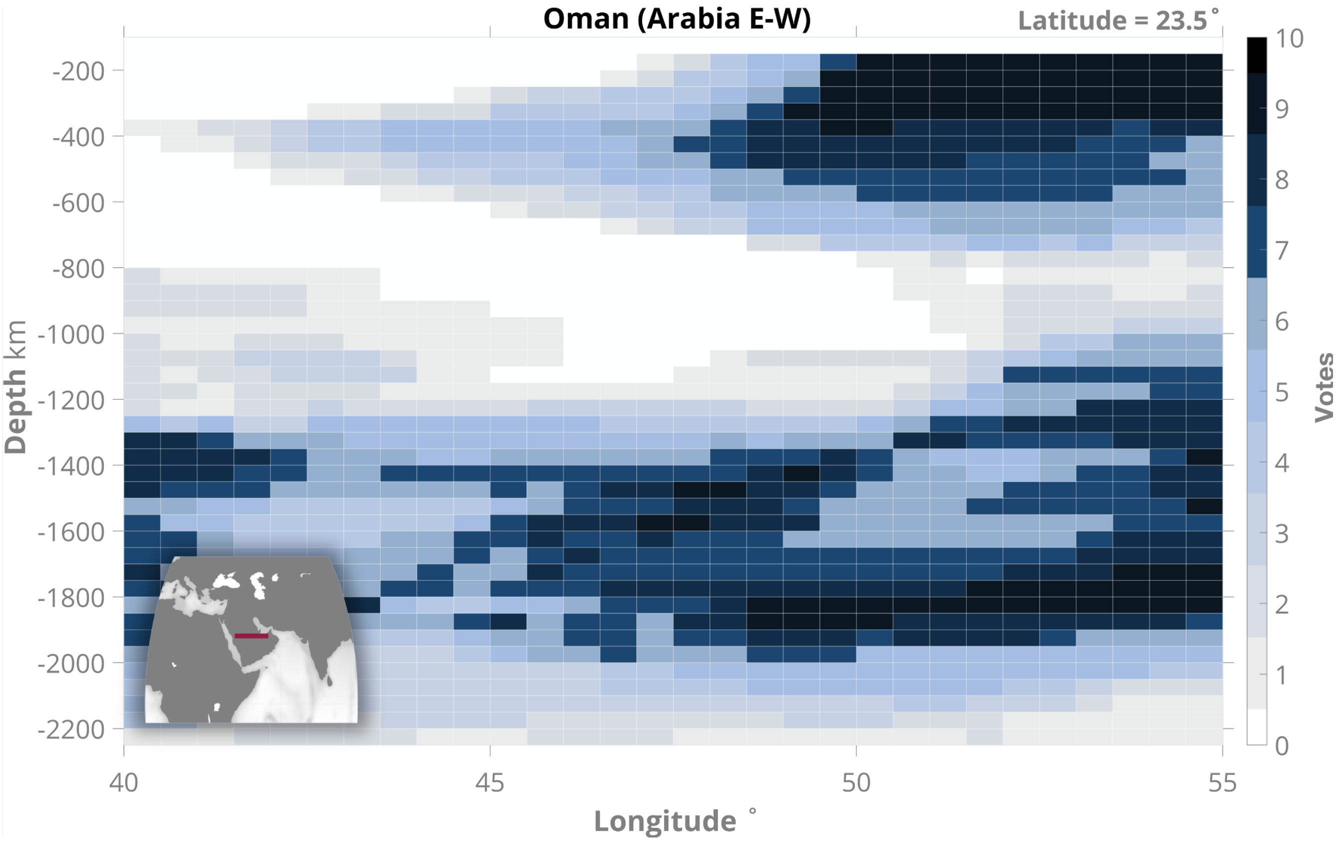

Supplementary Figure 24 Seismic tomography VoteMap[9] analysis of the Oman SZI event.

Supplementary Note 9: Philippine

Interpretation. The present-day Philippine subduction zone, with the Philippine Sea plate subducting below the Eurasian plate (Supplementary Figure 25), is thought to have started at about 9 Ma (e.g.,[36]) after the collision of the Palawan continental block with the Philippine Mobile Belt (PMB) that occurred around 20–11 Ma[75,76].

The Palawan block belongs to the Eurasian plate and drifted towards the southeast until it collided with the Philippine archipelago or, in other words, the former trench of the former, eastward subduction zone[75]. This collision likely induced a flip in subduction polarity, which initiated the westward Philippine subduction zone[77] on the other side of the already existing volcanic arc.

Direct evidence. Arc volcanism related to the east dipping subduction along the Philippine trench started at the latest around 6.6 Ma and is today recorded in rhyolitic volcanic rocks the Bicol peninsula (i.e., in the northern part of today's subduction trench). Arc magmatism shows a trend towards younger ages both north (1.5 Ma) and south of Bicol (3.5 Ma), potentially indicating a southward propagation of the subduction trench[78,79].

Reconstruction. In the model of Müller et al.[7], the Philippine SZI event occurs at 14 Ma, in conjunction with the Halmahera SZI event (the subduction zones are contiguous but of opposite polarity; see Supplementary Figure 26). The initiation of the Philippine subduction zone immediately follows the cessation of a pre-existing subduction zone (the 'East Philippine' subduction zone) on the far (south) side of the Philippine arc; the Philippine subduction zone initiated on the north side of the arc, parallel to the orientation of the pre-existing subduction zone, but separated from it by ~350–500 km. Coincident with the SZI event (at 14 Ma), the motion of the Philippine Sea plate changes significantly, whereas the motion of the Australian plate remains unchanged.

Seismic tomography. The scenario with the collision-induced subduction polarity flip is also consistent with the tomographic image under the Bicol peninsula[80]. Although the Philippine slab is missing from the Atlas of the Underworld catalogue, the seismic tomography models used here for creating vote maps are consistent in predicting a slab until 700–800 km depth, where it overlaps with another deeper anomaly (see Supplementary Figure 27).

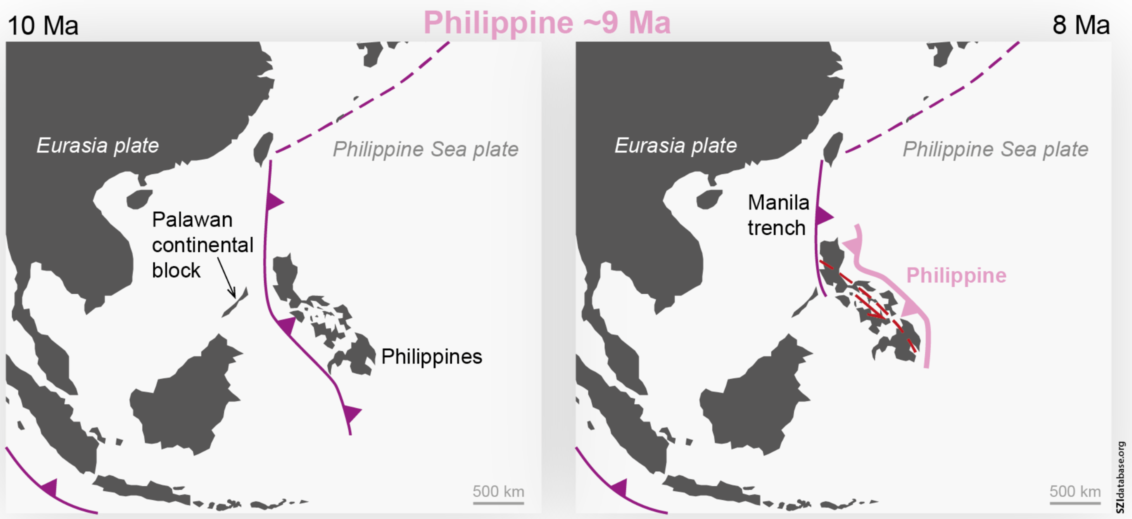

Supplementary Figure 25 Schematic tectonic reconstruction of the Philippine SZI event modified from refs.[31,36]. The collision of the Palawan continental block with the trench of the east-dipping subduction of the Eurasia plate below the Philippine Sea Plate is suggested to have caused a flip in subduction polarity, initiating the new Philippine subduction zone. Shown are the new subduction zone (pink line) and other active (solid purple lines) and inactive (dashed purple lines) subduction zones.

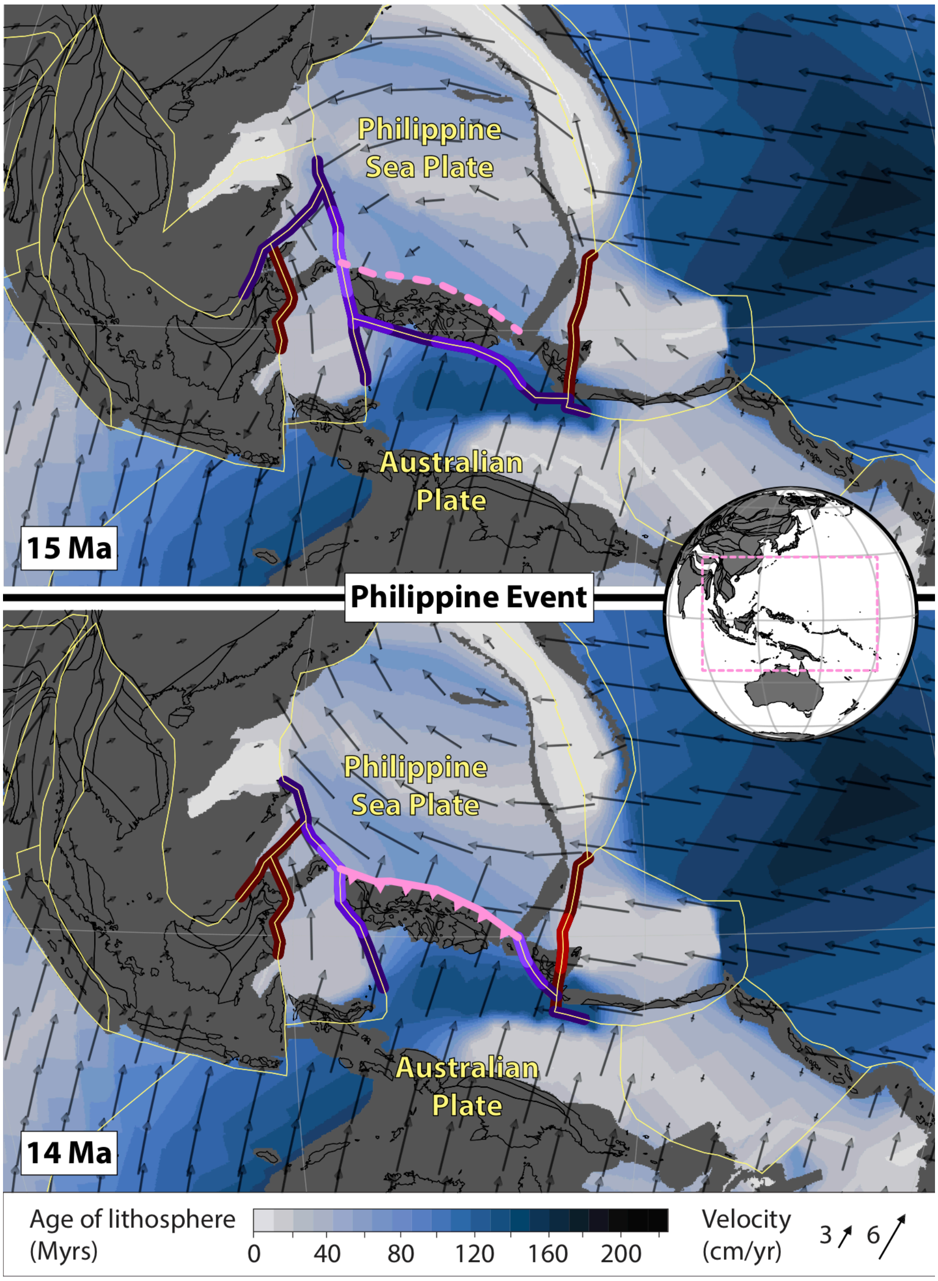

Supplementary Figure 26 Philippine SZI event as reconstructed in the model of Müller et al.[7]. Pink dashed (solid with teeth) line shows the Philippine trench 1 Myr before (at) SZI time in the model. Purple (red) lines show segments of neighbouring subduction zones (ridges and transforms) that lie within some radius of the Philippine trench (pink line); the brightness of the colours reflects 3 different distance thresholds of 250, 500 and 1000 km.

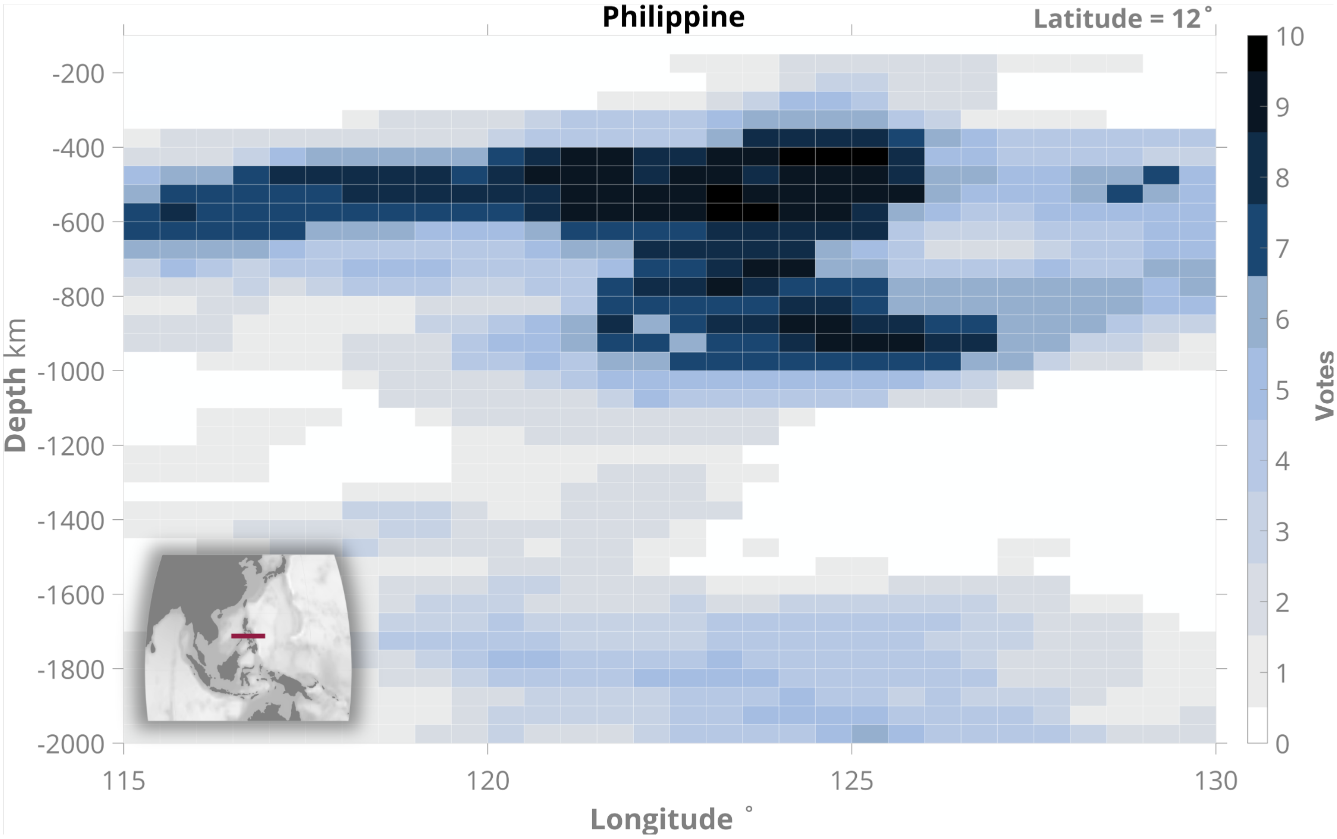

Supplementary Figure 27 Seismic tomography VoteMap[9] analysis of the Philippine SZI event.

Supplementary Note 10: Ryukyu

Interpretation. The Ryukyu SZI event reinitiated subduction of the Philippine Sea plate below the Eurasian plate, and is presently characterised by a northwest-dipping slab along the western boundary of the Philippine Sea plate (Supplementary Figure 28). For Ryukyu, there seem to be two SZI events to consider, an older more enigmatic event and a subsequent, younger one. The first, older SZI event is unclear, as reconstruction models currently disagree (e.g., Faccenna et al.[81] versus Müller et al.[7]) and geologic evidence is largely missing. The second, younger SZI event, which is considered and named here Ryukyu SZI event, might be classified as an episodic SZI event that occurred at around 6 Ma. The two separated phases of ongoing subduction are interrupted by a slab break-off event[82,83], due to the arrival of the Gagua Ridge at the subduction trench[84].

Direct evidence. Ryukyu arc volcanism, indicating ongoing subduction, is observed to have occurred during two separate time periods, between 18–13 Ma and from 6 Ma until present day[81,85,86]. For the youngest episode of magmatism, the oldest ages of arc rocks are reported to be about 6 Ma and are found in Kume Island and Aguni[86,87].

Reconstruction. The SZI event is not implemented into the current plate reconstruction model[7].

Seismic tomography. The Ryukyu slab is imaged by van der Meer et al.[8] in the Atlas of the Underworld. It is represented by a fast anomaly from the surface until 350 km depth. The Ryukyu slab is underlain by the Pacific slab subducting at the Izu-Bonin-Mariana trench. Due to its shallow depth, this anomaly does not show on a vote map (see Supplementary Figure 29).

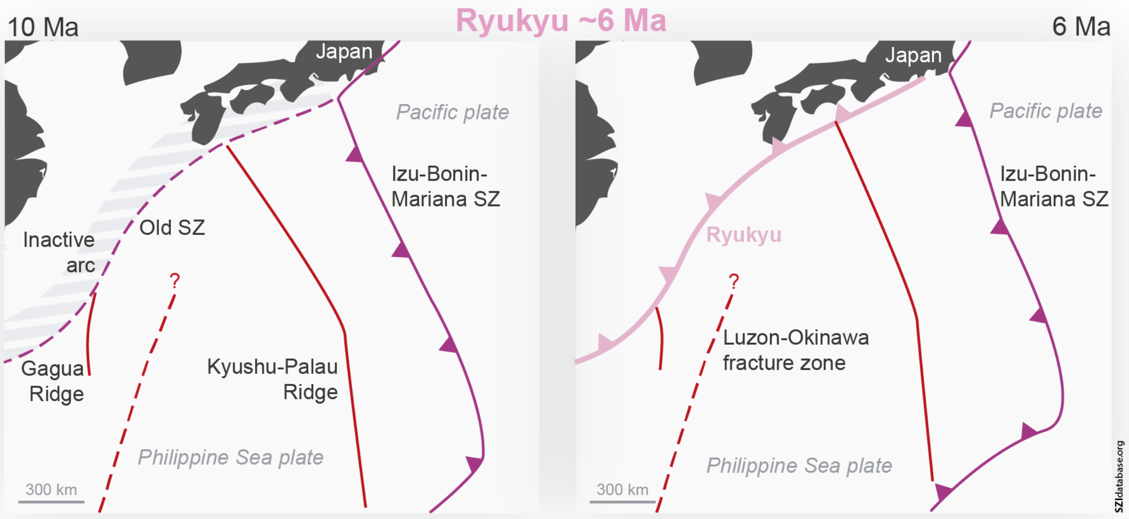

Supplementary Figure 28 Schematic tectonic reconstruction of the Ryukyu SZI event modified from ref.[81]. A slab break-off event caused a pause in arc activity. Subduction of the Philippine Sea plate started again along the same margin, initiating the new Ryukyu subduction zone. Shown are the new subduction zone (pink line), other active (solid purple lines) and inactive (dashed purple lines) subduction zones, and ridges (solid red lines).

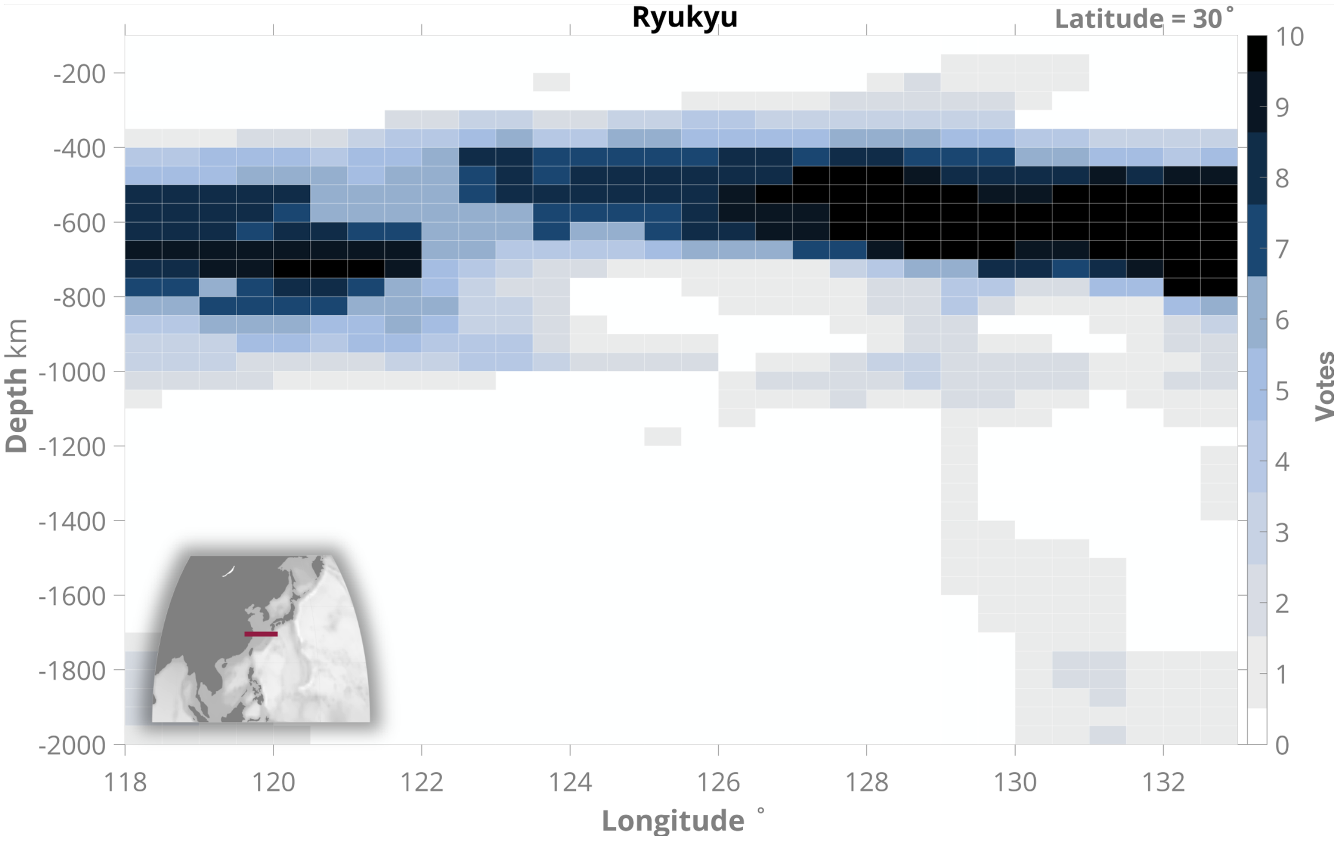

Supplementary Figure 29 Seismic tomography VoteMap[9] analysis of the Ryukyu SZI event.

Supplementary Note 11: South Sandwich

Interpretation. The South Sandwich SZI event marked the start of the subduction of the South American plate westwards beneath the Scotia plate, giving rise to the South Sandwich subduction zone that remains active at present-day (Supplementary Figure 30). The age of that SZI event remains debated, with estimates ranging from ~30 Ma to the Cretaceous[88,89] and, from the cross-disciplinary perspective, we estimate SZI to have occurred between 39 and 29 Ma. The type of SZI associated with the onset of South Sandwich subduction is interpreted as a new destructive boundary after e.g.,[89]. South Sandwich subduction has, however, also been attributed to lateral propagation from the Endurance Collision Zone[88] - in which case the event would not actually qualify as SZI, according to our definition. More broadly (on a larger scale), the South Sandwich SZI might be a consequence of subduction polarity reversal[90]. In this interpretation, the South Sandwich SZI occurred as a subduction polarity reversal further back in time (between around 80-40 Ma) along one section of the previously intact South America-South Shetland subduction system[90], possibly by collision of the Chile ridge with the preexisting subduction trench[91]. The South Sandwich SZI event might have coincided with a reconstructed acceleration of westward motion of the South America plate relative to the Africa plate[91].

Direct evidence. The oldest dated arc magmatic rocks associated directly to the South Sandwich system are calc-alkaline basalts, basaltic andesites and andesites dredged from the South Sandwich forearc, which have yielded K-Ar ages of 28.5 ± 0.9 Ma to 32.8 ± 3.2 Ma[92,93]. Similar Ar-Ar ages (28.5 ± 1.4 and 28.6 ± 1.0 Ma) have also been reported from basalts dredged from volcanic edifices in the Central Scotia Sea, which Dalziel et al.[93] and Pearce et al.[89] have termed the "Ancestral South Sandwich Arc" (ASSA). Together, those ~33–28 Ma volcanic rocks indicate that a subduction-related magmatic arc must have been operating at least by 33 Ma, implying that SZI had occurred prior to that time. Geometric considerations also require that subduction was operating by the time the West Scotia Sea started spreading (as it outpaced the rate of separation between South America and Antarctica), which Pearce et al.[89] have estimated to have begun by 32 ± 2 Ma on the basis of marine magnetic anomaly data. These observations thus place an upper age constraint of ~34 Ma on the timing of SZI.

The South Sandwich SZI is suggested to be of an Andean type in which the oldest lavas just postdate subduction initiation. This is due to the lack of crust (ophiolite) that might otherwise represent new lithosphere formed by subduction initiation rollback, as well as the lack of a boninitic component among the earliest lavas[89]. Pearce et al.[89] also noted an isotopic signature of Pb and Hf enrichment among the oldest arc volcanics, which they interpreted to reflect higher subduction temperatures associated with a hot mantle wedge that had not been cooled by prior subduction there.

Reconstruction. In the model of Müller et al.[7], the South Sandwich SZI event occurs at 30 Ma (see Supplementary Figure 31). The southern half of the South Sandwich subduction zone exploits a pre-existing boundary where convergence was already occurring (labeled as the 'Endurance Collision Zone', ECZ, in the model), whereas the northern half of the South Sandwich subduction zone develops away from active plate boundaries. There are no major plate tectonic reorganisation events associated with this region at this time, but after initiation of the South Sandwich subduction zone, the motion of the (then-enlarged) Central Scotia Sea plate (CSS; model plate ID 818) changes at 29 Ma.

Seismic tomography. The South Sandwich subduction zone is represented by a west-dipping fast seismic anomaly connected to the surface and reaching a depth of 850 km (±200km)[8]. Based on a vote map, seismic tomography models consistently predict a fast velocity anomaly until a depth of 1000 km (see Supplementary Figure 32). Some models cannot resolve the top of the anomaly, hence it is only visible below 400 km depth on the vote map.

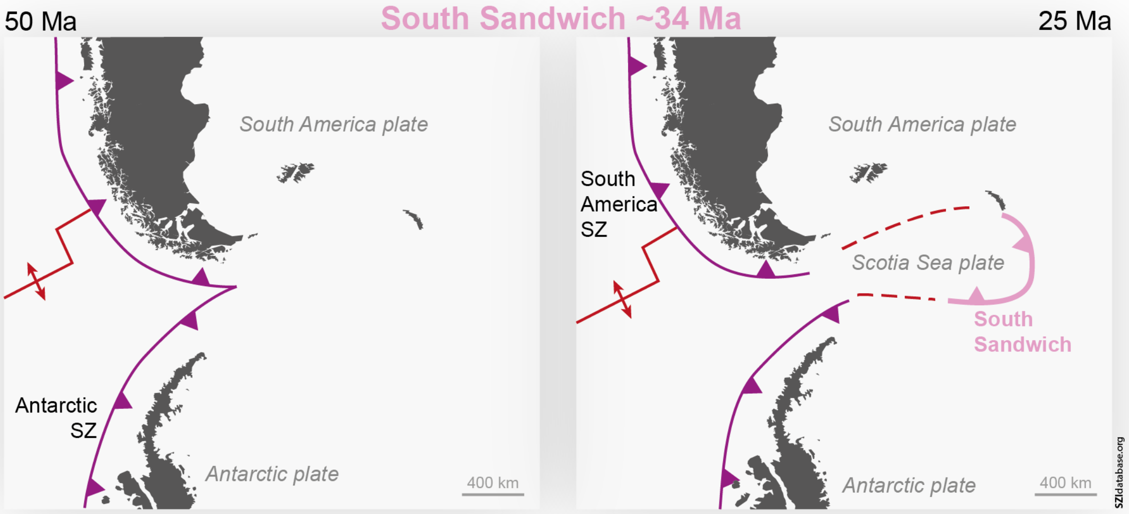

Supplementary Figure 30 Schematic tectonic reconstruction of the South Sandwich SZI event modified from ref.[94]. The arrival of the Chile ridge at the South America trench might have triggered a flip in subduction polarity, but the South Sandwich subduction zone is suggested to have initiated as a newly destructive boundary. Shown are the new subduction zone (pink line), other active subduction zones (solid purple lines), spreading ridges (solid red lines), and transform faults (red dashed lines).

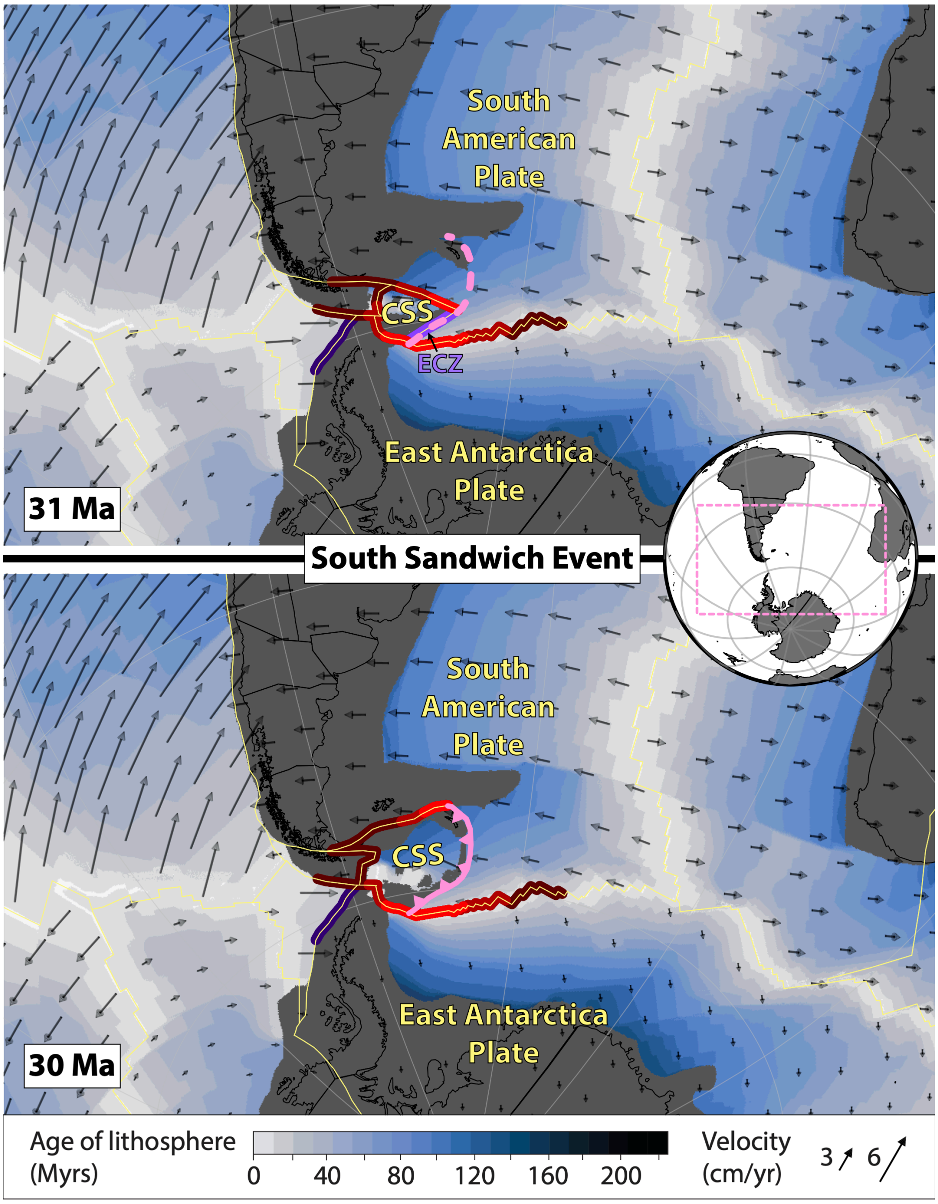

Supplementary Figure 31 South Sandwich SZI event as reconstructed in the model of Müller et al.[7]. Pink dashed (solid with teeth) line shows the South Sandwich trench 1 Myr before (at) SZI time in the model. Purple (red) lines show segments of neighbouring subduction zones (ridges and transforms) that lie within some radius of the South Sandwich trench (pink line); the brightness of the colours reflects 3 different distance thresholds of 250, 500 and 1000 km.

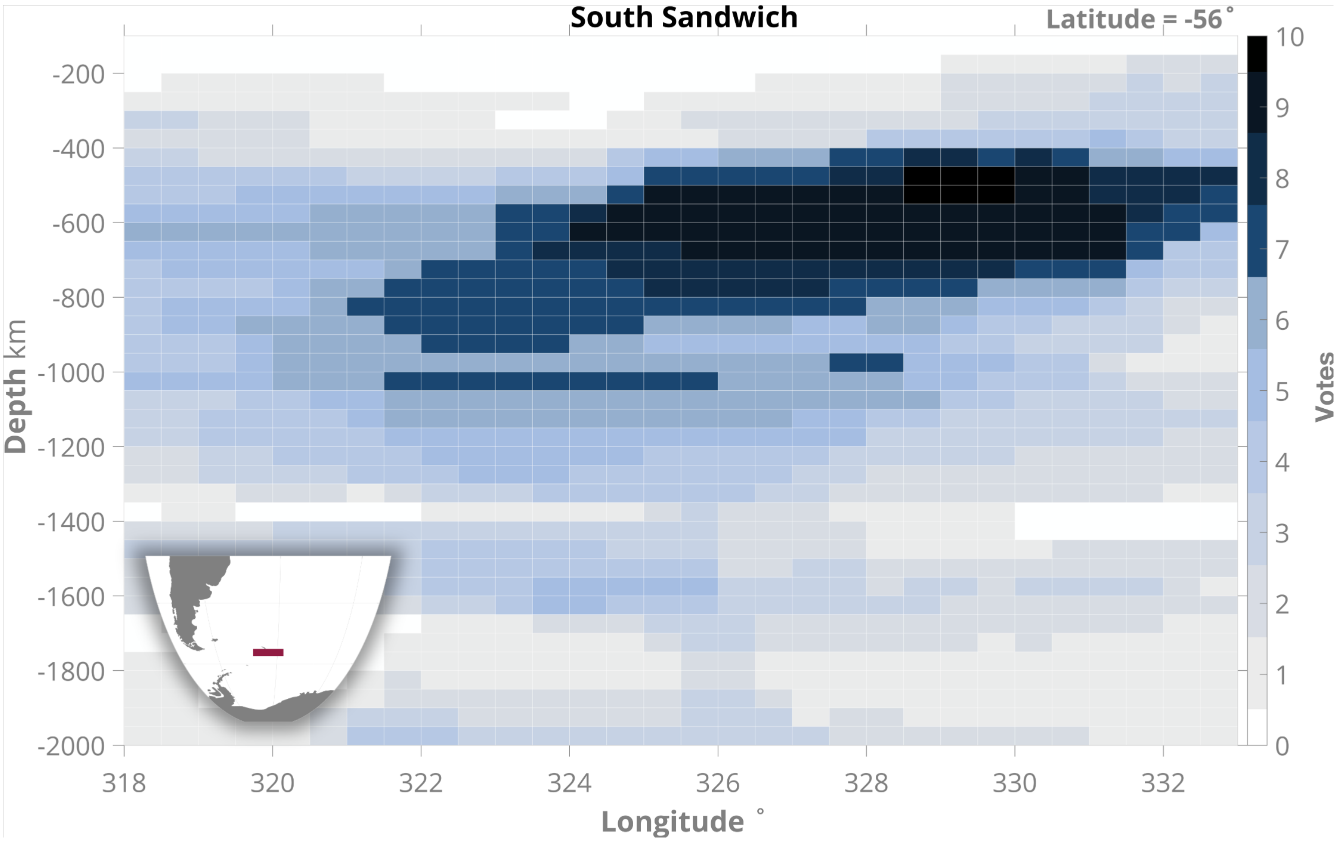

Supplementary Figure 32 Seismic tomography VoteMap[9] analysis of the South Sandwich SZI event.

Supplementary Note 12: Sunda-Java

Interpretation. The Sunda-Java SZI event might have re-started subduction at the southern margin of Sundaland (Supplementary Figure 33) with the Indo-Australian plate sinking below the Eurasian plate at around 60–40 Ma (50 Ma is taken as the approximate average of the below timings). This subduction zone eventually evolved into the presently active Sunda-Java subduction system[95,96,97].

It is generally agreed that the earlier accretion of the Woyla arc to Sundaland was followed by a hiatus in subduction along the margin. However, the absolute timings are debated. Hall[96] initiate subduction along Sundaland (Sunda-Java described here) at 45 Ma (hiatus between 90–45 Ma) whereas the reconstruction of Zahirovic et al.[97] suggest only a 10 Myr long hiatus with subduction initiating after 62 Ma (hiatus ~75–62 Ma). Nonetheless, the SZI event might have restarted subduction along the temporarily distinct destructive boundary by an episodic SZI mechanism. The general northward motion of the Indo-Australian plate driven by the surrounding northward directed subduction zones might have induced significant North-South directed compression and thereby fostered the new subduction zone. It is, however, also possible that the subduction zone re-initiated by a lateral progression of still active surrounding subduction systems.

Direct evidence. One age constraint suggesting already ongoing subduction comes from the 42.7 Ma SHRIMP U-Pb date on volcanoclastic zircons[98]. In favour of the slightly earlier SZI timings, a magmatic gap of arc volcanics related to Woyla accretion on Sumatra from ~75–62 Ma[99] was implemented into the reconstruction of Zahirovic et al.[97].

Reconstruction. In the model of Müller et al.[7], the Sunda-Java SZI event ostensibly occurs at 65 Ma, with the initiation of northeast-dipping subduction of the India Plate beneath Eurasia (Supplementary Figure 34). However, that SZI event effectively occurs in name only, as it does not appear to be kinematically distinguishable. Starting in Jurassic time, a 'Sunda' subduction zone was operating along the southwest margin of the Woyla terranes (where the younger Sunda-Java subduction zone formed), and this subduction was already consuming Indian plate oceanic lithosphere by Early Cretaceous time. In the model, it is indicated that this 'Sunda' subduction zone ceased at 75 Ma, and the boundary became an orogenic belt, named 'Woyla accretion', from 75–65 Ma. At 65 Ma, subduction re-initiates as the Sunda-Java subduction zone along that orogenic boundary. According to those evolving boundary descriptions, subduction ceased along that margin for ~10 Ma. However, there is no clear change in the relative convergence rates of the India and Eurasia plates during the ~10 Myr that subduction is supposed to have ceased; and in that 10 Myr interval, >1000 km of relative convergence between those plates occurred along the 'Woyla accretion' boundary. In addition to that possible continuation of subduction along the Sunda-Java subduction zone proper, immediately to the southeast of the Sunda-Java subduction zone lies the Kalimantan subduction zone which was also active prior to 65 Ma.

Seismic tomography. An SZI event along the South of Sundaland at around 60 Ma would place the Sunda slab at about the same depth as it is currently observed in P- and S-wave seismic tomography models[97]. The Atlas of Underworld catalogues an anomaly until 1100 km depth[8]. However, it is stated that the slab is at variable depths along strike of the trench. The western section of the anomaly reaches the bottom of the upper mantle and is clearly separated from the underlying lower mantle anomaly. However, to the east the two anomalies overlap. Based on the vote map, we see an agreement of the models imaging a fast anomaly until 1450–1500 km depth (see Supplementary Figure 35). It is worth noting, though, that the thickness of the slab based on the vote map is over 700 km, which suggests that it could be two overlapping anomalies.

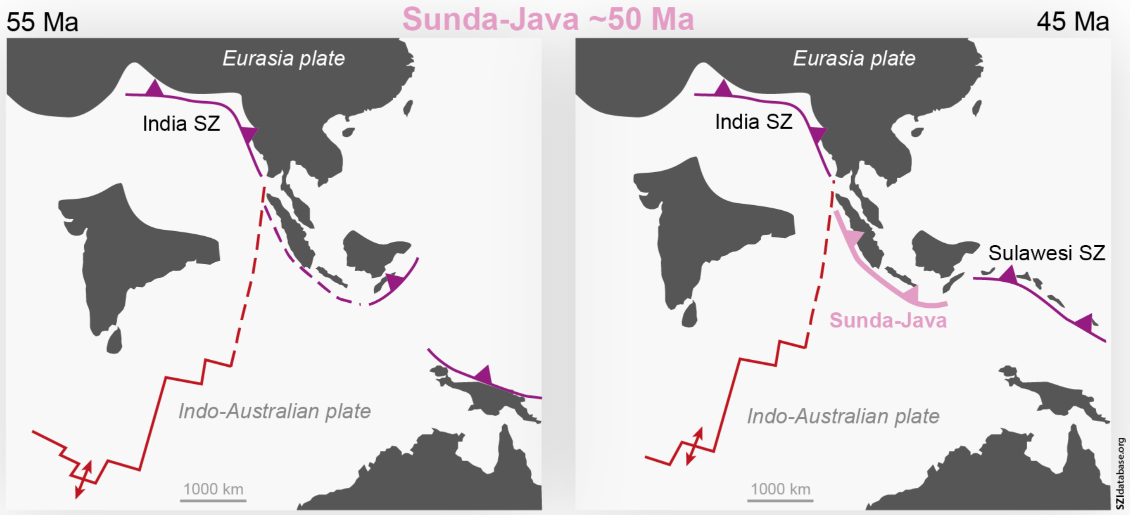

Supplementary Figure 33 Schematic tectonic reconstruction of the Sunda-Java SZI event modified from ref.[96]. Subduction of the Indo-Australian plate at the Sundaland margin has been episodically ongoing since >100 Ma. The most recent episode occurred at ca. 40–60 Ma and it initiated the new Sunda-Java subduction zone. Shown are the new subduction zone (pink line), other active (solid purple lines) and inactive (dashed purple lines) subduction zones, spreading ridges (solid red lines), and transform faults (red dashed lines).

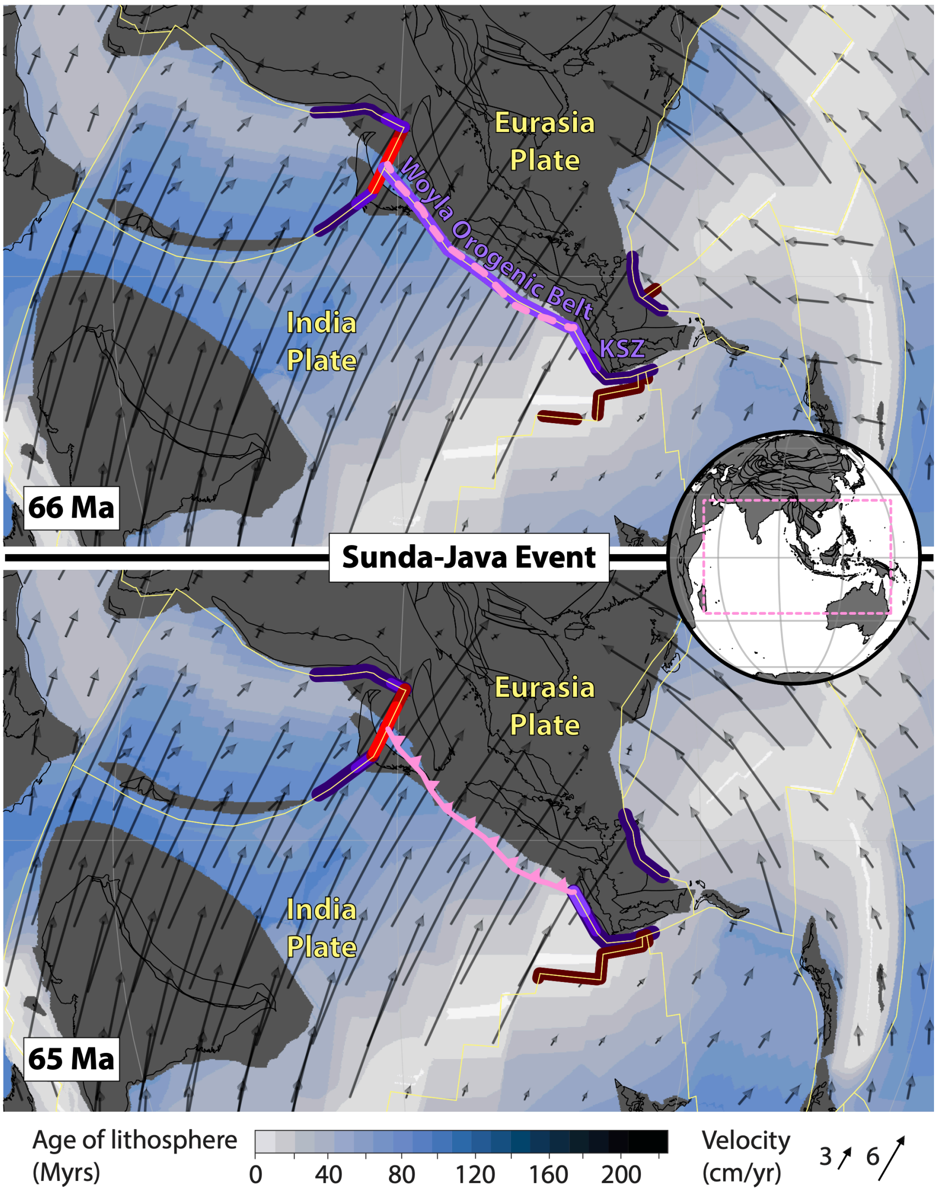

Supplementary Figure 34 Sunda-Java SZI event as reconstructed in the model of Müller et al.[7]. Pink dashed (solid with teeth) line shows the Sunda-Java trench 1 Myr before (at) SZI time in the model. Purple (red) lines show segments of neighbouring subduction zones (ridges and transforms) that lie within some radius of the Izu-Bonin-Mariana trench (pink line); the brightness of the colours reflects 3 different distance thresholds of 250, 500 and 1000 km. KSZ: Kalimantan subduction zone.

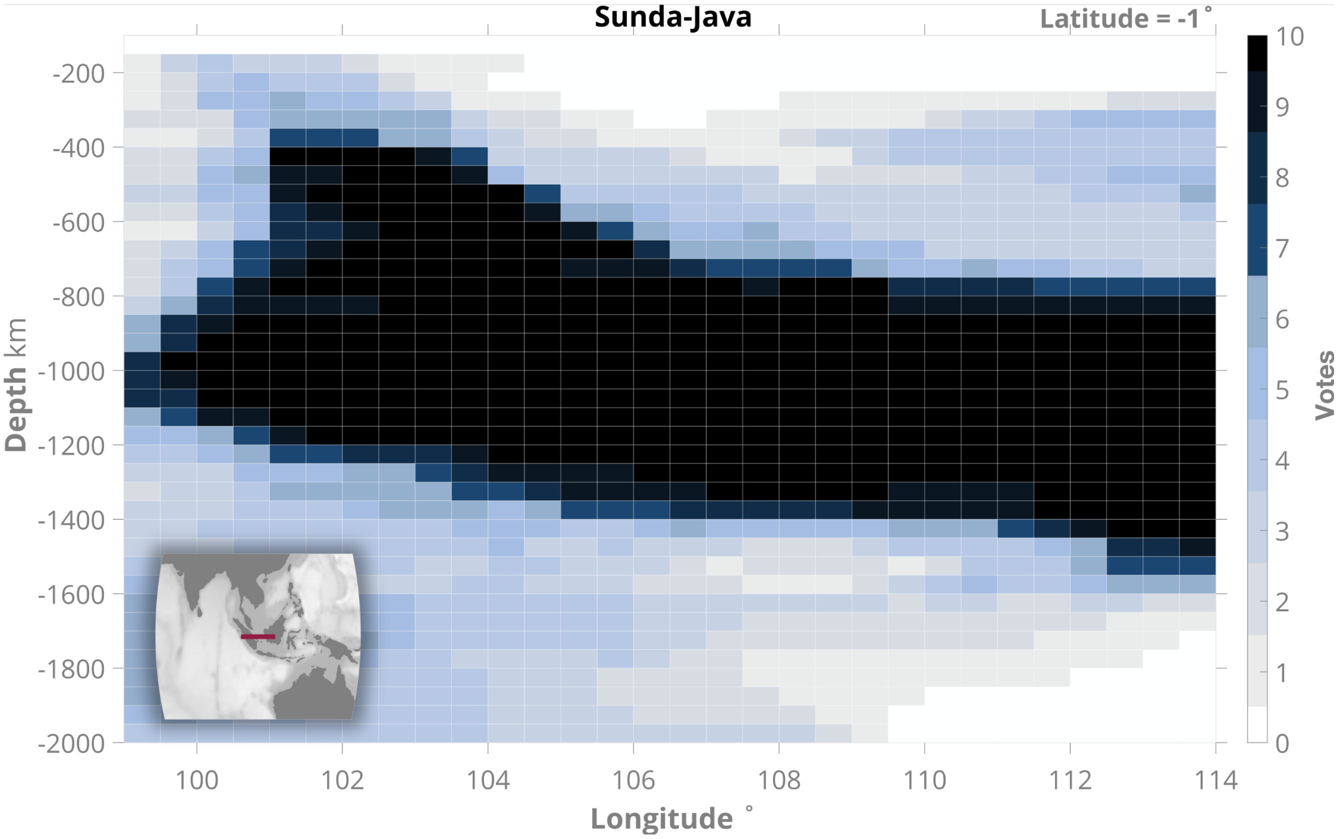

Supplementary Figure 35 Seismic tomography VoteMap[9] analysis of the Sunda-Java SZI event.

Supplementary Note 13: Tonga-Kermadec

Interpretation. The onset of the present-day Tonga-Kermadec subduction zone (Supplementary Figure 36) occurred at around 50 Ma with the subduction of the Pacific plate under the Australian plate (e.g.,[100,101,102]). However, the date of this SZI event is highly debated, with some models suggesting that subduction was active since ~100 Ma[51] and others that it started at ~30 Ma[103].

Most models suggest that the Tonga-Kermadec subduction zone initiated due to the collision of the Papuan peninsula with the trench of the New Caledonia subduction zone (NE dipping subduction) at around 55 Ma (e.g.,[101]). This collision jammed subduction locally and caused a polarity reversal that started the Tonga-Kermadec subduction zone in the north, which progressively propagated southward (e.g.,[100,101,102]). It is also suggested that W-dipping subduction was previously active in the same region (85–65 Ma) and that collision reactivated the fossil subduction zone[101]. In this case, the event could be considered as 'episodic subduction', but it is here preferred to use 'polarity reversal' as it is the main driving mechanism.

Direct evidence. Meffre et al.[102] dated forearc basalts to 52–49 Ma. However, they interpret these basalts as the product of back-arc magmatism of the east-dipping Loyalty-Three Kings subduction zone and thus are not related to the Tonga-Kermadec SZI event. *Boninites* are still forming today in the Tonga-Kermadec subduction zone (e.g.,[104]) and are thus not necessarily related to SZI. The oldest arc ages are ~44 Ma, found in rhyolitic lavas in the south part of the Tonga ridge basement[105]. Similar ages are also observed in different arc rocks in 'Eua Island[106].

Reconstruction. In the model of Müller et al.[7], the Tonga-Kermadec SZI event occurs at 44 Ma (see Supplementary Figure 37). The initiation of the Tonga-Kermadec subduction zone coincides with the cessation of a sub-parallel subduction zone ('South Loyalty Basin subduction zone') that lies between 0 and ~800 km to the southwest of the Tonga subduction zone (and passes through the trace of the future Kermadec subduction zone at a high angle). The location of the Tonga-Kermadec subduction zone furthermore appears to approximately trace an older subduction zone along the northeast side of the Proto-Loyalty Arc plate, which was active until 56 Ma. The initiation of the Tonga-Kermadec subduction zone was associated with the formation of a new plate, the North Loyalty plate, and shortly before the initiation of the Tonga-Kermadec subduction zone at 44 Ma, there was a major change in the motion and heading of both the Pacific and Australian plates at 47 Ma.

Seismic tomography. The Tonga-Kermadec slab is represented by a fast seismic anomaly reaching to a depth of 1500–1600 km[8], but P- and S-wave tomography models are consistent only until 1400–1550 km in the vote map (7–6 out of 10, respectively; see Supplementary Figure 38). The anomaly is quite homogeneous along the entire Tonga-Kerdamec trench, although the slab penetrates more vertically to the transition zone to the South[8].

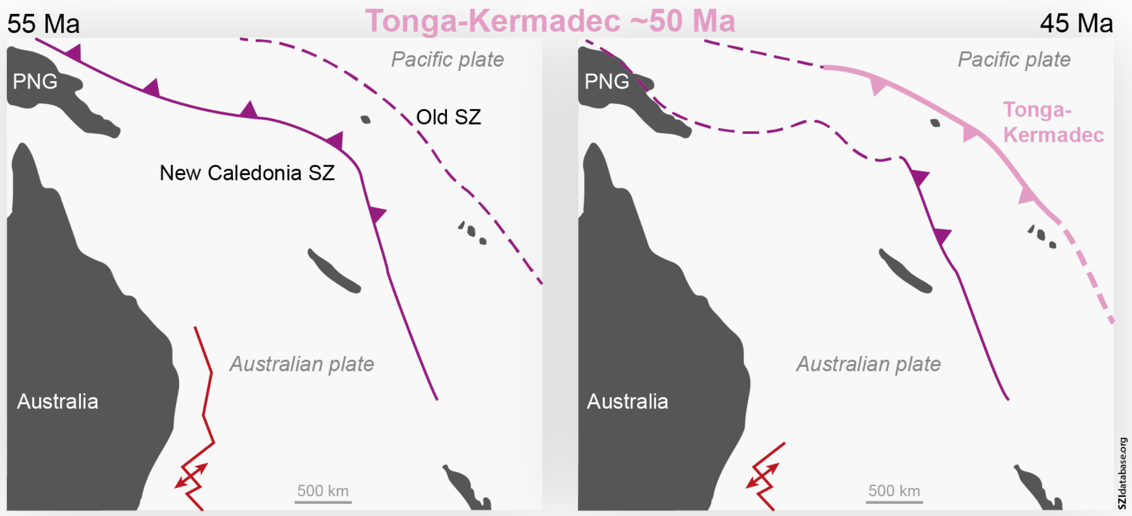

Supplementary Figure 36 Schematic tectonic reconstruction of the Tonga-Kermadec SZI event modified from ref.[101]. The collision of the Papua New Guinea continental block with the Loyalty-Three Kings trench is suggested to have caused a flip in subduction polarity, initiating the new Tonga-Kermadec subduction zone, possibly exploiting a weakness due to the presence of an old subduction zone. Shown are the new subduction zone (pink line), other active (solid purple lines) and inactive (dashed purple lines) subduction zones, and spreading ridges (solid red lines).

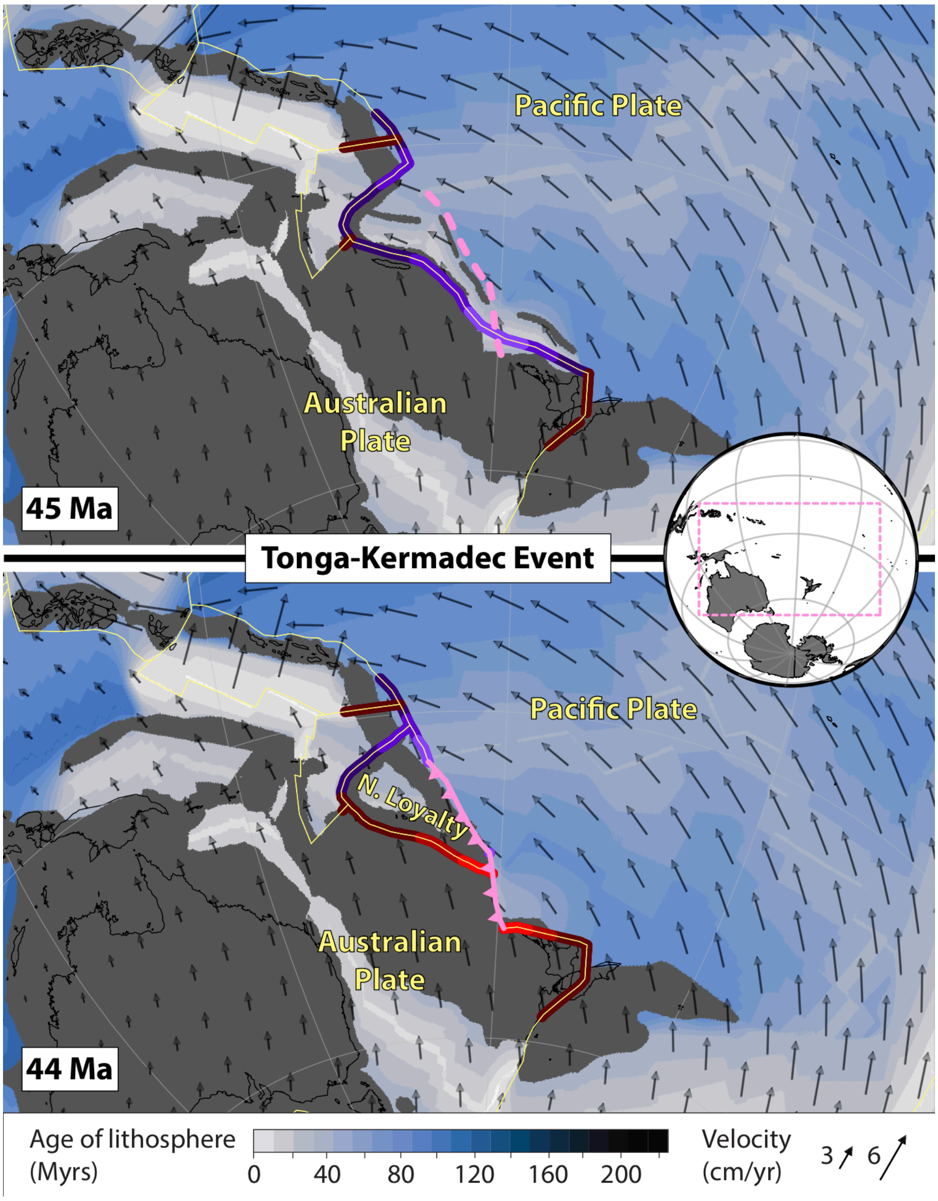

Supplementary Figure 37 Tonga-Kermadec SZI event as reconstructed in the model of Müller et al.[7]. Pink dashed (solid with teeth) line shows the Tonga-Kermadec trench 1 Myr before (at) SZI time in the model. Purple (red) lines show segments of neighbouring subduction zones (ridges and transforms) that lie within some radius of the Tonga-Kermadec trench (pink line); the brightness of the colours reflects 3 different distance thresholds of 250, 500 and 1000 km.

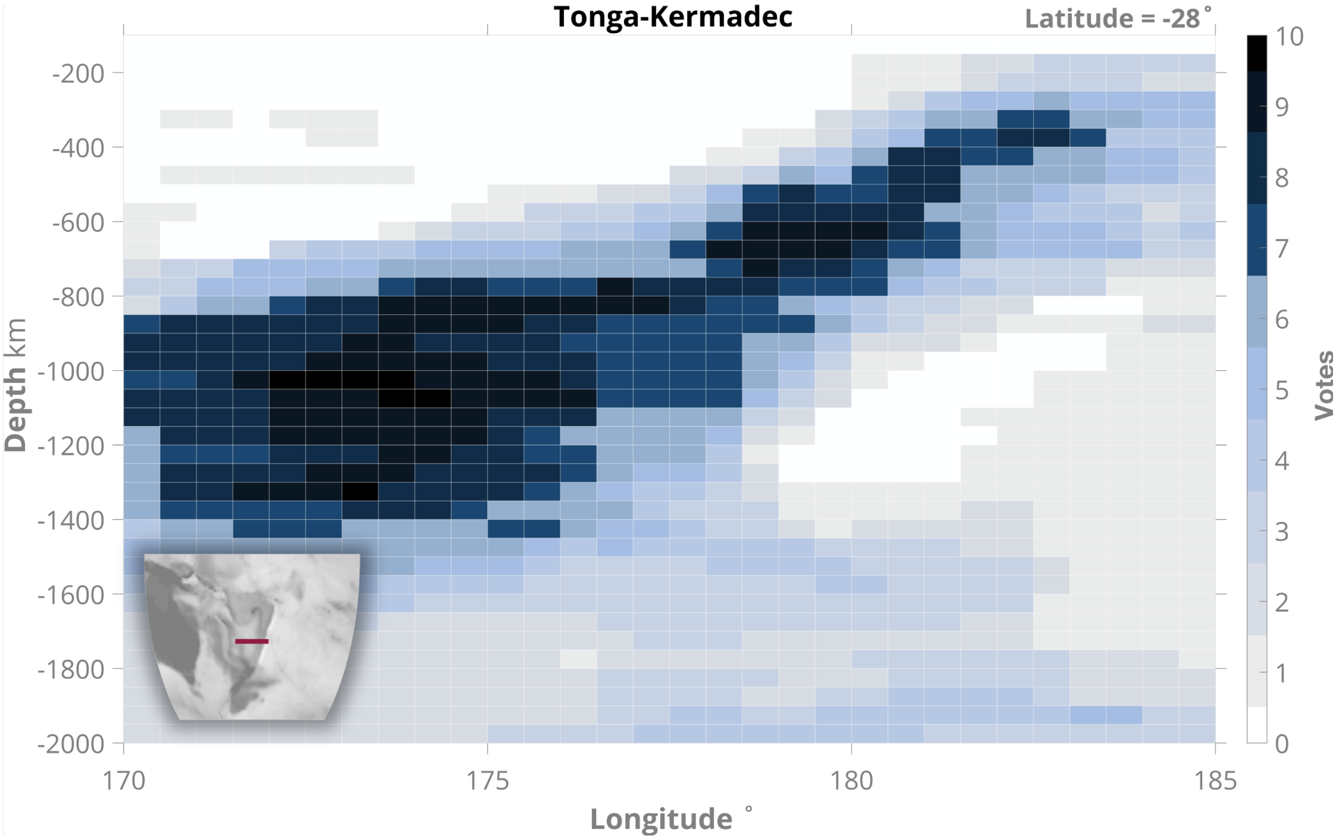

Supplementary Figure 38 Seismic tomography VoteMap[9] analysis of the Tonga-Kermadec SZI event.

Supplementary Note 14: Unclear SZI events

"Unclear SZI events" are events that might be classified as SZI, but currently lack key information or are described by multiple research groups with strongly differing, incompatible interpretations. These events in particular should be revisited in future studies.

Manila (unclear nature). Strongly different reconstructions are proposed for the onset of the present-day Manila (a.k.a. Luzon) subduction zone. For instance, the reconstruction by Wu et al.[36] suggests that the Manila subduction initiated at 20 Ma at a transform fault with the Eurasian-South China Sea subducting below the Philippine Sea plate. Other models (e.g.,[107]) propose a flip in subduction polarity at 15 Ma from a west-dipping subduction of the Philippine Sea plate below Eurasia to an east-dipping subduction that started the Luzon arc in an intra-oceanic setting.

The reconstructions from the literature strongly differ from the current plate reconstruction model[7] as supplied with GPlates. The GPlates reconstruction currently also misrepresents the polarity of the present-day Manila subduction zone, which is East-dipping as seen via slab earthquakes and through seismic tomography (e.g.,[36]). We decided to not include the Manila SZI event in the database because of the significant discrepancies among the geodynamic reconstruction models proposed in the literature.

Mid America (unclear nature and timing). The Mid America SZI event was related to the subduction of the Farallon plate (later Cocos plate) below the Caribbean plate[45] in the region between present-day Mexico and Costa Rica. Some studies indicate a possible onset of subduction at this location around 85 Ma[45]. This timing would also match evidence of a plate reconfiguration based on the adjacent Caribbean subduction history – at the eastern margin of the Caribbean plate, the Aves ridge, a relict island arc was active from 88 to 59 Ma[49]. Furthermore, Buchs et al.[108] suggest the arc in southern Costa Rica and Western Panama started forming at 75–73 Ma on top of an oceanic plateau. Galli-Olivier[109] suggested the island arc suite in Costa Rica started in the early Campanian (about 83 Ma), having found clastic rocks unconformably covered an ophiolite that is older (i.e., 150–83 Ma). This ophiolite is suggested to be composed of oceanic crust formed at a ridge, thus, it is not a SZI ophiolite (or supra-subduciton zone ophiolite). Therefore, it cannot be used to constrain the timing of the SZI event.

It is also worth noting that long-lived subduction along the broader eastern margin of Panthalassa since at least 200 Ma is predicted based on global plate reconstructions (involving the Farallon, Cocos plates), rendering the timing and mechanism for the Mid America SZI difficult to ascertain.

Looking into the mantle record, the Cocos slab is the longest slab identified in the Atlas of the Underworld database[8]. Based on a reinterpretation in[110], the Atlas associates this slab to the Mid America subduction zone, and suggested continuous subduction since around 230 Ma. It is, however, difficult, if not impossible, to distinguish present-day slabs as separate subduction zones as they are in close proximity to each other.

Due to the uncertainty in the onset timing and nature of subduction, including with respect to contemporaneous broader Panthalassa and Caribbean subduction, we decided not to include this event in the database. We suggest that it is a target for the next iteration of the database.

Papua-New Guinea (unclear nature). The Papua-New Guinea (PNG) SZI event would be related to convergence between the Australian and Caroline plates. The PNG/New Guinea trench at present-day exhibits southward subduction of the Caroline plate, and is thought to have initiated in the late Eocene-Oligocene at around 40 Ma[111]. Towards the east, the present-day plate boundary continues to the Bismarck and Solomon seas, where subduction polarity is of the opposite sense.

There are many competing scenarios for the Cenozoic evolution of New Guinea (e.g.,[112]), owing to an uncertain chronology of major tectonic events prior to the initiation of the presently active trench, as well as poorly-constrained subduction polarities. In some models, the PNG subduction starts as a polarity flip from north- to south-dipping subduction at ages that range from 20 to 5 Ma (e.g.,[112,113]). Other scenarios consider the PNG trench as a propagation of the Melanasian trench, which has been active since at least 40 Ma[114]. We decided to not include the Papua SZI event in the database because of the significant discrepancies among the geodynamic reconstruction models proposed in the literature.

South America (older than 100 Ma). Subduction of the Nazca and Farallon (for earlier times) plates below the eastern continental margin South America has been active since at least 140 Ma, if not earlier[115,116,117]. Thus, we do not include the South America SZI event in this version of the database, which is currently intended to cover only SZI events back to around 100 Ma.

Kamchatka-Japan (older than 100 Ma and/or unclear nature and timing). Subduction of the Pacific plate, and Izanagi plate for earlier times, dominated the tectonics in the northwest region of the Panthalassa margin since at least 200 Ma. A discrete subduction episode within the last 100 Myr may have initiated due to ridge subduction[118] or the accretion of arc complexes and subduction reorganisation around the Hawaiian-Emperor Bend time[4]. However, the current uncertainty in the nature and timing renders a discrete SZI entry to be beyond

the scope of the current version of the database and is a target for future efforts.

Supplementary Note 15: Non-SZI events

The **Puysegur "Trench"** (also referred to as being located within the Puysegur-Fiordland system) is a region located south of New Zealand. It is proposed to be the site of incipient subduction[119] due to the geophysical and seismic imaging of the megathrust interface and recent plate motions. Since a self-sustaining subduction has not formed here yet, strictly speaking, it cannot be considered a SZI event. Therefore, we decided to not include it in the database.

The **Yap subduction zone** is located towards the south of the Izu-Bonin-Mariana (IBM) subduction zone. It is not clear, if the Yap subduction zone is active at present-day and whether the trench represents a new subduction zone, or if it is the continuation of the Marianas trench. Below is a discussion that led us to conclude that we should not consider it as a new SZI event.

Most reconstructions suggest that the Yap trench was originally part of the Mariana subduction zone, which at about 25 Ma collided with the Caroline ridge. This event lead to a local slowing down and eventual cessation of subduction. At about the same time, the Parece Vela basin started spreading and the Mariana trench in the north continued to retreat, leaving behind the Yap segment. Some studies (e.g.,[41]) consider the Yap subduction to have been inactive since then. However, there are some rocks with arc affinity that were dredged in the Yap forearc that have been dated with K/Ar at 11–7 Ma[120]. These rocks seem to be the only record of recent activity of the Yap arc after the Caroline ridge collision. Because they were dated with K/Ar, their provenance is uncertain since they were dredged, and their affinity might also agree with back-arc magmatism[121], we do not think this data is enough to prove subduction was active at 11–7 Ma. Therefore, if subduction has stopped after the Caroline ridge collision (at 25 Ma), the Yap trench cannot be considered a location of a SZI event.

Today, it is unclear if subduction is active in the region. Zhang et al.[122] suggest that subduction is active today, with a very slow convergence rate of 3–6 mm/yr. However, there is no deep seismicity, no slab, and no active volcanism in the region. The high heat flux, the gravity anomalies and the shallow seismicity, can all be possibly explained with a shallow remnant slab from when subduction was active as part of the Marianas subduction zone. Nevertheless, even if we think that subduction has stopped after the Caroline ridge collision and then started again (with maybe some arc activity at 11–7 Ma), it is hard to imagine that the slab was deep enough at 11 Ma to generate arc volcanism and, then, did not subduct any further (otherwise a slab would be clearly visible). Moreover, if subduction is active today, the subducting plate is the Caroline ridge, which would be hard to subduct, as it is not negatively buoyant with respect to the underlying mantle. Therefore, it is not possible to consider it as a SZI event, since no self-sustained subduction has formed (neither at 11 Ma, nor now).

To conclude, regardless of which reconstruction we take into account, we arrive at the same conclusion that this is not a (new or discrete) SZI event.

Supplementary References

1. Davis, A. S., L.-B. G. Pickthorn, T. L. Valuer, and M. S. Marlow. Petrology and age of volcanic-arc rocks from the continental margin of the bering sea: implications for early eocene relocation of plate boundaries. *Canadian Journal of Earth Sciences*, 26(7): 1474–1490, 1989.
2. Jicha, B. R., D. W. Scholl, B. S. Singer, G. M. Yogodzinski, and S. M. Kay. Revised age of aleutian island arc formation implies high rate of magma production. *Geology*, 34(8):661–664, 2006.
3. Scholl, D. W. Viewing the tectonic evolution of the kamchatka-aleutian (kat) connection with an alaska crustal extrusion perspective. *Washington DC American Geophysical Union Geophysical Monograph Series*, 172:3–35, 2007.
4. Domeier, M., G. E. Shephard, J. Jakob, C. Gaina, P. V. Doubrovine, and T. H. Torsvik. Intraoceanic subduction spanned the pacific in the late cretaceous–paleocene. *Science advances*, 3(11):eaao2303, 2017.
5. Vaes, B., D. J. Van Hinsbergen, and L. M. Boschman. Reconstruction of subduction and back-arc spreading in the nw pacific and aleutian basin: Clues to causes of cretaceous and eocene plate reorganizations. *Tectonics*, 38(4):1367–1413, 2019.
6. Minyuk, P. and D. Stone. Paleomagnetic determination of paleolatitude and rotation of bering island (komandorsky islands) russia: Comparison with rotations in the aleutian islands and kamchatka. *Stephan Mueller Special Publication Series*, 4:329–348, 2009.
7. Müller, R. D., M. Seton, S. Zahirovic, S. E. Williams, K. J. Matthews, N. M. Wright, G. E. Shephard, K. T. Maloney, N. Barnett-Moore, M. Hosseinpour, D. J. Bower, and J. Cannon. Ocean basin evolution and global-scale plate reorganization events since pangea breakup. *Annual Review of Earth and Planetary Sciences*, 44(1):107–138, 2016. doi: 10.1146/annurev-earth-060115-012211. URL https://doi.org/10.1146/annurev-earth-060115-012211.
8. van der Meer, D. G., D. J. J. van Hinsbergen, and W. Spakman. Atlas of the underworld: Slab remnants in the mantle, their sinking history, and a new outlook on lower mantle viscosity. *Tectonophysics*, 723:309–448, Jan. 2018. ISSN 0040-1951. URL http://www.sciencedirect.com/science/article/pii/S0040195117304055.
9. Shephard, G. E., K. J. Matthews, K. Hosseini, and M. Domeier. On the consistency of seismically imaged lower mantle slabs. *Scientific Reports*, 7(1):10976, Sept. 2017. ISSN 2045-2322. doi: 10.1038/s41598-017-11039-w. URL https://doi.org/10.1038/s41598-017-11039-w.
10. van Hinsbergen, D. J., M. Maffione, L. M. Koornneef, and C. Guilmette. Kinematic and paleomagnetic restoration of the semail ophiolite (oman) reveals subduction initiation along an ancient neotethyan fracture zone. *Earth and Planetary Science Letters*, 518:183–196, 2019.
11. Agard, P., L. Jolivet, B. Vrielynck, E. Burov, and P. Monie. Plate acceleration: the obduction trigger? *Earth and Planetary Science Letters*, 258(3-4):428–441, 2007.
12. Gürer, D., D. J. van Hinsbergen, L. Matenco, F. Corfu, and A. Cascella. Kinematics of a former oceanic plate of the neotethys revealed by deformation in the ulukışla basin (turkey). *Tectonics*, 35(10):2385–2416, 2016.
13. Gaina, C., D. J. Van Hinsbergen, and W. Spakman. Tectonic interactions between india and arabia since the jurassic reconstructed from marine geophysics, ophiolite geology, and seismic tomography. *Tectonics*, 34(5):875–906, 2015.
14. van Hinsbergen, D. J., T. H. Torsvik, S. M. Schmid, L. C. Maţenco, M. Maffione, R. L. Vissers, D. Gürer, and W. Spakman. Orogenic architecture of the mediterranean region and kinematic reconstruction of its tectonic evolution since the triassic. *Gondwana Research*, 2019.
15. Biryol, C. B., S. L. Beck, G. Zandt, and A. A. Özacar. Segmented african lithosphere beneath the anatolian region inferred from teleseismic p-wave tomography. *Geophysical Journal International*, 184(3):1037–1057, 2011.
16. Portner, D. E., J. R. Delph, C. B. Biryol, S. L. Beck, G. Zandt, A. A. Özacar, E. Sandvol, and N. Türkelli. Subduction termination through progressive slab deformation across Eastern Mediterranean subduction zones from updated P-wave tomography beneath Anatolia. *Geosphere*, 14(3):907–925, 05 2018. ISSN 1553-040X. doi: 10.1130/GES01617.1. URL https://doi.org/10.1130/GES01617.1.
17. Peters, K., M. Smit, D. van Hinsbergen, H. van Roermund, and F. Brouwer. The longevity of neotethyan metamorphic soles from lu-hf garnet chronology. In *EGU General Assembly Conference Abstracts*, volume 19, page 15673, 2017.
18. Pourteau, A., E. E. Scherer, S. Schorn, R. Bast, A. Schmidt, and L. Ebert. Thermal evolution of an ancient subduction interface revealed by lu–hf garnet geochronology, halilbağı complex (anatolia). *Geoscience Frontiers*, 10(1):127–148, 2019.
19. van Hinsbergen, D. J., M. Maffione, A. Plunder, N. Kaymakcı, M. Ganerød, B. W. Hendriks, F. Corfu, D. Gürer, G. I. de Gelder, K. Peters, et al. Tectonic evolution and paleogeography of the kırşehir block and the central anatolian ophiolites, turkey. *Tectonics*, 35(4):983–1014, 2016.
20. Guilmette, C., M. A. Smit, D. J. van Hinsbergen, D. Gürer, F. Corfu, B. Charette, M. Maffione, O. Rabeau, and D. Savard. Forced subduction initiation recorded in the sole and crust of the semail ophiolite of oman. *Nature Geoscience*, 11(9):688, 2018.
21. Whitney, D. L., C. Teyssier, A. K. Fayon, M. A. Hamilton, and M. Heizler. Tectonic controls on metamorphism, partial melting, and intrusion: timing and duration of regional metamorphism and magmatism in the niğde massif, turkey. *Tectonophysics*, 376 (1-2):37–60, 2003.
22. Lefebvre, C., M. J. Meijers, N. Kaymakci, A. Peynircioğlu, C. G. Langereis, and D. J. Van Hinsbergen. Reconstructing the geometry of central anatolia during the late cretaceous: Large-scale cenozoic rotations and deformation between the pontides and taurides. *Earth and Planetary Science Letters*, 366:83–98, 2013.
23. Hyndman, R., C. Yorath, R. Clowes, and E. Davis. The northern cascadia subduction zone at vancouver island: Seismic structure and tectonic history. *Canadian Journal of Earth Sciences*, 27(3):313–329, 1990.
24. Priest, G. R. Volcanic and tectonic evolution of the cascade volcanic arc, central oregon. *Journal of Geophysical Research: Solid Earth*, 95(B12):19583–19599, 1990.
25. Schmandt, B. and E. Humphreys. Seismically imaged relict slab from the 55 ma siletzia accretion to the northwest united states. *Geology*, 39(2):175–178, 2011.
26. Stern, R. J. and T. A. Dumitru. Eocene initiation of the cascadia subduction zone: A second example of plume-induced subduction initiation? *Geosphere*, 15(3):659–681, 2019.
27. Wells, R., D. Bukry, R. Friedman, D. Pyle, R. Duncan, P. Haeussler, and J. Wooden. Geologic history of siletzia, a large igneous province in the oregon and washington coast range: Correlation to the geomagnetic polarity time scale and implications for a long-lived yellowstone hotspot. *Geosphere*, 10(4):692–719, 2014.
28. Eddy, M. P., K. P. Clark, and M. Polenz. Age and volcanic stratigraphy of the Eocene Siletzia oceanic plateau in Washington and on Vancouver Island. *Lithosphere*, 9(4):652–664, 05 2017. ISSN 1941-8264. doi: 10.1130/L650.1. URL https://doi.org/10.1130/L650.1.
29. du Bray, E. A. and D. A. John. Petrologic, tectonic, and metallogenic evolution of the ancestral cascades magmatic arc, washington, oregon, and northern california. *Geosphere*, 7(5):1102–1133, 2011.
30. Baker, S. and J. Malaihollo. Dating of neogene igneous rocks in the halmahera region: arc initiation and development. *Geological Society, London, Special Publications*, 106(1):499–509, 1996.
31. Hall, R. Reconstructing cenozoic se asia. *Geological Society, London, Special Publications*, 106(1):153–184, 1996.

32. Hall, R. and W. Spakman. Mantle structure and tectonic history of se asia. *Tectonophysics*, 658:14 – 45, 2015. ISSN 0040-1951. doi: https://doi.org/10.1016/j.tecto.2015.07.003. URL http://www.sciencedirect.com/science/article/pii/S0040195115003698.
33. Chandra, J. and R. Hall. Tectono-stratigraphic evolution and hydrocarbon prospectivity of the south halmahera basin, indonesia. *The Indonesian Petroleum Association's 40th Annual Convention & Exhibition Proceedings*, IPA16-46-G, 2016.
34. Zhang, Q., F. Guo, L. Zhao, and Y. Wu. Geodynamics of divergent double subduction: 3-d numerical modeling of a cenozoic example in the molucca sea region, indonesia. *Journal of Geophysical Research: Solid Earth*, 122(5):3977–3998, 2017.
35. Hall, R. and H. R. Smyth. Cenozoic arc processes in indonesia: Identification of the key influences on the stratigraphic record in active volcanic arcs. *Formation and applications of the sedimentary record in arc collision zones*, 436:27, 2008.
36. Wu, J., J. Suppe, R. Lu, and R. Kanda. Philippine sea and east asian plate tectonics since 52 ma constrained by new subducted slab reconstruction methods. *Journal of Geophysical Research: Solid Earth*, 121(6):4670–4741, 2016.
37. Hall, R., M. G. Audley-Charles, F. T. Banner, S. Hidayat, and S. L. Tobing. Basement rocks of the halmahera region, eastern indonesia: a late cretaceous–early tertiary arc and fore-arc. *Journal of the Geological Society*, 145(1):65–84, 1988. ISSN 0016-7649. doi: 10.1144/gsjgs.145.1.0065. URL https://jgs.lyellcollection.org/content/145/1/65.
38. Ishizuka, O., R. Hickey-Vargas, R. J. Arculus, G. M. Yogodzinski, I. P. Savov, Y. Kusano, A. McCarthy, P. A. Brandl, and M. Sudo. Age of izu–bonin–mariana arc basement. *Earth and Planetary Science Letters*, 481:80–90, 2018.
39. Reagan, M. K., D. E. Heaton, M. D. Schmitz, J. A. Pearce, J. W. Shervais, and A. A. Koppers. Forearc ages reveal extensive short-lived and rapid seafloor spreading following subduction initiation. *Earth and Planetary Science Letters*, 506:520–529, 2019.
40. O'Connor, J. M., B. Steinberger, M. Regelous, A. A. Koppers, J. R. Wijbrans, K. M. Haase, P. Stoffers, W. Jokat, and D. Garbe-Schönberg. Constraints on past plate and mantle motion from new ages for the hawaiian-emperor seamount chain. *Geochemistry, Geophysics, Geosystems*, 14(10):4564–4584, 2013.
41. Lallemand, S. Philippine sea plate inception, evolution, and consumption with special emphasis on the early stages of izu-bonin-mariana subduction. *Progress in Earth and Planetary Science*, 3(1):15, 2016.
42. Hall, C. E., M. Gurnis, M. Sdrolias, L. L. Lavier, and R. Müller. Catastrophic initiation of subduction following forced convergence across fracture zones. *Earth and Planetary Science Letters*, 212(1–2):15–30, July 2003. ISSN 0012-821X. URL http://www.sciencedirect.com/science/article/pii/S0012821X03002425.
43. Ishizuka, O., R. N. Taylor, Y. Ohara, and M. Yuasa. Upwelling, rifting, and age-progressive magmatism from the oki-daito mantle plume. *Geology*, 41(9):1011–1014, 2013.
44. Ishizuka, O., K. Tani, M. K. Reagan, K. Kanayama, S. Umino, Y. Harigane, I. Sakamoto, Y. Miyajima, M. Yuasa, and D. J. Dunkley. The timescales of subduction initiation and subsequent evolution of an oceanic island arc. *Earth and Planetary Science Letters*, 306(3-4):229–240, 2011.
45. Boschman, L. M., D. J. van Hinsbergen, T. H. Torsvik, W. Spakman, and J. L. Pindell. Kinematic reconstruction of the caribbean region since the early jurassic. *Earth-Science Reviews*, 138:102–136, 2014.
46. van Benthem, S., R. Govers, W. Spakman, and R. Wortel. Tectonic evolution and mantle structure of the caribbean. *Journal of Geophysical Research: Solid Earth*, 118(6):3019–3036, 2013. doi: 10.1002/jgrb.50235. URL https://agupubs.onlinelibrary.wiley.com/doi/abs/10.1002/jgrb.50235.
47. Aitken, T., P. Mann, A. Escalona, and G. L. Christeson. Evolution of the grenada and tobago basins and implications for arc migration. *Marine and Petroleum Geology*, 28(1):235 – 258, 2011. ISSN 0264-8172. doi: https://doi.org/10.1016/j.marpetgeo.2009.10.003. URL http://www.sciencedirect.com/science/article/pii/S0264817209001755. Thematic Set on: Tectonics, basinal framework, and petroleum systems of eastern Venezuela, the Leeward Antilles, Trinidad and Tobago, and offshore areas.
48. Briden, J., D. Rex, A. Faller, and J. Tomblin. K-ar geochronology and palaeomagnetism of volcanic rocks in the lesser antilles island arc. *Philosophical Transactions of the Royal Society of London. Series A, Mathematical and Physical Sciences*, 291(1383): 485–528, 1979.
49. Neill, I., A. C. Kerr, A. R. Hastie, K.-P. Stanek, and I. L. Millar. Origin of the aves ridge and dutch–venezuelan antilles: interaction of the cretaceous 'great arc'and caribbean–colombian oceanic plateau? *Journal of the Geological Society*, 168(2):333–348, 2011.
50. Speed, R. C. and D. K. Larue. Barbados: Architecture and implications for accretion. *Journal of Geophysical Research: Solid Earth*, 87(B5):3633–3643, 1982. doi: 10.1029/JB087iB05p03633. URL https://agupubs.onlinelibrary.wiley.com/doi/abs/10.1029/JB087iB05p03633.
51. Schellart, W., G. Lister, and V. Toy. A late cretaceous and cenozoic reconstruction of the southwest pacific region: tectonics controlled by subduction and slab rollback processes. *Earth-Science Reviews*, 76(3-4):191–233, 2006.
52. Chase, C. G. Tectonic history of the fiji plateau. *Geological Society of America Bulletin*, 82(11):3087–3110, 1971.
53. Auzende, J.-M., Y. Lafoy, and B. Marsset. Recent geodynamic evolution of the north fiji basin (southwest pacific). *Geology*, 16 (10):925–929, 1988.
54. Greene, H. G., J.-Y. Collot, M. A. Fisher, and A. J. Crawford. Neogene tectonic evolution of the new hebrides island arc: A review incorporating odp drilling results. In *Proceedings of the Ocean Drilling Program, Scientific Results*, volume 134, pages 19–46. Ocean Drilling Program College Station, TX, 1994.
55. Holm, R. J., C. Spandler, and S. W. Richards. Melanesian arc far-field response to collision of the ontong java plateau: geochronology and petrogenesis of the simuku igneous complex, new britain, papua new guinea. *Tectonophysics*, 603:189–212, 2013.
56. Monjaret, M., H. Bellon, and P. Maillet. Magmatism of the troughs behind the new hebrides island arc (rv jean charcot seapso 2 cruise): K-ar geochronology and petrology. *Journal of Volcanology and Geothermal Research*, 46(3-4):265–280, 1991.
57. Falvey, D. A. Analysis of palaeomagnetic data from the new hebrides. *Exploration Geophysics*, 9(3):117–123, 1978. URL https://doi.org/10.1071/EG978117.
58. Musgrave, R. J. and J. V. Firth. Magnitude and timing of new hebrides arc rotation: Paleomagnetic evidence from nendo, solomon islands. *Journal of Geophysical Research: Solid Earth*, 104(B2):2841–2853, 1999. doi: 10.1029/1998JB900080. URL https://agupubs.onlinelibrary.wiley.com/doi/abs/10.1029/1998JB900080.
59. Pelletier, B., Y. Lafoy, and F. Missegue. Morphostructure and magnetic fabric of the northwestern north fiji basin. *Geophysical Research Letters*, 20(12):1151–1154, 1993. doi: 10.1029/93GL01240. URL https://agupubs.onlinelibrary.wiley.com/doi/abs/10.1029/93GL01240.
60. Holm, R. J., G. Rosenbaum, and S. W. Richards. Post 8 ma reconstruction of papua new guinea and solomon islands: Microplate tectonics in a convergent plate boundary setting. *Earth-Science Reviews*, 156:66–81, 2016.
61. Boudier, F., G. Ceuleneer, and A. Nicolas. Shear zones, thrusts and related magmatism in the oman ophiolite: initiation of thrusting on an oceanic ridge. *Tectonophysics*, 151(1-4):275–296, 1988.
62. Nicolas, A., F. Boudier, B. Ildefonse, and E. Ball. Accretion of oman and united arab emirates ophiolite–discussion of a new structural map. *Marine Geophysical Researches*, 21(3-4):147–180, 2000.
63. Duretz, T., P. Agard, P. Yamato, C. Ducassou, E. B. Burov, and T. V. Gerya. Thermo-mechanical modeling of the obduction process based on the oman ophiolite case. *Gondwana Research*, 32:1–10, 2016.
64. Maffione, M., D. J. J. van Hinsbergen, G. I. N. O. de Gelder, F. C. van der Goes, and A. Morris. Kinematics of late cretaceous subduction initiation in the neo-tethys ocean reconstructed from ophiolites of turkey, cyprus, and syria. *Journal of Geophysical Research: Solid Earth*, 122(5):3953–3976, 2017. doi: 10.1002/2016JB013821. URL https://agupubs.onlinelibrary.wiley.com/

doi/abs/10.1002/2016JB013821.
65. Koop, W. and R. Stoneley. Subsidence history of the middle east zagros basin, permian to recent. *Philosophical Transactions of the Royal Society of London. Series A, Mathematical and Physical Sciences*, 305(1489):149–168, 1982.
66. Searle, M. and J. Cox. Tectonic setting, origin, and obduction of the oman ophiolite. *Geological Society of America Bulletin*, 111 (1):104–122, 1999.
67. Al-Riyami, K., A. Robertson, J. Dixon, and C. Xenophontos. Origin and emplacement of the late cretaceous baer–bassit ophiolite and its metamorphic sole in nw syria. *Lithos*, 65(1-2):225–260, 2002.
68. Searle, M., C. Warren, D. Waters, and R. Parrish. Structural evolution, metamorphism and restoration of the arabian continental margin, saih hatat region, oman mountains. *Journal of Structural Geology*, 26(3):451–473, 2004.
69. Dilek, Y. and H. Furnes. Structure and geochemistry of tethyan ophiolites and their petrogenesis in subduction rollback systems. *Lithos*, 113(1-2):1–20, 2009.
70. Homke, S., J. Vergés, J. Serra-Kiel, G. Bernaola, I. Sharp, M. Garcés, I. Montero-Verdú, R. Karpuz, and M. H. Goodarzi. Late cretaceous–paleocene formation of the proto–zagros foreland basin, lurestan province, sw iran. *Geological Society of America Bulletin*, 121(7-8):963–978, 2009.
71. Agard, P., J. Omrani, L. Jolivet, H. Whitechurch, B. Vrielynck, W. Spakman, P. Monié, B. Meyer, and R. Wortel. Zagros orogeny: a subduction-dominated process. *Geological Magazine*, 148(5-6):692–725, 2011.
72. Rioux, M., J. Garber, A. Bauer, S. Bowring, M. Searle, P. Kelemen, and B. Hacker. Synchronous formation of the metamorphic sole and igneous crust of the semail ophiolite: New constraints on the tectonic evolution during ophiolite formation from high-precision u–pb zircon geochronology. *Earth and Planetary Science Letters*, 451:185–195, 2016.
73. Kusano, Y., S. Umino, R. Shinjo, A. Ikei, Y. Adachi, S. Miyashita, and S. Arai. Contribution of slab-derived fluid and sedimentary melt in the incipient arc magmas with development of the paleo-arc in the oman ophiolite. *Chemical Geology*, 449:206–225, 2017.
74. Alabaster, T., J. Pearce, and J. Malpas. The volcanic stratigraphy and petrogenesis of the oman ophiolite complex. *Contributions to Mineralogy and Petrology*, 81(3):168–183, 1982.
75. Marchadier, Y. and C. Rangin. Polyphase tectonics at the southern tip of the manila trench, mindoro-tablas islands, philippines. *Tectonophysics*, 183(1-4):273–287, 1990.
76. Yumul Jr, G. P., C. B. Dimalanta, R. A. Tamayo Jr, and R. C. Maury. Collision, subduction and accretion events in the philippines: A synthesis. *Island Arc*, 12(2):77–91, 2003. doi: 10.1046/j.1440-1738.2003.00382.x. URL https://onlinelibrary.wiley.com/doi/abs/10.1046/j.1440-1738.2003.00382.x.
77. Barrier, E., P. Huchon, and M. Aurelio. Philippine fault: a key for philippine kinematics. *Geology*, 19(1):32–35, 1991.
78. Sajona, F. G., H. Bellon, R. C. Maury, M. Pubellier, R. D. Quebral, J. Cotten, F. E. Bayon, E. Pagado, and P. Pamatian. Tertiary and quaternary magmatism in mindanao andleyte (philippines): geochronology, geochemistry and tectonic setting. *Journal of Asian Earth Sciences*, 15(2):121 – 153, 1997. ISSN 1367-9120. doi: https://doi.org/10.1016/S0743-9547(97)00002-0. URL http://www.sciencedirect.com/science/article/pii/S0743954797000020.
79. Ozawa, A., T. Tagami, E. L. Listanco, C. B. Arpa, and M. Sudo. Initiation and propagation of subduction along the philippine trench: evidence from the temporal and spatial distribution of volcanoes. *Journal of Asian Earth Sciences*, 23(1):105–111, 2004.
80. Puspito, N. T., Y. Yamanaka, T. Miyatake, K. Shimazaki, and K. Hirahara. Three-dimensional p-wave velocity structure beneath the indonesian region. *Tectonophysics*, 220(1-4):175–192, 1993.
81. Faccenna, C., A. F. Holt, T. W. Becker, S. Lallemand, and L. H. Royden. Dynamics of the ryukyu/izu-bonin-marianas double subduction system. *Tectonophysics*, 746:229–238, 2018.
82. Lallemand, S., Y. Font, H. Bijwaard, and H. Kao. New insights on 3-d plates interaction near taiwan from tomography and tectonic implications. *Tectonophysics*, 335(3-4):229–253, 2001.
83. Malavieille, J., S. E. Lallemand, S. Dominguez, A. Deschamps, C.-Y. Lu, C.-S. Liu, P. Schnurle, and A. S. Crew. Arc-continent collision in taiwan: New marine observations and tectonic evolution. *Special Papers-Geological Society of America*, pages 187–211, 2002.
84. Deschamps, A. and S. Lallemand. The west philippine basin: An eocene to early oligocene back arc basin opened between two opposed subduction zones. *Journal of Geophysical Research: Solid Earth*, 107(B12):EPM 1–1–EPM 1–24, 2002. doi: 10.1029/2001JB001706. URL https://agupubs.onlinelibrary.wiley.com/doi/abs/10.1029/2001JB001706.
85. Kizaki, K. Geology and tectonics of the ryukyu islands. *Tectonophysics*, 125(1-3):193–207, 1986.
86. Shinjo, R. Geochemistry of high mg andesites and the tectonic evolution of the okinawa trough–ryukyu arc system. *Chemical Geology*, 157(1-2):69–88, 1999.
87. Shinjo, R., M. Ban, K. Saito, and Y. Kato. K-ar dating of the volcanic rocks in the ryukyu arc. *Journal of Mineralogy, Petrology and Economic Geology*, 86(7):323–328, 1991. doi: 10.2465/ganko.86.323.
88. Eagles, G. The age and origin of the central scotia sea. *Geophysical Journal International*, 183(2):587–600, 2010.
89. Pearce, J., A. Hastie, P. Leat, I. W. Dalziel, L. A. Lawver, P. Barker, I. Millar, T. Barry, and R. Bevins. Composition and evolution of the ancestral south sandwich arc: Implications for the flow of deep ocean water and mantle through the drake passage gateway. *Global and Planetary Change*, 123:298–322, 2014.
90. Crameri, F. and P. Tackley. Spontaneous development of arcuate single-sided subduction in global 3-D mantle convection models with a free surface. *J. Geophys. Res. Solid Earth*, 119(7):5921–5942, July 2014. ISSN 2169-9356. doi: 10.1002/2014JB010939. URL http://dx.doi.org/10.1002/2014JB010939.
91. Barker, P. F. Scotia Sea regional tectonic evolution: implications for mantle flow and palaeocirculation. *Earth-Science Reviews*, 55(1–2):1–39, Oct. 2001. ISSN 0012-8252. URL http://www.sciencedirect.com/science/article/pii/S0012825201000551.
92. Barker, P. F. Tectonic framework of the east scotia sea. In *Backarc Basins*, pages 281–314. Springer, 1995.
93. Dalziel, I., L. Lawver, J. Pearce, P. Barker, A. Hastie, D. Barfod, H.-W. Schenke, and M. Davis. A potential barrier to deep Antarctic circumpolar flow until the late Miocene? *Geology*, 41(9):947–950, 09 2013. ISSN 0091-7613. doi: 10.1130/G34352.1. URL https://doi.org/10.1130/G34352.1.
94. Dalziel, I. W., L. A. Lawver, I. O. Norton, and L. M. Gahagan. The scotia arc: genesis, evolution, global significance. *Annual Review of Earth and Planetary Sciences*, 41, 2013.
95. Heine, C., R. D. Müller, C. Gaina, and P. Clift. Reconstructing the lost eastern tethys ocean basin: convergence history of the se asian margin and marine gateways. *Continent-Ocean Interactions Within East Asian Marginal Seas, Geophys. Monogr. Ser*, 149: 37–54, 2004.
96. Hall, R. Late jurassic–cenozoic reconstructions of the indonesian region and the indian ocean. *Tectonophysics*, 570:1–41, 2012.
97. Zahirovic, S., K. J. Matthews, N. Flament, R. D. Müller, K. C. Hill, M. Seton, and M. Gurnis. Tectonic evolution and deep mantle structure of the eastern tethys since the latest jurassic. *Earth-Science Reviews*, 162:293–337, 2016.
98. Smyth, H. R., R. Hall, and G. J. Nichols. Cenozoic volcanic arc history of east java, indonesia: the stratigraphic record of eruptions on an active continental margin. *Special Papers-Geological Society of America*, 436:199, 2008.
99. McCourt, W. J., M. J. Crow, E. J. Cobbing, and T. C. Amin. Mesozoic and cenozoic plutonic evolution of se asia: evidence from sumatra, indonesia. *Geological Society, London, Special Publications*, 106(1):321–335, 1996. ISSN 0305-8719. doi: 10.1144/GSL.SP.1996.106.01.21. URL https://sp.lyellcollection.org/content/106/1/321.

100. Crawford, A., S. Meffre, and P. Symonds. 120 to 0 ma tectonic evolution of the southwest pacific and analogous geological evolution of the 600 to 220 ma tasman fold belt system. *SPECIAL PAPERS-GEOLOGICAL SOCIETY OF AMERICA*, pages 383–404, 2003.
101. Whattam, S. A., J. Malpas, J. R. Ali, and I. E. Smith. New sw pacific tectonic model: Cyclical intraoceanic magmatic arc construction and near-coeval emplacement along the australia-pacific margin in the cenozoic. *Geochemistry, Geophysics, Geosystems*, 9(3), 2008.
102. Meffre, S., T. J. Falloon, T. J. Crawford, K. Hoernle, F. Hauff, R. A. Duncan, S. H. Bloomer, and D. J. Wright. Basalts erupted along the tongan fore arc during subduction initiation: Evidence from geochronology of dredged rocks from the tonga fore arc and trench. *Geochemistry, Geophysics, Geosystems*, 13(12), 2012.
103. van de Lagemaat, S. H., D. J. van Hinsbergen, L. M. Boschman, P. J. Kamp, and W. Spakman. Southwest pacific absolute plate kinematic reconstruction reveals major cenozoic tonga-kermadec slab dragging. *Tectonics*, 37(8):2647–2674, 2018.
104. Cooper, L. B., T. Plank, R. J. Arculus, E. H. Hauri, P. S. Hall, and S. W. Parman. High-ca boninites from the active tonga arc. *Journal of Geophysical Research: Solid Earth*, 115(B10), 2010.
105. McDougall, I. Dating of rhyolitic glass in the tonga forearc (hole 841b). In *Proceedings of the Ocean Drilling Program. Scientific results*, volume 135. Ocean Drilling Program, 1994.
106. Todd, E., J. B. Gill, and J. A. Pearce. A variably enriched mantle wedge and contrasting melt types during arc stages following subduction initiation in fiji and tonga, southwest pacific. *Earth and Planetary Science Letters*, 335:180–194, 2012.
107. Sibuet, J.-C. and S.-K. Hsu. How was taiwan created? *Tectonophysics*, 379(1-4):159–181, 2004.
108. Buchs, D. M., R. J. Arculus, P. O. Baumgartner, C. Baumgartner-Mora, and A. Ulianov. Late cretaceous arc development on the sw margin of the caribbean plate: Insights from the golfito, costa rica, and azuero, panama, complexes. *Geochemistry, Geophysics, Geosystems*, 11(7), 2010.
109. Galli-Olivier, C. Ophiolite and island-arc volcanism in costa rica. *Geological Society of America Bulletin*, 90(5):444–452, 1979.
110. Boschman, L. M., D. J. Van Hinsbergen, D. L. Kimbrough, C. G. Langereis, and W. Spakman. The dynamic history of 220 million years of subduction below mexico: A correlation between slab geometry and overriding plate deformation based on geology, paleomagnetism, and seismic tomography. *Geochemistry, Geophysics, Geosystems*, 19(12):4649–4672, 2018.
111. Gaina, C. and D. Müller. Cenozoic tectonic and depth/age evolution of the indonesian gateway and associated back-arc basins. *Earth-Science Reviews*, 83(3-4):177–203, 2007.
112. van Ufford, A. Q. and M. Cloos. Cenozoic tectonics of new guinea. *AAPG bulletin*, 89(1):119–140, 2005.
113. Hamilton, W. B. *Tectonics of the Indonesian region*. Number 1078. US Govt. Print. Off., 1979.
114. Milsom, J. New guinea and the western melanesian arcs. In *The ocean basins and margins*, pages 551–605. Springer, 1985.
115. Mpodozis, C. and V. Ramos. The andes of chile and argentina. *Geology of the Andes and its Relation to Hydrocarbon and Mineral Resources*, 1990.
116. Capitanio, F. A., C. Faccenna, S. Zlotnik, and D. R. Stegman. Subduction dynamics and the origin of andean orogeny and the Bolivian orocline. *Nature*, 480:83–86, 2011. ISSN 0028-0836. doi: 10.1038/nature10596. URL http://dx.doi.org/10.1038/nature10596.
117. Seton, M., R. D. Müller, S. Zahirovic, C. Gaina, T. Torsvik, G. Shephard, A. Talsma, M. Gurnis, M. Turner, S. Maus, and M. Chandler. Global continental and ocean basin reconstructions since 200Ma. *Earth-Science Reviews*, 113(3):212–270, July 2012. ISSN 0012-8252. doi: 10.1016/j.earscirev.2012.03.002. URL http://www.sciencedirect.com/science/article/pii/S0012825212000311.
118. Whittaker, J., R. Müller, G. Leitchenkov, H. Stagg, M. Sdrolias, C. Gaina, and A. Goncharov. Major australian-antarctic plate reorganization at hawaiian-emperor bend time. *Science*, 318(5847):83–86, 2007.
119. Gurnis, M., H. Van Avendonk, S. P. Gulick, J. Stock, R. Sutherland, E. Hightower, B. Shuck, J. Patel, E. Williams, D. Kardell, et al. Incipient subduction at the contact with stretched continental crust: The puysegur trench. *Earth and Planetary Science Letters*, 520:212–219, 2019.
120. Beccaluva, L., G. Macciotta, C. Savelli, G. Serri, and O. Zeda. Geochemistry and k/ar ages of volcanics dredged in the philippine sea (mariana, yap, and palau trenches and parece vela basin). *Washington DC American Geophysical Union Geophysical Monograph Series*, 23:247–268, 1980.
121. Ohara, Y., K. Fujioka, O. Ishizuka, and T. Ishii. Peridotites and volcanics from the yap arc system: implications for tectonics of the southern philippine sea plate. *Chemical Geology*, 189(1-2):35–53, 2002.
122. Zhang, Z., D. Dong, W. Sun, S. Wu, Y. Bai, X. Wang, and J. Fan. Subduction erosion, crustal structure, and an evolutionary model of the northern yap subduction zone: New observations from the latest geophysical survey. *Geochemistry, Geophysics, Geosystems*, 20(1):166–182, 2019.
