# Supplementary material for: A transdisciplinary and community-driven database to unravel subduction zone initiation
Source: Nat Commun. 2020 Jul 27;11:3750. doi: 10.1038/s41467-020-17522-9 (PMC7385650; doi:10.1038/s41467-020-17522-9)
Supplement: Supplementary file 2 — Description of Additional Supplementary Files [file 41467_2020_17522_MOESM2_ESM.pdf]

Description of Additional Supplementary Files

File Name: Supplementary Data 1
Description: Contains a collection of raw data, methodological settings and analyses concerning parameters that have been extracted from the global topological (full-plate) tectonic model of Müller et al.[48], the default plate model used in the construction of SZI database version 1.0.

[48] Müller, R. D. *et al.* Ocean Basin Evolution and Global-Scale Plate Reorganization Events Since Pangea Breakup. *Annu. Rev. Earth Planet. Sci.* **44**, 107-138, doi:10.1146/annurev-earth-060115- 012211 (2016).
